# miR-30d Inhibition Protects IPEC-J2 Cells Against *Clostridium perfringens* Beta2 Toxin-Induced Inflammatory Injury

**DOI:** 10.3389/fvets.2022.909500

**Published:** 2022-06-21

**Authors:** Kaihui Xie, Qiaoli Yang, Zunqiang Yan, Xiaoli Gao, Xiaoyu Huang, Pengfei Wang, Juanli Zhang, Jiaojiao Yang, Jie Li, Shuangbao Gun

**Affiliations:** ^1^College of Animal Science and Technology, Gansu Agricultural University, Lanzhou, China; ^2^Gansu Research Center for Swine Production Engineering and Technology, Lanzhou, China

**Keywords:** miR-30d, CPB2 toxin, IPEC-J2 cells, *PSME3*, inflammation, proliferation

## Abstract

*Clostridium perfringens* beta2 (CPB2) toxin, one of the virulence factors of *Clostridium perfringens* (*C. perfringens*), can cause necrotizing enterocolitis in piglets. Accumulating pieces of evidence indicate that microRNAs (miRNAs) refer to the regulation of inflammatory processes. Previously, we have discovered that miR-30d was differentially expressed between the ileum of normal piglets and *C. perfringens* type C-infected diarrheal piglets. Here, we found that miR-30d expression was lowered in CPB2 toxin-treated intestinal porcine epithelial cells (IPEC-J2) at different time points. Subsequently, we determined that miR-30d inhibitor attenuated CPB2 toxin revulsive inflammatory damage in IPEC-J2 cells and promoted cell proliferation and cell cycle progression, whereas miR-30d mimic had opposite results. In addition, we confirmed that Proteasome activator subunit 3 (*PSME3*) was a downstream target gene of miR-30d *via* a dual luciferase reporter assay, qPCR, and western blot. We also found that overexpression of *PSME3* suppressed CPB2 toxin-induced inflammatory damage and promoted cell proliferation and cycle progression. Our results demonstrate that miR-30d aggravates CPB2 toxin revulsive IPEC-J2 cells inflammatory injury *via* targeting *PSME3*, thereby providing a novel perspective for the prevention and treatment of piglet diarrhea at the molecular level.

## Introduction

Piglet diarrhea is one of the most common microbial diseases causing high mortality and serious economic losses (1). Piglet diarrhea is usually caused by bacterial pathogens, such as *Clostridia* (2), *Escherichia coli* (3), *Salmonella choleraesuis* (4), and *hyodysenteriae* (5). *Clostridium perfringens* (*C. perfringens*) is a zoonotic microorganism that causes necrotizing enteritis in animals, food poisoning in humans, and other digestive diseases (6, 7). *C. perfringens* can be grouped into A, B, C, D, E, F, and G 7 types according to its production of lethal toxins (8). Among them, *C. perfringens* type C is the pathogenic bacterium that causes pig enteritis, especially necrotizing enteritis in piglets, and has the characteristics of short course of disease and high lethality (9). The main virulence factors of *C. perfringens* type C are α (CPA), β1 (CPB1), and β2 (CPB2) toxins, all of which are highly virulent and have good immunogenicity (10). CPB2 toxin was first isolated from the culture supernatant of piglet-necrotizing enteritis disease material, and is a strongly cytotoxic and lethal toxin (11). Studies indicate that CPB2 is present in various animal intestinal disease *C. perfringens* isolates, causing absence of feces, diarrhea, and other symptoms (12, 13). In addition, CPB2 also has certain toxicity to cells. Zeng et al. (14) found that CPB2 toxin induced NCM460 human intestinal epithelial cell apoptosis. Gao et al. (15) and Luo et al. (16) showed that CPB2 toxin enhanced intestinal porcine epithelial cells (IPEC-J2) apoptosis, cytotoxicity, and inflammatory damage.

MicroRNAs (miRNAs) are highly conserved small non-coding RNAs, with a length of 18–25 nucleotides, which participate in post-transcriptional regulation by suppressing mRNA translation or inducing mRNA degradation (17, 18). miRNAs refer to the regulation of intestinal epithelial tight junction permeability, intestinal epithelial cell differentiation, barrier function, and intestinal mucosal immunity (19–21). Moreover, miRNAs also participate in the infection process of various pathogens. Wang et al. (22) found that miR-500 and miR-92b-3p regulated the process of *C. perfringens* type C infection in piglets. Sun et al. (23) indicated that miR-192 regulated *Escherichia coli* infection in piglets. Herrera et al. (24) found that miR-21 was upregulated in the mesenteric lymph nodes of *Salmonella*-infected weaned piglets.

Our previous miRNA high-throughput sequencing results indicated that miR-30d expression was differentially expressed in the ileum tissue of *C. perfringens* type C-infected with piglets diarrhea compared with that in normal piglets (22), implying that miR-30d may be a key molecule in the process of piglet diarrhea. miR-30d was firstly described in mouse tissue in 2002 (25), and belongs to the miR-30 miRNA family. miR-30d participates in multiple biological and pathological processes, including cellular proliferation, autophagy, differentiation, immune response, apoptosis, and inflammatory response (26–29). Liu et al. (30) indicated that miR-30d-5p facilitated oxidative stress injury in high glucose (HG)-treated Schwann cells *via* targeting *SIRT1*. Zhao et al. (31) indicated that miR-30d-5p expression was lowered in developing rat brains after Hypoxic–Ischemic (HI) injury, and enhanced apoptosis and aggravated brain injury after HI. Moreover, miR-30d was also found to be a potential therapeutic target for Porcine Reproductive and Respiratory Syndrome Virus (PRRSV) infection (32). However, the mechanism of action of miR-30d in *C. perfringens* type C-infected piglets and its downstream target molecules are unclear.

Proteasome activator subunit 3 (*PSME3*, also known as PA28 gamma, REG γ, or Ki antigen), belongs to the 11s proteasome activator family (33). *PSME3* refers to the regulation of biological processes, such as the cell cycle, proliferation, apoptosis, and immunodeficiency (34–37). In addition, *PSME3* also participates in the bacterial infection process. Moriishi et al. (38) demonstrated that *PSME3* promoted the degradation of hepatitis C virus (HCV) core protein. In the present study, we tested the expression of miR-30d in CPB2 toxin revulsive IPEC-J2 cells, and investigated the impacts of miR-30d by overexpression and silencing assays. The dual luciferase reporter assay, qPCR, and western blot verified the targeting relationship between miR-30d and *PSME3* to ascertain the regulatory effects of the miR-30d/PSME3 axis on CPB2 toxin treatment of IPEC-J2 cells.

## Materials and Methods

### Sample Collection

Seven-day-old Landrace- × -Yorkshire piglets (*n* = 30) were fed with a 1- × -10^9^ CFU/ml of *C. perfringens* type C medium to establish a *C. perfringens* type C-infected diarrhea piglet model. The specific method of model establishment has been described in our previous studies (2, 39). We collected the ileum tissue samples from piglets in the susceptible group infected with *C. perfringens* type C and the control group, and stored them at −80°C. All animal experiments followed the approval of the Ethical Committee of Experimental Animal Center of Gansu Agricultural University (Approval No. 2006-398).

### Cell Culture and Establishment of CPB2 Toxin Injury IPEC-J2 Cells Model

The IPEC-J2 and HEK-293T cell lines were obtained from BeNa Culture Collection (BNCC, Beijing, China). IPEC-J2 and HEK-293T cells were cultured in DMEM/F12 and DMEM mediums (HyClone, New York, NY, USA), containing 10% FBS (Everygreen, Hangzhou, China) and 1% penicillin and streptomycin (HyClone), respectively, and maintained at 37°C and 5% CO_2_.

We obtained recombinant CPB2 (rCPB2) toxin and determined that the optimal concentration of CPB2 toxin for IPEC-J2 cells was 20 μg/ml. The specific preparation and purification methods, and the choice of final concentration have been detailed described in our previous study (15, 16). In the current study, IPEC-J2 cells were treated with 20-μg/ml CPB2 toxin after transfection for 36 h to establish the cell damage model.

### Cell Transfection

IPEC-J2 cells were transfected at 70–80% confluence with miR-30d mimic (50 nM), mimic negative control (NC; 50 nM), miR-30d inhibitor (150 nM), inhibitor NC (150 nM; GenePharma, Shanghai, China); pcDNA3.1 (+) vector (1 μg; Promega, Madison, WI, USA), *PSME3* overexpression vector (pc-PSME3; 1 μg; GENEWIZ, Suzhou, China), si-NC or si-PSME3 (150 nM; GenePharma) using Lipofectamine™ 2000 reagent (Invitrogen, Carlsbad, CA, USA) according to the manufacturer's guidance. HEK-293T cells were co-transfected at 70–80% confluence with the miR-30d mimic or mimic NC and pmirGLO-*PSME3* 3' untranslated region (UTR)-wild type (WT) or mutation (Mut; 1 μg). miR-30d mimic, inhibitor, si-PSME3, and their respective NCs sequences are exhibited in Supplementary Table 1.

### Enzyme-Linked Immuno Sorbent Assay

Pro-inflammatory cytokines [interleukin 1β (IL-1β), IL-6, and IL-8] levels in CPB2 toxin-induced IPEC-J2 cells were assessed by Enzyme-linked immuno sorbent assay (ELISA) kits (mlbio, Shanghai, China). IPEC-J2 cells were fostered in 24-well plates. After transfection and treatment with CPB2 toxin for 36 h, the cell supernatant was collected and centrifuged at 2,000 rpm for 20 min for detection according to the manufacturer's instructions. Meanwhile, blank and standard wells were set, and the absorbance was tested at 450 nm with a microplate reader (Thermo Fisher Scientific, Waltham, MA, USA).

### Detection of Lactate Dehydrogenase Activity and Reactive Oxygen Species Level

An lactate dehydrogenase (LDH) assay kit (APPLYGEN, Beijing, China) was employed to assess IPEC-J2 cells cytotoxicity. The cell supernatant was collected and tested according to the manufacturer's instructions. Conclusively, the absorbance was tested at 450 nm in the microplate reader (Thermo Fisher Scientific). For reactive oxygen species (ROS) level detection, IPEC-J2 cells were cultured 24-well plates. After the IPEC-J2 cells were transfected and treated with CPB2 toxin, 200 μL of Dichloro-dihydro-fluorescein diacetate (DCFH-DA) probe (Beyotime, Shanghai, China) diluted 1:1,000 with a serum-free cell culture medium was appended to each well and incubated at 37°C for 20 min. Subsequently, the absorbance was observed with a microplate reader at 488 nm excitation wavelength and 535 nm emission wavelength (Thermo Fisher Scientific).

### Detection of Cell Viability

Cell viability was appraised with Cell Counting Kit-8 (CCK-8; Solarbio, Beijing, China). IPEC-J2 cells were fostered in 96-well plates, and then transfected with miR-30d mimic, inhibitor, and their respective NCs. After CPB2 toxin disposed cells for 36 h, 10 μL of CCK-8 reagent was appended to cells and hatched for 2 h. A microplate reader (Thermo Fisher Scientific) was performed to detect the absorbance at 450 nm.

### 5-Ethynyl-2'-Deoxyuridine Staining

The Beyoclick™ 5-Ethynyl-2'-Deoxyuridine (EdU)-555 cell proliferation detection kit (Beyotime) was employed to ascertain the proliferation of IPEC-J2 cells. The cells were fostered in 24-well plates for 24 h and then transfected. After 36 h of CPB2 toxin treatment, the cells were incubated with an equal volume of 2 × EdU (250 μL) and a culture medium (250 μL) for 2 h. Then, the cells were stained following the manufacturer's specifications and viewed under a fluorescence microscope (Olympus IX71, Tokyo, Japan).

### Flow Cytometry for Cell Cycle

Flow cytometry was performed to analyze the cell cycle. After the IPEC-J2 cells were transfected and CPB2 toxin treatment for 36 h, centrifugated at 1,500 rpm for 5 min to collect the cells, 1 ml of 75% ethanol was added to cells and incubated overnight at 4°C. Nextly, the cells were centrifuged for 5 min at 1,500 rpm, the supernatant was discarded, and 100-μL PBS was appended to resuspend the cells. Subsequently, 2 μL (10 mg/ml) of RNase A (Solarbio) was added to the cells, and the cells were fostered at 37°C for 30 min. Finally, 100 μL (100 μg/ml) of propidium iodide (PI) staining solution (Servicebio, Wuhan, China) was added to the cells, and the cells were stained for 10 min in the dark. A flow cytometer (Beckman Coulter, Indianapolis, IN, USA) was employed to detect the number of the cells at different stages of the cell cycle.

### Real-Time Quantitative PCR Analysis

Total RNA was extracted with Trizol reagent (TransGen Biotech, Beijing, China). RNA was reversed transcription into complementary DNA (cDNA) for miRNA and mRNA expression analysis by miRNA First-Strand cDNA Synthesis Kit (Takara, Dalian, China) and *Evo M-MLV* RT Kit (Accurate Biotech, Changsha, China), respectively, following the manufacturer's recommendations, and cDNA was stored at −20°C. Two × SYBR Green qPCR Master Mix (Servicebio) was used for real-time quantitative PCR (qPCR). *U6* and *GAPDH* were considered internal references for miRNA and mRNA, respectively. Gene expression levels were normalized using the 2 ^−(ΔΔCt)^ method (40). The primers were compounded by GENEWIZ Biotechnology Co., Ltd. The primer sequences are displayed in Table 1.

**Table 1:** The primers used in this research.

**Gene**	**Primer Sequence (5^′^-3^′^)**	**Accession No**.	**Utilization**
miR-30d	Forward: TAAACATCCCCGACTGGAAGCT	MIMAT0013871	qPCR
	Reverse: mRQ 3' Primer (TaKaRa)		
*U6*	Forward: GGAACGATACAGAGAAGATTAGC	NC_000015	qPCR
	Reverse: TGGAACGCTTCACGAATTTGCG		
*PSME3*	Forward: CCG*CTCGAG*GCTCAAGACCGACATTGCCTT	XM_005668789.3	3'UTR amplification
	Reverse: ACGC*GTCGAC*AGTACTCCAGAAATTAGGAC		
*PSME3*	Forward: ATCGTGATGGGGAAACTGGC	XM_005668789.3	qPCR
	Reverse: AACATCCTGCGCACACAAAC		
*CDK4*	Forward: ATGTGGAGCGTTGGCTGTAT	NM_001123097.1	qPCR
	Reverse: TGCTCCAGACTCCTCCATCT		
*PCNA*	Forward: GCCACTCCACTCTCTCCTAC	NM_001291925.1	qPCR
	Reverse: GCATCACCGAAGCAGTTCTC		
*p21*	Forward: ACGTCTCAGGAGGACCATGT	XM_013977858.2	qPCR
	Reverse: CGGCGTTTGGAGTGGTAGAA		
*GAPDH*	Forward: AGTATGATTCCACCCACGGC	NM_001206359.1	qPCR
	Reverse: TACGTAGCACCAGCATCACC		

*Bases in italics are restriction enzyme sites*.

### Western Blot

A RIPA lysis buffer (Solarbio) was employed to withdraw the total protein, and the BCA Protein Assay Kit (Beyotime) was utilized to detect protein concentration. Protein was subjected to 12% sodium dodecyl sulfate-polyacrylamide gel electrophoresis (SDS-PAGE) and then shifted to polyvinylidene fluoride (PVDF) membrane. The membrane was closured with 5% skimmed milk at room temperature for 30 min. Then, the membrane was fostered overnight at 4°C with the anti-PSME3 (1:800; bs-4222R, Bioss, Beijing, China) and anti-β-actin (1:1,000; bs-0061R, Bioss) antibodies. Subsequently, the membrane was fostered with a goat anti-rabbit horseradish peroxidase (HRP) IgG antibody (1:1,000; bs-0295G-HRP, Bioss) for 30 min at room temperature. The immunoreactive protein bands were normalized by an Enhanced Chemiluminescence (ECL) Kit (NCM Biotech, Suzhou, China), and Image J software (National Institutes of Health, USA) analyzed the gray values.

### Plasmid Construction

TargetScan (http://www.targetscan.org/vert_72/) was utilized to predict the binding site of miR-30d with *PSME3*. The *PSME3* 3'UTR fragment (XM_005668789.3), containing the miR-30d binding site, was amplified *via* PCR using specific primers containing *Xho* I and *Sal* I (Takara) restriction enzyme sites (Table 1). After recovery and purification, the fragment was inserted into the pmirGLO fluorescent vector (Promega) to construct the *PSME3* 3'UTR-WT vector. The sequence TGTTTAC, which binds to miR-30d in *PSME3* 3'UTR, was mutated to ACAAATG, and the *PSME3* 3'UTR-Mut vector and the pc-PSME3 vector were synthesized by GENEWIZ. The recombinant plasmids were verified by sequencing and double enzyme digestion.

### Dual Luciferase Reporter Assay

miR-30d mimic and mimic NC were co-transfected with *PSME3* 3'UTR-WT or Mut vectors, respectively, into the HEK-293T cells. After 48 h transfection, a dual luciferase reporter assay System (Promega) was executed to monitor luciferase activity according to the manufacturer's guidance. Renilla luciferase was utilized as a reporter gene for normalization control to evaluate the dual luciferase reporter data.

### Statistical Analysis

All assays were repeated independently at least three times. SPSS 18.0 (IBM Corp., Armonk, NY, USA) was executed to analyze the data. All the data in this study are expressed as mean ± SD. Student's *T*-test was performed to analyze differences between the two groups. ^*^p < 0.05 was implied statistically significant, and ^**^p < 0.01 was considered extremely significant.

## Results

### miR-30d Expression Analysis

**Figure 1:**
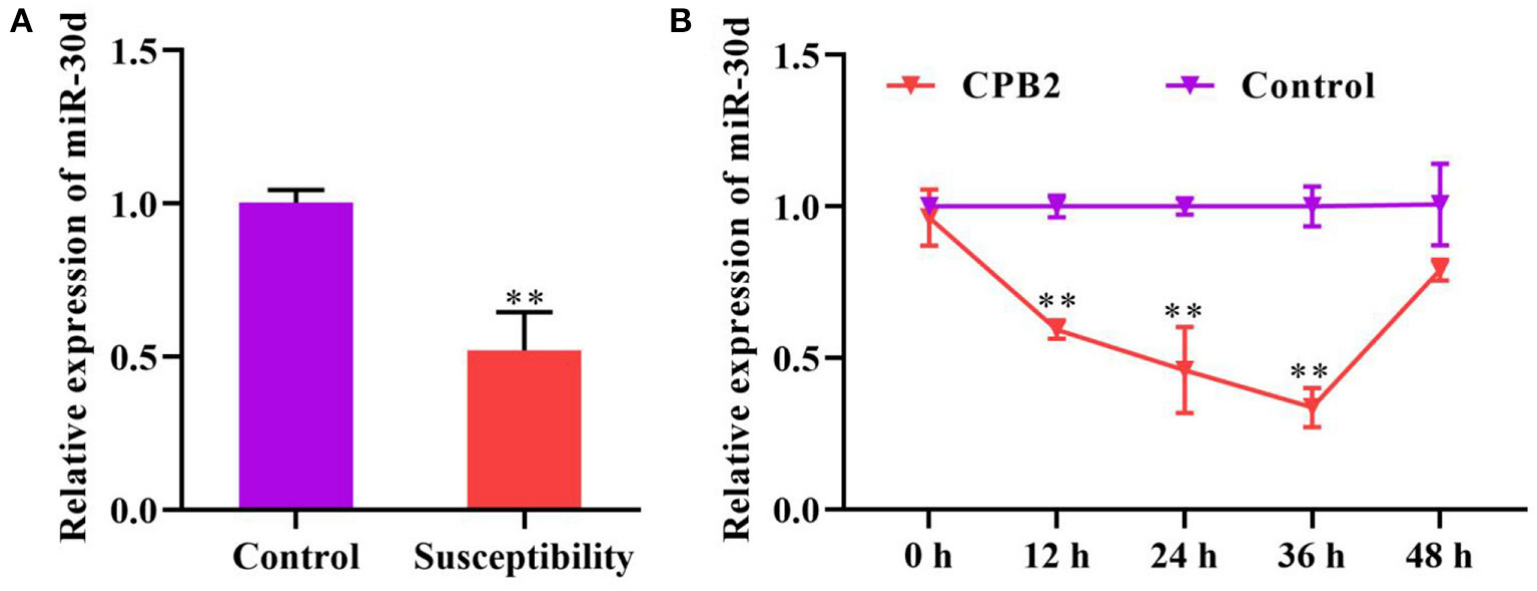
miR-30d expression reduced in *Clostridium perfringens* type C-infected piglets and CPB2 toxin revulsive IPEC-J2 cells. **(A)** The expression of miR-30d in the ileum tissue of *Clostridium perfringens* type C-infected diarrhea piglets was evaluated *via* qPCR. **(B)** qPCR analysis of the expression of miR-30d in IPEC-J2 cells treated with CPB2 toxin at 12, 24, 36, and 48 h. ** *p* < 0.01.

We monitored the expression of miR-30d in the ileum of *C. perfringens* type C-infected piglets with diarrhea and normal piglets, and discovered that miR-30d expression was notably reductive in the piglets with diarrhea (Figure 1A). Subsequently, we evaluated the expression of miR-30d at 12, 24, 36, and 48 h after CPB2 toxin treatment of the IPEC-J2 cells. As exhibited in Figure 1B, miR-30d expression was lowered in the CPB2 toxin revulsive IPEC-J2 cells, and its downregulation was most significant at 36 h. These data suggest that miR-30d might be involved in the infection of *C. perfringens* type C.

### miR-30d Aggravates the CPB2 Toxin-Induced Inflammatory Damage in IPEC-J2 Cells

**Figure 2:**
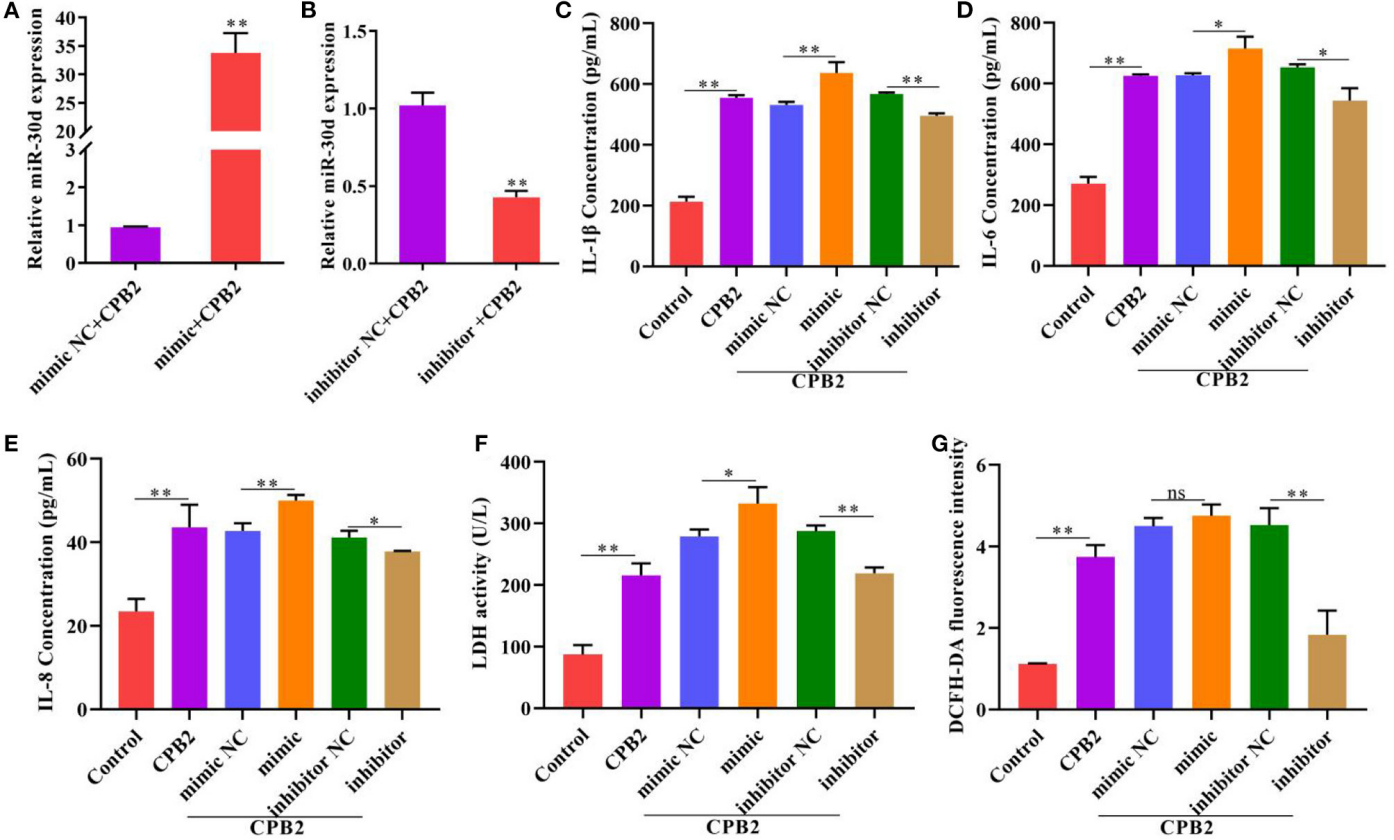
Downregulation of miR-30d attenuated CPB2 toxin-induced inflammatory damage. IPEC-J2 cells were transfected with mimic NC, miR-30d mimic (50 nM), inhibitor NC, and miR-30d inhibitor (150 nM), and treated with CPB2 toxin. **(A,B)** miR-30d expression was detected at 36 h after transfection by qPCR. **(C–E)** ELISA was performed to assess the levels of IL-1β, IL-6, and IL-8 after transfection and treatment with CPB2 toxin for 36 h. **(F)** LDH activity was detected at 450 nm in a microplate reader. **(G)** The ROS level was evaluated by a microplate reader at 488-nm excitation wavelength and 535-nm emission wavelength. ns, not significant, *p < 0.05, **p < 0.01.

To probe the impact of miR-30d on the inflammatory process, we overexpressed and knocked down miR-30d in the CPB2-induced IPEC-J2 cells. The results indicated that, after transfection with the miR-30d mimic, the level of miR-30d was notably enhanced, and transfection with the miR-30d inhibitor decreased the levels of miR-30d (Figures 2A,B). The ELISA assay indicated that the miR-30d mimic signally augmented the levels of IL-1β, IL-6, and IL-8, while the miR-30d inhibitor repressed the levels of IL-1β, IL-6, and IL-8 (Figures 2C–E). Moreover, after CPB2 treatment of the IPEC-J2 cells, LDH activity and the ROS level significantly increased. And transfection of the miR-30d mimic further enhanced CPB2 toxin-induced LDH activity, but not significant in the ROS level. By contrast, transfection of the miR-30d inhibitor remarkably decreased the LDH activity and the ROS level (Figures 2F,G). These data demonstrate that miR-30d promoted the CPB2 toxin revulsive inflammatory injury in the IPEC-J2 cells.

### miR-30d Suppresses the Proliferation in CPB2 Toxin-Induced IPEC-J2 Cells

**Figure 3:**
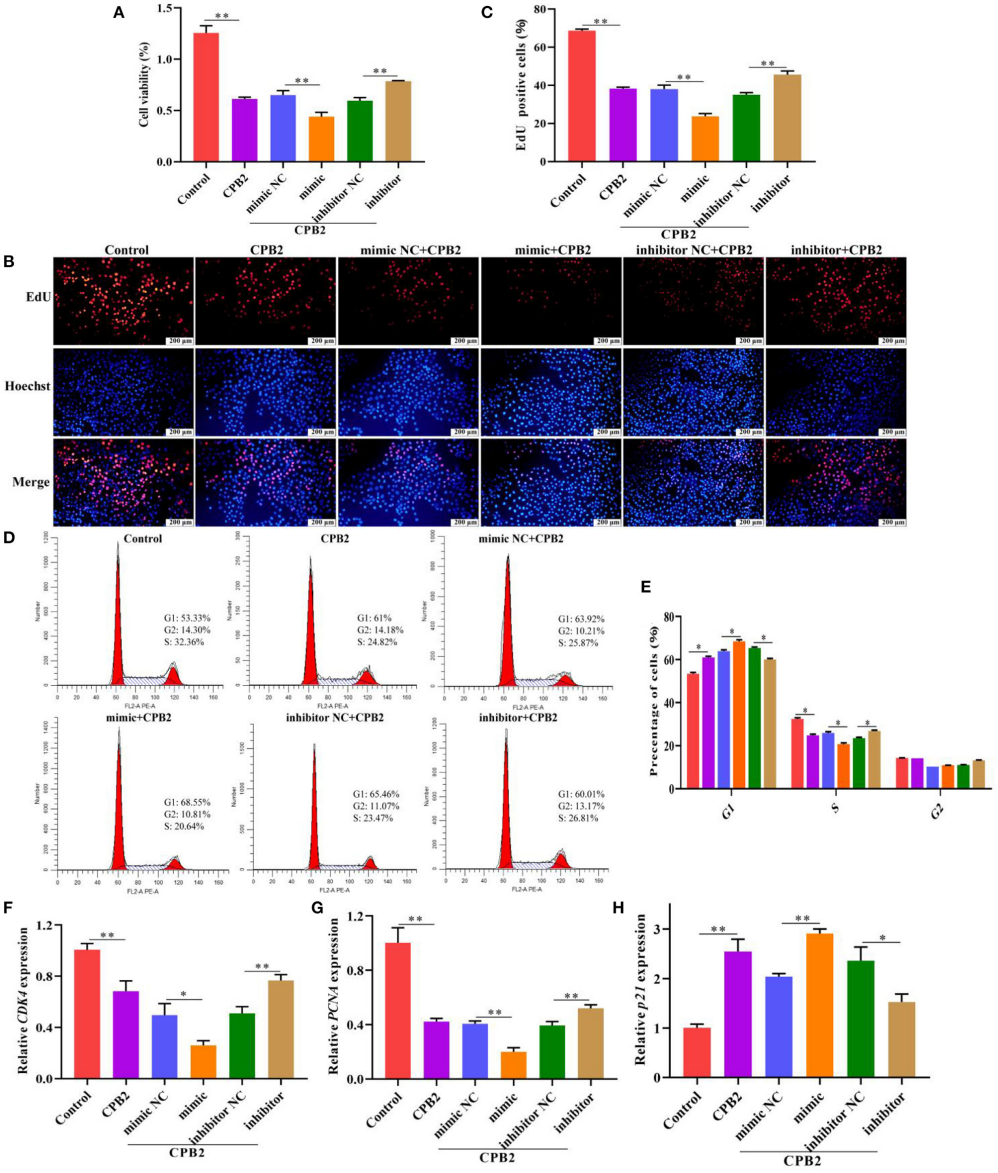
miR-30d arrested cell proliferation and cycle progression in CPB2 toxin revulsive IPEC-J2 cells. **(A–H)** The IPEC-J2 cells were transfected with mimic NC, miR-30d mimic (50 nM), inhibitor NC, and miR-30d inhibitor (150 nM), and treated with CPB2 toxin for 36 h. **(A)** CCK-8 was performed to analyze cell viability, and the results were recorded using a microplate reader at 450 nm. **(B)** The EdU assay was performed. The IPEC-J2 cells in S-phase were stained red with EdU, whereas the nucleus was dyed blue with Hoechst. **(C)** Image J analysis of the proportion of proliferating cells. **(D,E)** Flow cytometry analysis of the IPEC-J2 cell cycle. **(F–H)** qPCR analysis of *CDK4, PCNA*, and *p21* expression. *p < 0.05, **p < 0.01.

To inquire the impact of miR-30d on CPB2 toxin-induced cell viability and proliferation in the IPEC-J2 cells, we used CCK-8 assay, EdU, flow cytometry, and qPCR to assess after transfection and CPB2 toxin treatment. Obviously, CPB2 toxin weakened cell viability and lowered the number of positive proliferating cells. Moreover, overexpression of miR-30d promoted the CPB2 toxin-induced suppression of cell viability and proliferation, whereas knockdown of miR-30d significantly increased cell viability and proliferation (Figures 3A–C). We performed flow cytometry and qPCR to reveal the roles of miR-30d in the cell cycle. The results indicated that G1 transition to the S phase was blocked in the CPB2 toxin-treated IPEC-J2 cells compared with that in normal cells (Figures 3D,E). Overexpression of miR-30d further arrested the G1/S transition, whereas silencing miR-30d facilitated cell cycle progression (Figures 3D,E). Furthermore, the expression levels of cell cycle marker genes, including cyclin-dependent kinase 4 (*CDK4*) and proliferating cell nuclear antigen (*PCNA*), were notably lowered in the CPB2 toxin-induced IPEC-J2 cells, and the cyclin-dependent kinase inhibitor 1A (*p21*) level increased. *CDK4* and *PCNA* levels reduced, and the *p21* level aggrandized in the miR-30d mimic group, whereas the miR-30d inhibitor group exhibited the opposite results (Figures 3F–H). The above results manifest that miR-30d suppressed cell proliferation and cycle progression in the CPB2 toxin revulsive IPEC-J2 cells.

### *PSME3* Is a Regulatory Target of miR-30d

**Figure 4:**
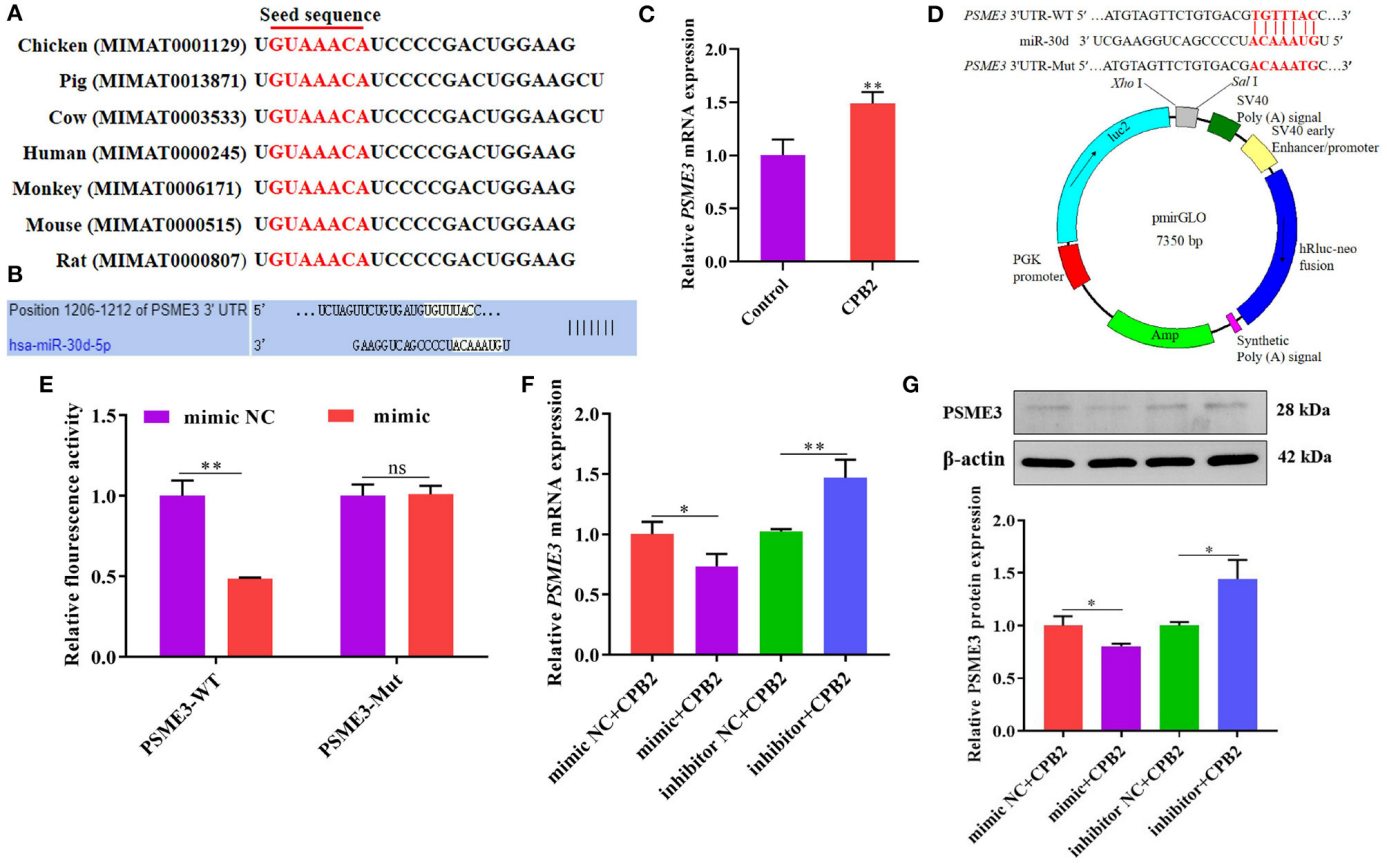
miR-30d negatively regulated the expression of *PSME3*. **(A)** The matured sequence of miR-30d was aligned in chicken, pig, cow, human, monkey, mouse, and rat. **(B)** TargetScan analyzed the binding site of miR-30d to *PSME3*. **(C)** qPCR analysis of *PSME3* expression in CPB2 toxin revulsive IPEC-J2 cells. **(D)** Comparison of the *PSME3* 3'UTR-WT and Mut vectors. **(E)** The relative luciferase activity of the IPEC-J2 cells after co-transfected with miR-30d mimic or mimic NC and *PSME3* 3'UTR-WT or Mut. **(F,G)** The miR-30d mimic and the inhibitor were transfected into IPEC-J2 for 36 h, **(F)** the mRNA level of *PSME3* was evaluated through qPCR, **(G)** the protein level of PSME3 was evaluated *via* western blot. ns: not significant, *p < 0.05, **p < 0.01.

Through the sequence alignment of miR-30d in various species, we discovered that the matured sequence of miR-30d was conserved in chicken, pig, cow, human, monkey, mouse, and rat (Figure 4A), indicating that the function of miR-30d is likely to be conserved. miRNA target prediction analysis revealed that the *PSME3* 3' UTR and miR-30d had binding sites, which manifested that *PSME3* might be a potential target gene of miR-30d (Figure 4B). We found that the *PSME3* level enhanced in the CPB2 toxin revulsive IPEC-J2 cells (Figure 4C). To further confirm the targeting relationship between miR-30d and *PSME3*, we constructed *PSME3* 3'UTR-WT and Mut vectors (Figure 4D). As expected, miR-30d mimic signally reduced the luciferase activity of *PSME3* 3'UTR-WT, while the luciferase activity of *PSME3* 3'UTR-Mut did not change significantly (Figure 4E). Next, we used qPCR and western blot to verify whether miR-30d negatively regulates the expression of *PSME3*. As displayed in Figures 4F,G, the expression levels of PSME3 mRNA and protein remarkably lessened in the mimic group as compared with those in the mimic NC group, whereas the miR-30d inhibitor increased the level of PSME3. The above results demonstrate that *PSME3* is the direct target gene of miR-30d.

### *PSME3* Alleviates the CPB2 Toxin Revulsive Inflammatory Damage in IPEC-J2 Cells

**Figure 5:**
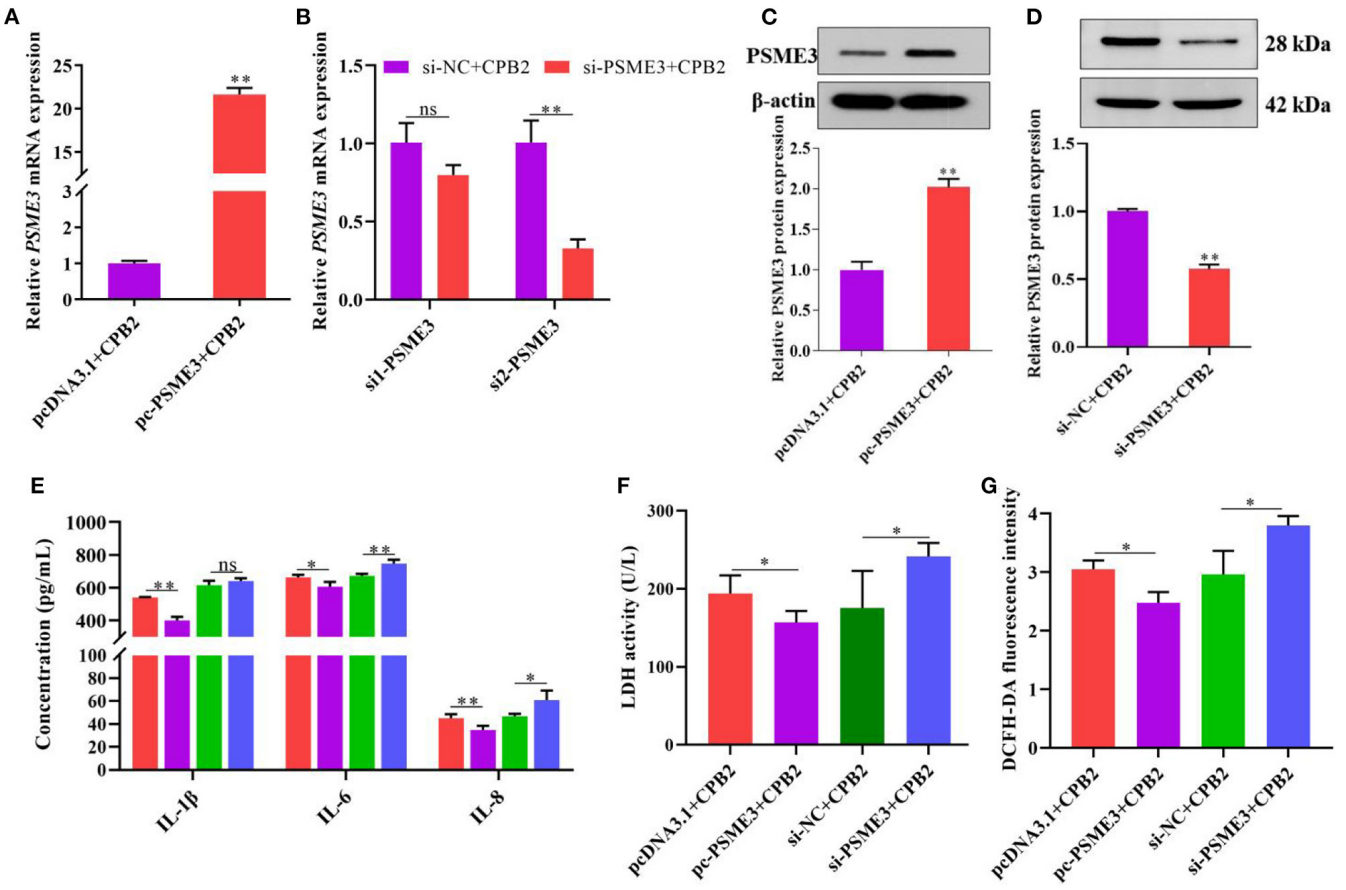
*PSME3* repressed CPB2 toxin-induced inflammation in IPEC-J2 cells. The IPEC-J2 cells were transfected with pcDNA3.1, pc-PSME3 (1 μg), si-NC, and si-PSME3 (150 nM), and treated with CPB2 toxin. **(A,B)** qPCR analysis of *PSME3* expression after transfection of pcDNA3.1, pc-PSME3 (1 μg), si-NC, si1-PSME3, and si2-PSME3 (150 nM). **(C,D)** Western blot analysis of PSME3 expression after transfection of pcDNA3.1, pc-PSME3 (1 μg), si-NC, and si2-PSME3 (si-PSME3, 150 nM). **(E)** ELISA analysis of IL-1β, IL-6, and IL-8 expression. **(F)** LDH activity was tested at 450 nm in a microplate reader. **(G)** The ROS level was tested by microplate reader at 488-nm excitation wavelength and 535-nm emission wavelength. ns: not significant, *p < 0.05, **p < 0.01.

To redouble explore the impact of *PSME3* in CPB2 toxin revulsive IPEC-J2 cells inflammation, we overexpressed and knocked down *PSME3* in the IPEC-J2 cells. The results manifested that, after transfection of pc-PSME3, the expression of PSME3 mRNA and protein dramatically increased (Figures 5A,C). We designed *PSME3* siRNA to knockdown. As shown in Figure 5B, si2-PSME3 (si-PSME3) successfully decreased the expression of *PSME3*. Subsequently, we tested the expression of PSME3 protein after transfection with si-PSME3, which displayed that si-PSME3 reduced the PSME3 protein level (Figure 5D). Moreover, we found that the overexpression of *PSME3* notably repressed the expression of IL-1β, IL-6, and IL-8, and the knockdown of *PSME3* showed the opposite results (Figure 5E). Meanwhile, the LDH activity and ROS levels significantly decreased after the overexpression of *PSME3*, and the LDH activity and the ROS levels were higher after the knockdown of *PSME3* (Figures 5F,G). In summary, these data reveal that *PSME3* weakened CPB2 toxin revulsive inflammatory damage in the IPEC-J2 cells.

### *PSME3* Facilitates Cell Proliferation and Cell Cycle Progression in CPB2 Toxin-Infected IPEC-J2 Cells

**Figure 6:**
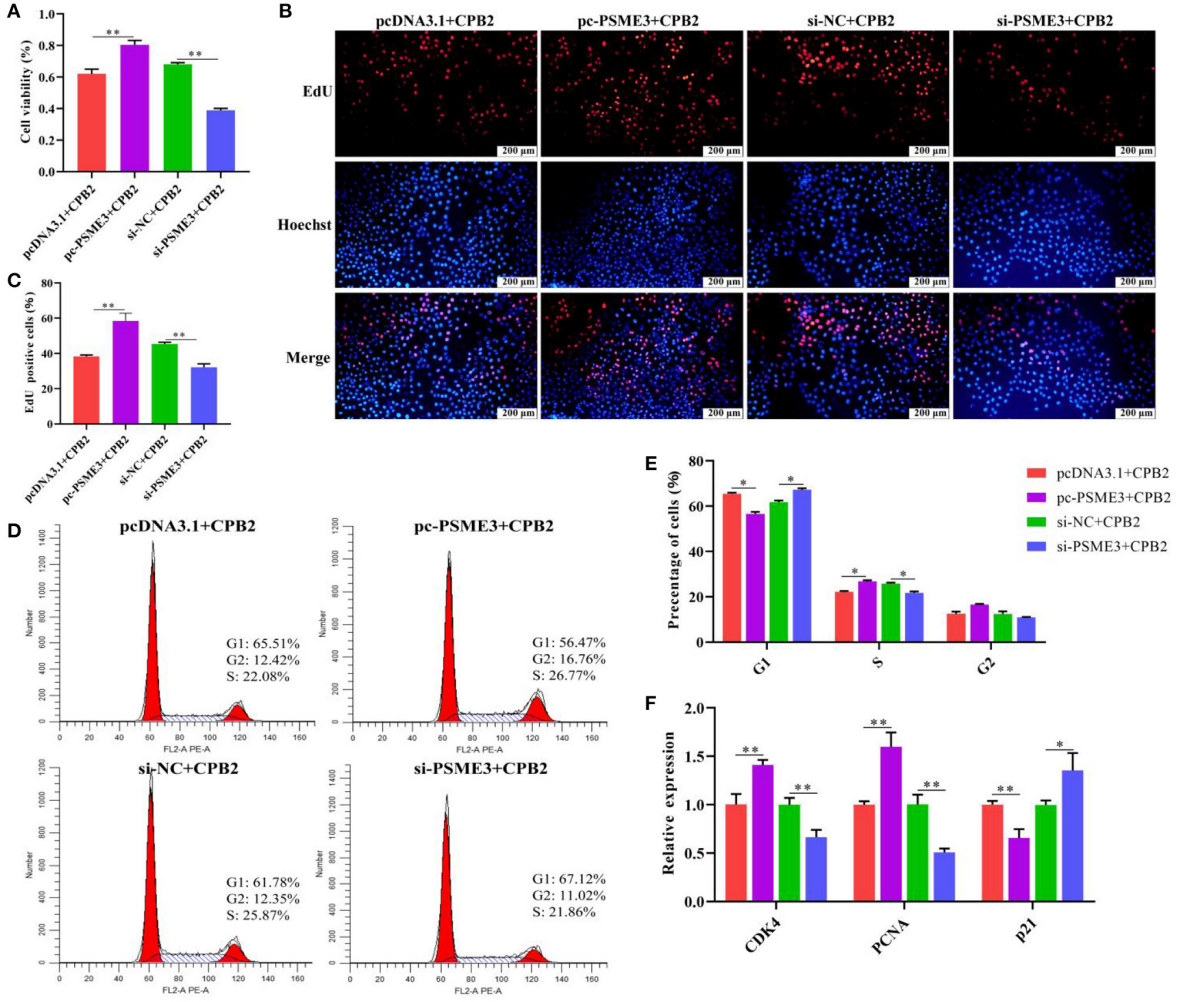
*PSME3* promoted cell proliferation and cycle progression in the CPB2 toxin revulsive IPEC-J2 cells. **(A–F)** The IPEC-J2 cells were transfected with pcDNA3.1, pc-PSME3 (1 μg), si-NC, and si-PSME3 (150 nM), and treated with CPB2 toxin for 36 h. **(A)** Cell viability was tested *via* CCK-8 on a microplate reader at 450 nm. **(B)** The EdU assay was performed. The IPEC-J2 cells in S-phase were stained red with EdU, whereas the nucleus was dyed blue with Hoechst. **(C)** Image J analysis of the proportion of proliferating cells. **(D,E)** Flow cytometry analysis of the IPEC-J2 cell cycle. **(F)** qPCR analysis of *CDK4, PCNA*, and *p21* expression. *p < 0.05, **p < 0.01.

We executed CCK-8, EdU, flow cytometry, and qPCR assays to evaluate cell viability, proliferation, and cell cycle. The CCK-8 results demonstrated that the cell viability was notably enhanced after transfection of pc-PSME3, while cell viability was weakened after transfection si-PSME3 (Figure 6A). Moreover, pc-PSME3 transfection dramatically aggrandized the number of red EdU-positive cells as compared with that in the pcDNA3.1 control group, whereas transfection with si-PSME3 lowered red EdU-positive cells (Figures 6B,C). Furthermore, flow cytometry showed that upregulation of *PSME3* promoted the transition from G1 to S phases in the CPB2 toxin revulsive IPEC-J2 cells, whereas downregulation of *PSME3* arrested G1 to S transition (Figures 6D,E). We also found that overexpression *PSME3* remarkably enhanced the levels of *CDK4* and *PCNA*, and reduced the expression of *p21* (Figure 6F), whereas the *PSME3* knockdown had the opposite results. Thus, *PSME3* accelerated the proliferation in the CPB2 toxin-induced IPEC-J2 cells.

### miR-30d Targets *PSME3* to Modulate CPB2 Toxin-Induced Inflammatory Injury in IPEC-J2 Cells

**Figure 7:**
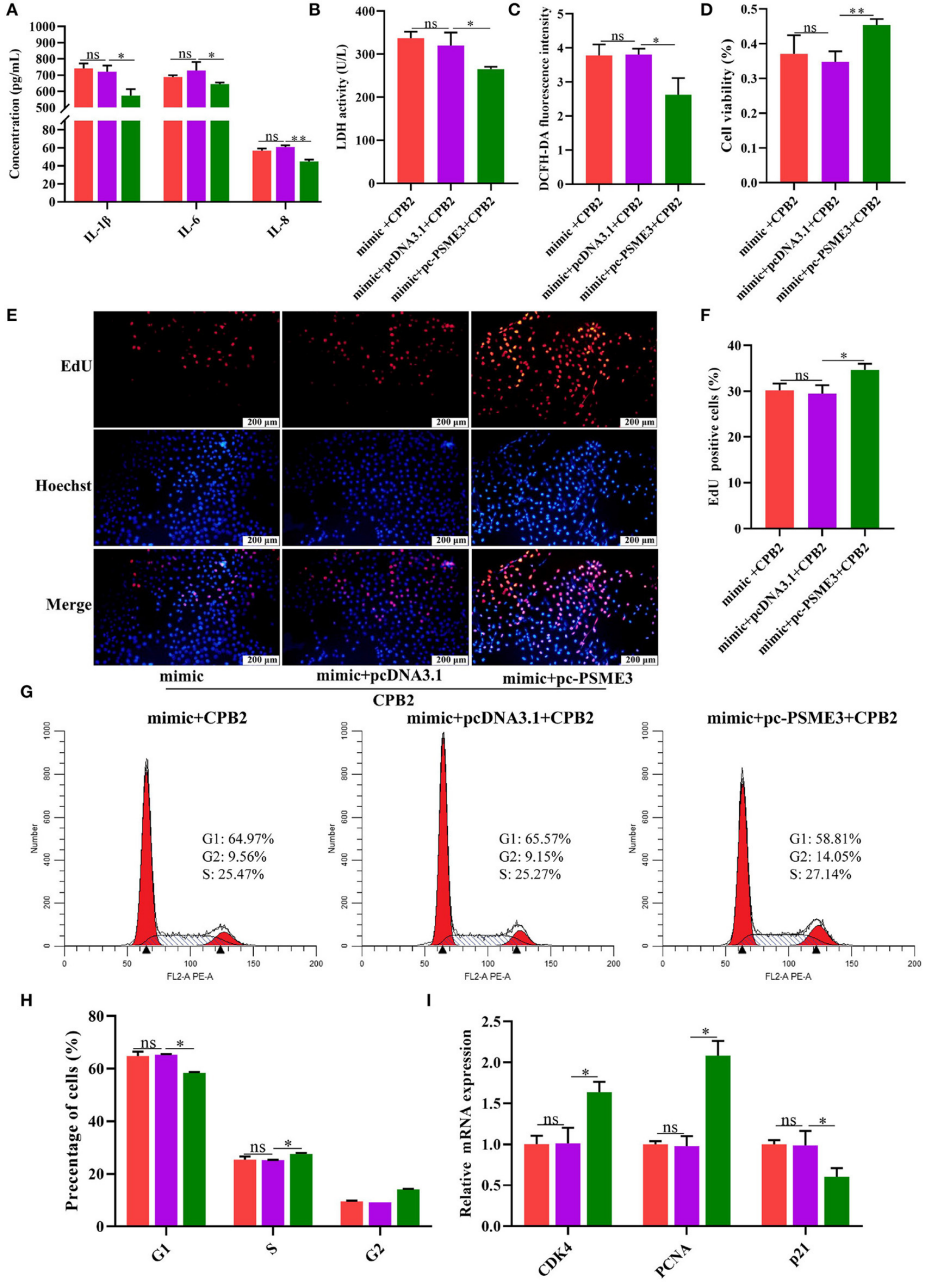
*PSME3* reversed the promotion of inflammatory damage by miR-30d in the IPEC-J2 cells. **(A–I)** The IPEC-J2 cells were co-transfected with the miR-30d mimic and the pc-PSME3 vector and treated with CPB2 toxin for 36 h. **(A)** ELISA analysis of IL-1β, IL-6, and IL-8 levels. **(B)** LDH activity was detected at 450 nm in microplate reader. **(C)** The ROS level was detected by a microplate reader at 488-nm excitation wavelength and 535-nm emission wavelength. **(D)** Cell viability *via* CCK-8. **(E)** The EdU assay was performed. The IPEC-J2 cells in S-phase were stained red with EdU, whereas the nucleus was dyed blue with Hoechst. **(F)** Image J analysis of the proportion of proliferating cells. **(G,H)** Flow cytometry evaluated the cell cycle. **(I)** qPCR analysis of *CDK4, PCNA*, and *p21* expression. ns, not significant,*p < 0.05, **p < 0.01.

To further verify whether miR-30d regulates CPB2 toxin-induced IPEC-J2 cells inflammatory damage *via* targeting *PSME3*, we co-transfected the IPEC-J2 cells with the miR-30d mimic and pc-PSME3 vector, and treated with CPB2 toxin. Compared with the mimic+pcDNA3.1 group, the mimic+pc-PSME3 group significantly reduced the levels of IL-1β, IL-6, IL-8, LDH, and ROS (Figures 7A–C). CCK-8, EdU, and Flow cytometry results indicated that overexpression of *PSME3* attenuated the inhibitory effect of the miR-30d mimic on cell viability, proliferation, and the cell cycle (Figures 7D–H). Moreover, co-transfection of the miR-30d mimic and pc-PSME3 significantly promoted the expression of *CDK4* and *PCNA*, and suppressed the expression of *p21* compared to transfection of the miR-30d mimic and the pcDNA3.1 vector (Figure 7I). The above results suggest that *PSME3* reversed the promotion of inflammatory damage by the miR-30d in IPEC-J2 cells.

## Discussion

Increasing studies have manifested that miRNAs regulate the process of pathogens infection livestock and poultry. For example, chicken miR-1306-5p targeted *Tollip* and modulated host defense against *Salmonella Enteritidis* infection (41). miR-29b inhibited bovine viral diarrhea virus (BNDV) replication in Madin-Darby bovine kidney (MDBK) cells by targeting *caspase-7* and *NAIF* (42). miR-218 targeted *SOCS3* to regulate PRRSV replication (43). miR-129a-3p significantly downregulated in porcine epidemic diarrhea virus (PEDV)-infected IPEC-J2 cells and targeted the EDA-mediated NF-κB pathway to suppress PEDV replication (44). Moreover, Gao et al. (45) demonstrated that epigenetic upregulation of ssc-miR-124a attenuated apoptosis and inflammation in the CPB2 toxin-infected IPEC-J2 cells. miR-140-5p targeting *VEGFA* exacerbated CPB2 toxin-induced inflammatory injury in the IPEC-J2 cells *via* ERK1/2 and JNK signaling pathways (46). Our previous study has revealed that miR-30d was significantly downregulated in the ileum tissue of *C. perfringens* type C-infected diarrhea piglets, implying that miR-30d might have a vital role in this process. In the present research, we found that miR-30d expression decreased after CPB2 toxin treatment of the IPEC-J2 cells, and the expression was lowest at 36 h, which further indicated that miR-30d refers to regulate the process of *C. perfringens* infection in the piglets.

Studies indicated that the miR-30 family plays a pivotal role in the inflammatory response process. miR-30a attenuated Hepatitis B Virus X Protein revulsive autophagosome formation in hepatic cells (47). miR-30b and miR-30c repressed the replication of Hepatitis C Virus (48). The current pieces of research on miR-30d has mainly focused on cancer, such as prostate cancer (49), lung cancer (50), and colon cancer (51). Moreover, miR-30d was a potential therapeutic target for PRRSV infection (32). miR-30d suppressed the replication of infectious bronchitis virus (IBV) *via* targeting *USP47* (52). Selenium deficiency induced oxidative stress and inflammation in pig adrenal tissue *via* the miR-30d-R_1/TLR4 pathway (53). In the present study, we discovered that miR-30d inhibitor notably reduced CPB2 toxin revulsive release of pro-inflammatory cytokines (IL-1β, IL-6, and IL-8), LDH activity, and the ROS level compared with the inhibitor NC group, and alleviated the inflammatory damage in the IPEC-J2 cells. By contrast, the miR-30d mimic aggravated CPB2-induced inflammatory response.

Studies have indicated that *PSME3* is involved in the bacterial infection process. The hematopoietic *PSME3*-deficient mice were more susceptible to bacterial infection, and increased bacterial burden in tissues (54). The *PSME3*-deficient mice lessened numbers of CD8+ T cells and showed reduced clearance of fungal infections in the lungs (37). In this study, we predicted the existence of a binding region between *PSME3* 3'UTR and miR-30d through the bioinformatics website. Meanwhile, we found that *PSME3* expression was observably higher in the CPB2 toxin revulsive IPEC-J2 cells, which was correlated negatively with miR-30d expression. The dual luciferase reporter assay manifested that the luciferase activity was lowered after the interaction of miR-30d with *PSME3* 3'UTR. Moreover, the miR-30d mimic decreased the expression of PSME3 at mRNA and protein levels, while the miR-30d inhibitor enhanced the expression of PSME3. Taken together, miR-30d targeting *PSME3* exacerbates CPB2 toxin-induced inflammatory response. To further determine the impact of *PSME3* on the CPB2 toxin-disposed IPEC-J2 cells, we constructed a PSME3 overexpression vector and designed PSME3 siRNA to transfect into the CPB2 toxin revulsive IPEC-J2 cells. Our results displayed that overexpression of *PSME3* signally alleviated the CPB2 toxin revulsive inflammatory damage in the IPEC-J2 cells, and the knockdown of *PSME3* increased the inflammatory injury. Furthermore, rescue experiments showed that *PSME3* reverses the effects of miR-30d on CPB2 toxin-induced inflammatory damage in the IEC-J2 cells.

Progression of the cell cycle is regulated by cyclins and cyclin-dependent kinases (CDKs) (55). Several studies have shown that the inflammatory response is associated with the cell cycle and proliferation. Melo-Salas et al. (56) showed that systemic inflammation altered the cell cycle progression of Hippocampal Type 2 Intermediate Precursor Cells and inhibited cell proliferation. In addition, studies have also indicated that cell cycle-related proteins contribute to the inhibition of inflammation (57). Xie et al. (58) demonstrated that cell cycle kinase attenuates the inflammatory response in Staphylococcus aureus-induced pneumonia by inhibiting the NF-kB signaling pathway. CDK4 is one of the important members of the cell cycle regulatory protein family and plays key roles in regulating the G1 phase of the cell cycle (59). The PCNA protein is a marker of cell proliferation, a vital factor of DNA replication and repair, and can be used to evaluate the growth of cell population (60, 61). p21 is a cyclin-dependent kinase inhibitor, which plays a momentous role in regulating the cell cycle progression. p21 acts as a cell cycle inhibitor and has anti-proliferative effects on normal cells (62, 63). Yu et al. (64) found that miR-30d refrained the proliferation and the cell cycle of renal cancer cells. miR-30d-5p also restrained the proliferation of non-small cell lung cancer cells by downregulating *CCNE2*, and induced G1/S cell cycle arrest (65). In this study, the miR-30d mimic enhanced the inhibition of CPB2 toxin on IPEC-J2 cell proliferation, blocked cell cycle progression, and inhibited the expression of cycle-related genes (*CDK4* and *PCNA*) and promoted the *p21* level, whereas the miR-30d inhibitor displayed the opposite effects. *PSME3* is involved in regulating the degradation of many key proteins and facilitates cell growth by mediating the degradation of cell cycle inhibitor p21 (66). Sanchez et al. (67) demonstrated that miR-7 targeted *PSME3* to trigger cell cycle arrest at the G1/S transition cell cycle. In this research, we manifested that overexpression of *PSME3* facilitated cell proliferation, cell cycle progression, and the expression of cycle-related genes in the CPB2 toxin-treated IPEC-J2 cells, whereas the knockdown of *PSME3* suppressed cell proliferation, cycle progression, and the expression of cycle-related genes.

## Conclusion

In conclusion, our research revealed that miR-30d aggravates CPB2 toxin-induced inflammation in the IPEC-J2 cells *via* targeting *PSME3* 3'UTR, and attenuates cell proliferation. Our studies suggest that miR-30d/PSME3 might be a novel target for the prevention and treatment of diarrhea in piglets, and establishes the foundation for further research in the breeding of pigs that are resistant to *C. perfringens*-induced diarrhea.

## Data Availability Statement

The original contributions presented in the study are included in the article/Supplementary Material; further inquiries can be directed to the corresponding authors.

## Ethics Statement

All animal experiments followed the approval of the Ethical Committee of Experimental Animal Center of Gansu Agricultural University (Approval No. 2006-398).

## Author Contributions

KX: conceptualization, software, formal analysis, writing—original draft preparation, and visualization. XG, PW, and KX: methodology. JZ, JY, and JL: validation. KX and XG: investigation. KX, XG, and JL: resources. KX and ZY: data curation. QY, ZY, and XH: writing—review and editing. SG: supervision. SG and ZY: project administration. SG: funding acquisition. All the authors have read and agreed to the published version of the manuscript.

## Funding

This research was funded by the Education Science and Technology Innovation Project of Gansu Province (GSSYLXM-02), the National Natural Science Foundation of China (31960646), and the Chief Special Project for the Pig and Chicken Industry of Gansu Province Modern Agricultural Industrial Technology System (GARS-ZJ-1).

## Conflict of Interest

The authors declare that the research was conducted in the absence of any commercial or financial relationships that could be construed as a potential conflict of interest.

## Publisher's Note

All claims expressed in this article are solely those of the authors and do not necessarily represent those of their affiliated organizations, or those of the publisher, the editors and the reviewers. Any product that may be evaluated in this article, or claim that may be made by its manufacturer, is not guaranteed or endorsed by the publisher.

## References

[1] Yue S Li Z Hu F Picimbon JF. Curing piglets from diarrhea and preparation of a healthymicrobiome with *Bacillus* treatment for industrial animal breeding. Sci Rep. (2020) 10:19476. 10.1038/s41598-020-75207-1 33173074 PMC7656456

[2] Yan Z Cai L Huang X Sun W Li S Wang P. Histological and comparative transcriptome analyses provide insights into small intestine health in diarrheal piglets after infection with *Clostridium Perfringens* Type C. Animals. (2019) 9:269. 10.3390/ani9050269 31126046 PMC6562977

[3] Wu Z Qin W Wu S Zhu G Bao W Wu S. Identification of microRNAs regulating *Escherichia coli* F18 infection in Meishan weaned piglets. Biol Direct. (2019) 11:59. 10.1186/s13062-016-0160-3 27809935 PMC5093996

[4] Uthe JJ Royaee A Lunney JK Stabel TJ Zhao SH Tuggle CK. Porcine differential gene expression in response to *Salmonellaenterica* serovars Choleraesuis and Typhimurium. Mol Immunol. (2007) 44:2900–14. 10.1016/j.molimm.2007.01.016 17337057

[5] Rubin JE Costa MO Hill JE Kittrell HE Champika F Huang Y. Reproduction of mucohaemorrhagic diarrhea and colitis indistinguishable from swine dysentery following experimental inoculation with “brachyspira hampsonii” strain 30446. PloS ONE. (2013) 8:e57146. 10.1371/journal.pone.0057146 23460829 PMC3584117

[6] Daneshmand A Kermanshahi H Mohammed J Sekhavati MH Javadmanesh A Ahmadian M. Intestinal changes and immune responses during *Clostridium perfringens*-induced necrotic enteritis in broiler chickens. Poult Sci. (2022) 101:101652. 10.1016/j.psj.2021.101652 35038649 PMC8762468

[7] Xiu L Zhu C Zhong Z Liu L Chen S Xu W. Prevalence and multilocus sequence typing of *Clostridium perfringens* isolated from different stages of a duck production chain. Food Microbiol. (2022) 102:103901. 10.1016/j.fm.2021.103901 34809933

[8] Rood JI Adams V Lacey J Lyras D McClane BA Melville SB. Expansion of the*Clostridium perfringens* toxin-based typing scheme. Anaerobe. (2018) 53:5–10. 10.1016/j.anaerobe.2018.04.011 29866424 PMC6195859

[9] Petit L Gibert M Popoff MR. *Clostridium perfringens*: toxinotype and genotype. Trends Microbiol. (1999) 7:104–10. 10.1016/S0966-842X(98)01430-9 10203838

[10] Waters M Savoie A Garmory HS Bueschel D Popoff MR Songer JG. Genotyping and phenotyping of beta2-toxigenic *Clostridium perfringens* fecal isolates associated with gastrointestinal diseases in piglets. J Clin Microbiol. (2003) 41:3584–91. 10.1128/JCM.41.8.3584-3591.2003 12904359 PMC179868

[11] Gibert M Jolivet-Renaud C Popoff MR. Beta2 toxin, a novel toxin produced by *Clostridium perfringens. Gene*. (1997) 203:65–73. 10.1016/S0378-1119(97)00493-9 9426008

[12] Dray T. *Clostridium perfringens* type A and beta2 toxin associated with enterotoxemia in a 5-week-old goat. Can Vet J. (2004) 45:251–3. 10.1111/j.1751-0813.2004.tb12652.x 15072200 PMC548614

[13] Klaasen H Molkenboer M Bakker J Miserez R Häni H Frey J. Detection of the β2 toxin gene of *Clostridium perfringens* in diarrhoeic piglets in the netherlands and switzerland. Pathog Dis. (2013) 24:325–32. 10.1016/S0928-8244(99)00049-8 10397318

[14] Zeng J Song F Yang Y Ma C Deng G Li Y. The generation and characterization of recombinant protein and antibodies of *Clostridium perfringens* beta2 toxin. J Immunol Res. (2016) 2016:5708468. 10.1155/2016/5708468 27672668 PMC5031884

[15] Gao X Yang Q Huang X Yan Z Zhang S Luo R. Effects of *Clostridium perfringens* beta2 toxin on apoptosis, inflammation, and barrier function of intestinal porcine epithelial cells. Microb Pathog. (2020) 147:104379. 10.1016/j.micpath.2020.104379 32649964

[16] Luo R Yang Q Huang X Yan Z Gao X Wang W. *Clostridium perfringens* beta2 toxin induced in vitro oxidative damage and its toxic assessment in porcine small intestinal epithelial cell lines. Gene. (2020) 759:144999. 10.1016/j.gene.2020.144999 32717305

[17] Lim LP Lau NC Weinstein EG Abdelhakim A Yekta S Rhoades MW. The microRNAs of Caenorhabditis elegans. Genes Dev. (2003) 17:991–1008. 10.1101/gad.1074403 12672692 PMC196042

[18] Dubey S Garg R. miRNAs: in the domain of cancer chemoresistance and stem cells. Acta Sci Cancer Biol. (2020) 4:01–03. 10.31080/ASCB.2020.04.020425887381

[19] Ye D Guo S Al-Sadi R Ma TY. MicroRNA regulation of intestinal epithelial tight junction permeability. Gastroenterology. (2011) 141:1323–33. 10.1053/j.gastro.2011.07.005 21763238 PMC3724217

[20] McKenna LB Schug J Vourekas A McKenna JB Bramswig NC Friedman JR. MicroRNAs control intestinal epithelial differentiation, architecture, and barrier function. Gastroenterology. (2010) 139:1654–64. 10.1053/j.gastro.2010.07.040 20659473 PMC3156097

[21] Biton M Levin A Slyper M Alkalay I Horwitz E Mor H. Epithelial microRNAs regulate gut mucosal immunity via epithelium -T cell crosstalk. Nat Immunol. (2011) 12:239–46. 10.1038/ni.1994 21278735

[22] Wang P Huang X Yan Z Yang Q Sun W Gao X. Analyses of miRNA in the ileum of diarrheic piglets caused by *Clostridium perfringens* type C. Microb Pathog. (2019) 136:103699. 10.1016/j.micpath.2019.103699 31472261

[23] Sun L Wu S Dai CH Sun SY Zhu GQ Wu SL. Insight into the molecular mechanism of miR-192 regulating *Escherichia coli* resistance in piglets. Biosci Rep. (2018) 38:BSR20171160. 10.1042/BSR20171160 29363554 PMC5821941

[24] Herrera-Uribe J Zaldivar-Lopez S Aguilar C Luque C Bautista R Carvajal A. Regulatory role of microRNA in mesenteric lymph nodes after *SalmonellaTyphimurium* infection. Vet Res. (2018) 49:9. 10.1186/s13567-018-0506-1 29391047 PMC5796392

[25] Lagos-Quintana M Rauhut R Yalcin A Meyer J Lendeckel W Tuschl T. Identification of tissue-specific MicroRNAs from mouse. Curr Biol. (2002) 12:735–9. 10.1016/S0960-9822(02)00809-6 12007417

[26] Liang L Yang Z Deng Q Jiang Y Cheng Y Sun Y. miR-30d-5p suppresses proliferation and autophagy by targeting *ATG5* in renal cell carcinoma. FEBS Open Bio. (2021) 11:529–40. 10.1002/2211-5463.13025 33145996 PMC7876493

[27] Zong S Zhao J Liu L. miR-30d induced apoptosis by targeting *Sox4* to inhibit the proliferation, invasion and migration of nephroblastoma. Onco Targets Ther. (2020) 13:7177–88. 10.2147/OTT.S251714 32821117 PMC7419636

[28] Karbiener M Neuhold C Opriessnig P Prokesch A Bogner-Strauss JG Scheideler M. MicroRNA-30c promotes human adipocyte differentiation and co-represses PAI-1 and ALK2. RNA Biol. (2011) 8:850–60. 10.4161/rna.8.5.16153 21878751

[29] Li W Hou G Lv J Lin F Song G Li R. MicroRNA-30d-5p ameliorates lipopolysaccharide-induced acute lung injury via activating AMPKalpha. Immunopharmacol Immunotoxicol. (2021) 43:431–42. 10.1080/08923973.2021.1933517 34157933

[30] Liu BY Li L Bai LW Xu CS. Long non-coding RNA XIST attenuates diabetic peripheral neuropathy by inducing autophagy through MicroRNA-30d-5p/sirtuin1 axis. Front Mol Biosci. (2021) 8:655157. 10.3389/fmolb.2021.655157 33996907 PMC8113765

[31] Zhao F Qu Y Zhu J Zhang L Huang L Liu H. miR-30d-5p Plays an important role in autophagy and apoptosis in developing rat brains after hypoxic-ischemic injury. J Neuropathol Exp Neurol. (2017) 76:709–19. 10.1093/jnen/nlx052 28789480

[32] Wang C Zhang Y Luo J Ding H Liu S Amer S. Identification of miRNomes reveals ssc-miR-30d-R_1 as a potential therapeutic target for PRRS viral infection. Sci Rep. (2016) 6:24854. 10.1038/srep24854 27117627 PMC4846818

[33] Nikaido T Shimada K Shibata M Hata M Sakamoto M Takasaki Y. Cloning and nucleotide sequence of cDNA for Ki antigen, a highly conserved nuclear protein detected with sera from patients with systemic lupus erythematosus. Clin Exp Immunol. (2010) 79:209–14. 10.1111/j.1365-2249.1990.tb05180.x 1968796 PMC1534747

[34] Song W Guo C Chen J Duan S Hu Y Zou Y. Silencing *PSME3* induces colorectal cancer radiosensitivity by down-regulating the expression of cyclin B1 and CKD1. Exp Biol Med. (2019) 244:1409–18. 10.1177/1535370219883408 31630568 PMC6900698

[35] Tanaka K. The proteasome: overview of structure and functions. Proc Jpn Acad Ser B Phys Biol Sci. (2009) 85:12–36. 10.2183/pjab.85.12 19145068 PMC3524306

[36] Moncsek A Gruner M Meyer H Lehmann A Kloetzel PM Stohwasser R. Evidence for anti-apoptotic roles of proteasome activator 28gamma via inhibiting caspase activity. Apoptosis. (2015) 20:1211–28. 10.1007/s10495-015-1149-6 26201457

[37] Barton LF Runnels HA Schell TD Cho Y Gibbons R Tevethia SS. Immune defects in 28-kDa proteasome activator gamma-deficient mice. J Immunol. (2004) 172:3948–54. 10.4049/jimmunol.172.6.3948 15004203

[38] Moriishi K Okabayashi T Nakai K Moriya K Koike K Murata S. Proteasome activator PA28gamma-dependent nuclear retention and degradation of hepatitis C virus core protein. J Virol. (2003) 77:10237–49. 10.1128/JVI.77.19.10237-10249.2003 12970408 PMC228494

[39] Huang X Sun W Yan Z Shi H Yang Q Wang P. Integrative analyses of long non-coding RNA and mRNA involved in piglet ileum immune response to *Clostridium perfringens* Type C Infec-tion. Front Cell Infect Microbiol. (2019) 9:130. 10.3389/fcimb.2019.00130 31114763 PMC6503642

[40] Livak KJ Schmittgen TDL. Analysis of relative gene expression data using real-time quantitative pcr and the 2(-Delta Delta C(T)) method. Methods. (2001) 25:402–8. 10.1006/meth.2001.1262 11846609

[41] Sun W Liu R Li P Li Q Cui H Zheng M. Chicken gga-miR-1306-5p targets *Tollip* and plays an important role in host response against *Salmonellaenteritidis* infection. J Anim Sci Biotechnol. (2019) 10:59. 10.1186/s40104-019-0365-2 31338187 PMC6628503

[42] Fu Q Shi H Shi M Meng L Zhang H Ren Y. bta-miR-29b attenuates apoptosis by directly targeting caspase-7 and NAIF1 and suppresses bovine viral diarrhea virus replication in MDBK cells. Can J Microbiol. (2014) 60:455–60. 10.1139/cjm-2014-0277 24965127

[43] Zhang L Zhang L Pan Y Gao J Xu Y Li X. Down-regulation of miR-218 by porcine reproductive and respiratory syndrome virus facilitates viral replication via inhibition of type I interferon responses. J Biol Chem. (2021) 296:100683. 10.1016/j.jbc.2021.100683 33887325 PMC8131720

[44] Qi X Cao Y Wu S Wu Z Bao W. miR-129a-3p inhibits PEDV replication by targeting the EDA-Mediated NF-kappaB pathway in IPEC-J2 cells. Int J Mol Sci. (2021) 22:8133. 10.3390/ijms22158133 34360898 PMC8347983

[45] Gao X Yang Q Zhang S Huang X Yan Z Wang P. Epigenetic upregulation of ssc-miR-124a following treatment with *Clostridium perfringens* beta2-toxin attenuates both apoptosis and inflammation in intestinal porcine epithelial cells. Arch Biochem Biophys. (2021) 701:108806. 10.1016/j.abb.2021.108806 33587903

[46] Luo R Yan Z Yang Q Huang X Gao X Wang P. Inhibition of ssc-microRNA-140-5p ameliorates the Clostridium perfringens beta2 toxin-induced inflammatory response in IPEC-J2 cells via the ERK1/2 and JNK pathways by targeting VEGFA. Mol Immunol. (2020) 127:12–20. 10.1016/j.molimm.2020.08.017 32905904

[47] Kumar S Gupta P Khanal S Shahi A Kumar P Sarin SK. Overexpression of microRNA-30a inhibits hepatitis Bvirus X protein-induced autophagosome formation in hepatic cells. FEBS J. (2015) 282:1152–63. 10.1111/febs.13209 25620738

[48] Zhang X Daucher M Armistead D Russell R Kottilil S. MicroRNA expression profiling in HCV-infected human hepatoma cells identifies potential anti-viral targets induced by interferon-α. PloS ONE. (2013) 8:e55733. 10.1371/journal.pone.0055733 23418453 PMC3572124

[49] Lin ZY Chen G Zhang YQ He HC Liang YX Ye JH. MicroRNA-30d promotes angiogenesis and tumor growth via MYPT1/c-JUN/VEGFA pathway and predicts aggressive outcome in prostate cancer. Mol Cancer. (2017) 16:48. 10.1186/s12943-017-0615-x 28241827 PMC5327510

[50] Wu Y Zhang J Hou S Cheng Z Yuan M. Non-small cell lung cancer: miR-30d suppresses tumor invasion and migration by directly targeting *NFIB*. Biotechnol Lett. (2017) 39:1827–34. 10.1007/s10529-017-2428-9 28861760

[51] Zhang R Xu J Zhao J Bai J. MiR-30d suppresses cell proliferation of colon cancer cells by inhibiting cell autophagy and promoting cell apoptosis. Tumour Biol. (2017*)* 39:1010428317703984. 10.1177/1010428317703984 28651493

[52] Li H Li J Zhai Y Zhang L Cui P Feng L. Gga-miR-30d regulates infectious bronchitis virus infection by targeting *USP47* in HD11 cells. Microb Pathog. (2020) 141:103998. 10.1016/j.micpath.2020.103998 31982568 PMC7125550

[53] Zhang K Gu X Lan J Zhang Y Ahmed KP Liu Z. Selenium-deficient diet induces inflammatory response in the pig adrenal glands by activating TLR4/NF-κB pathway via miR-30d-R_1. Metallomics. (2021) 13:mfab037. 10.1093/mtomcs/mfab037 34132350

[54] Sun J Luan Y Xiang D Tan X Chen H Deng Q. The 11S Proteasome subunit PSME3 is a positive feedforward regulator of NF-kappaB and important for host defense against bacterial pathogens. Cell Rep. (2016) 14:737–49. 10.1016/j.celrep.2015.12.069 26776519 PMC4740229

[55] Gao X Leone GW Wang H. Cyclin D-CDK4/6 functions in cancer. Adv Cancer Res. (2020) 148:147–69. 10.1016/bs.acr.2020.02.002 32723562

[56] Melo-Salas MS Perez-Dominguez M Zepeda A. Systemic inflammation impairs proliferation of hippocampal type 2 intermediate precursor cells. Cell Mol Neurobiol. (2018) 38:1517–28. 10.1007/s10571-018-0624-3 30315388 PMC11469845

[57] Syahirah R Hsu AY Deng Q. A curious case of cyclin-dependent kinases in neutrophils. J Leukoc Biol. (2022) 111:1057–68. 10.1002/JLB.2RU1021-573R 35188696 PMC9035055

[58] Xie F Chen R Zhao J Xu C Zan C Yue B. Cell cycle kinase CHEK2 in macrophages alleviates the inflammatory response to Staphylococcus aureus-induced pneumonia. Exp Lung Res. (2022) 1–8. 10.1080/01902148.2022.2029625 35075953

[59] Liu B Li X Sun F Tong X Bai Y Jin K. HP-CagA+ regulates the expression of CDK4/CyclinD1 via reg3 to change cell cycle and promote cell proliferation. Int J Mol Sci. (2019) 21:224. 10.3390/ijms21010224 31905669 PMC6981641

[60] Gonzalez-Magana A Blanco FJ. Human PCNA structure, function and interactions. Biomolecules. (2020) 10:570. 10.3390/biom10040570 32276417 PMC7225939

[61] Jurikova M Danihel L Polak S Varga I. Ki67, PCNA, and MCM proteins: markers of proliferation in the diagnosis of breast cancer. Acta Histochem. (2016) 118:544–52. 10.1016/j.acthis.2016.05.002 27246286

[62] Shamloo B Usluer S. p21 in cancer research. Cancers. (2019) 11:1178. 10.3390/cancers11081178 31416295 PMC6721478

[63] Wade Harper J Adami GR Wei N Keyomarsi K Elledge SJ. The p21 cdk-interacting protein cip1 is a potent inhibitor of G1 cyclin-dependent kinases-sciencedirect. Cell. (1993) 75:805–16. 10.1016/0092-8674(93)90499-G 8242751

[64] Yu H Lin X Wang F Zhang B Wang W Shi H. Proliferation inhibition and the underlying molecular mechanisms of microRNA-30d in renal carcinoma cells. Oncol Lett. (2014) 7:799–804. 10.3892/ol.2013.1754 24520297 PMC3919943

[65] Chen D Guo W Qiu Z Wang Q Li Y Liang L. MicroRNA-30d-5p inhibits tumour cell proliferation and motility by directly targeting CCNE2 in non-small cell lung cancer. Cancer Lett. (2015) 362:208–17. 10.1016/j.canlet.2015.03.041 25843294

[66] Li X Amazit L Long W Lonard DM Monaco JJ O'Malley BW. Ubiquitin-and ATP-independent proteolytic turnover of p21 by the REGgamma-proteasome pathway. Mol Cell. (2007) 26:831–42. 10.1016/j.molcel.2007.05.028 17588518

[67] Sanchez N Gallagher M Lao N Gallagher C Clarke C Doolan P. MiR-7 triggers cell cycle arrest at the G1/S transition by targeting multiple genes including Skp2 and Psme3. PloS ONE. (2013) 8:e65671. 10.1371/journal.pone.0065671 23762407 PMC3675065

