# Supplementary material for: miR-30d Inhibition Protects IPEC-J2 Cells Against Clostridium perfringens Beta2 Toxin-Induced Inflammatory Injury
Source: Front Vet Sci. 2022 Jun 21;9:909500. doi: 10.3389/fvets.2022.909500 (PMC9253665; doi:10.3389/fvets.2022.909500)
Supplement: Supplementary file 1 [file Table_1.DOCX]

Supplementary Material

Table S1. Sequences of miR-30d mimic, inhibitor, si-PSME3, and their NCs

| Name | Sense (5' to 3') | Antisense (5' to 3') |
| --- | --- | --- |
| miR-30d mimic | UGUAAACAUCCCCGACUGGAAGCU | CUUCCAGUCGGGGAUGUUUACAUU |
| mimic NC (si-NC) | UUCUCCGAACGUGUCACGUTT | ACGUGACACGUUCGGAGAATT |
| miR-30d inhibitor | AGCUUCCAGUCGGGGAUGUUUACA | / |
| inhibitor NC | CAGUACUUUUGUGUAGUACAA | / |
| si1-PSME3 | GCAGCUGGUGGACAUUAUUTT | AAUAAUGUCCACCAGCUGCTT |
| si2-PSME3 | GGAAGUAAAGCUCAAGGUUTT | AACCUUGAGCUUUACUUCCTT |
