# Supplementary material for: miR-30d Inhibition Protects IPEC-J2 Cells Against Clostridium perfringens Beta2 Toxin-Induced Inflammatory Injury
Source: Front Vet Sci. 2022 Jun 21;9:909500. doi: 10.3389/fvets.2022.909500 (PMC9253665; doi:10.3389/fvets.2022.909500)
Supplement: Supplementary file 2 [file Data_Sheet_1.zip › raw data/flow cytometry(cell cycle).docx]

Control


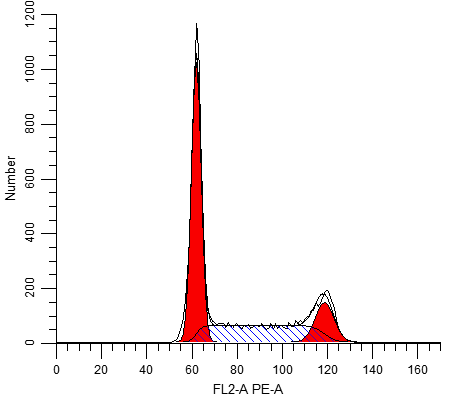


File analyzed: 7.fcs

Date analyzed: 4-Jan-2022

Model: 1nn0n_DSD

Analysis type: Manual analysis

Auto Linearity: No

Ploidy Mode: First cycle is diploid

Diploid: 100.00 %

Dip G1: 53.33 % at 61.86

Dip G2: 14.30 % at 118.77

Dip S: 32.36 % G2/G1: 1.92

%CV: 3.50

Total S-Phase: 32.36 %

Total B.A.D.: 0.00 % no debris no aggs

CPB2


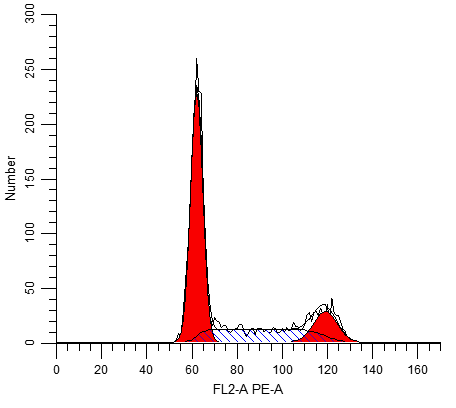


File analyzed: 12.fcs

Date analyzed: 4-Jan-2022

Model: 1nn0n_DSD

Analysis type: Manual analysis

Auto Linearity: No

Ploidy Mode: First cycle is diploid

Diploid: 100.00 %

Dip G1: 61.00 % at 62.05

Dip G2: 14.18 % at 119.13

Dip S: 24.82 % G2/G1: 1.92

%CV: 4.35

Total S-Phase: 24.82 %

Total B.A.D.: 0.00 % no debris no aggs

mimic NC+CPB2


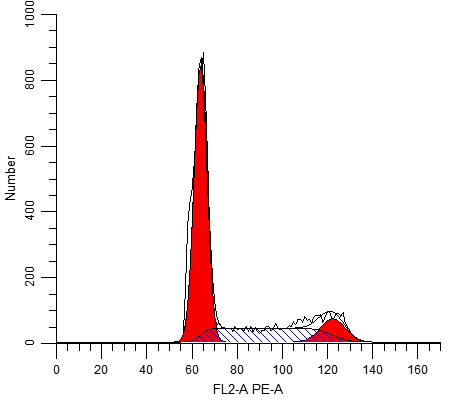


File analyzed: 1.fcs

Date analyzed: 4-Jan-2022

Model: 1nn0n_DSD

Analysis type: Manual analysis

Auto Linearity: No

Ploidy Mode: First cycle is diploid

Diploid: 100.00 %

Dip G1: 63.92 % at 63.77

Dip G2: 10.21 % at 122.44

Dip S: 25.87 % G2/G1: 1.92

%CV: 4.48

Total S-Phase: 25.87 %

Total B.A.D.: 0.00 % no debris no aggs

mimic+CPB2


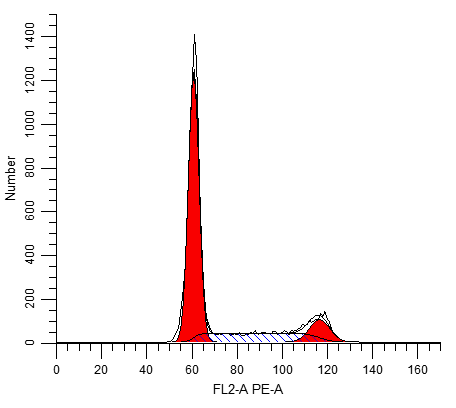


File analyzed: 10.fcs

Date analyzed: 4-Jan-2022

Model: 1nn0n_DSD

Analysis type: Manual analysis

Auto Linearity: No

Ploidy Mode: First cycle is diploid

Diploid: 100.00 %

Dip G1: 68.55 % at 60.70

Dip G2: 10.81 % at 116.55

Dip S: 20.64 % G2/G1: 1.92

%CV: 3.88

Total S-Phase: 20.64 %

Total B.A.D.: 0.00 % no debris no aggs

inhibitor NC+CPB2


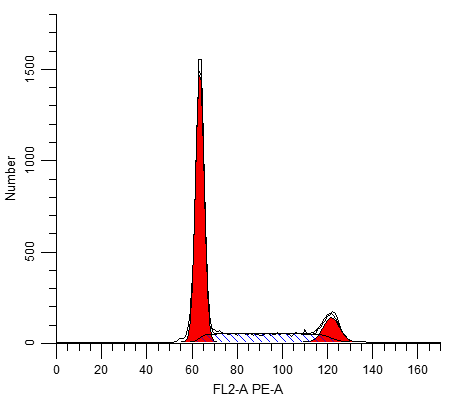


File analyzed: 4.fcs

Date analyzed: 4-Jan-2022

Model: 1nn0n_DSD

Analysis type: Manual analysis

Auto Linearity: No

Ploidy Mode: First cycle is diploid

Diploid: 100.00 %

Dip G1: 65.46 % at 63.42

Dip G2: 11.07 % at 121.76

Dip S: 23.47 % G2/G1: 1.92

%CV: 2.92

Total S-Phase: 23.47 %

Total B.A.D.: 0.00 % no debris no aggs

inhibitor+CPB2


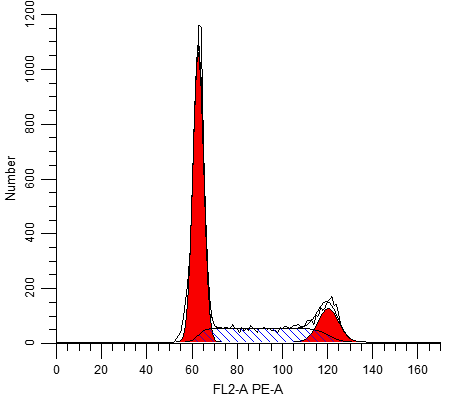


File analyzed: 5.fcs

Date analyzed: 4-Jan-2022

Model: 1nn0n_DSD

Analysis type: Manual analysis

Auto Linearity: No

Ploidy Mode: First cycle is diploid

Diploid: 100.00 %

Dip G1: 60.01 % at 62.73

Dip G2: 13.17 % at 120.44

Dip S: 26.81 % G2/G1: 1.92

%CV: 3.75

Total S-Phase: 26.81 %

Total B.A.D.: 0.00 % no debris no aggs

pcDNA3.1+CPB2


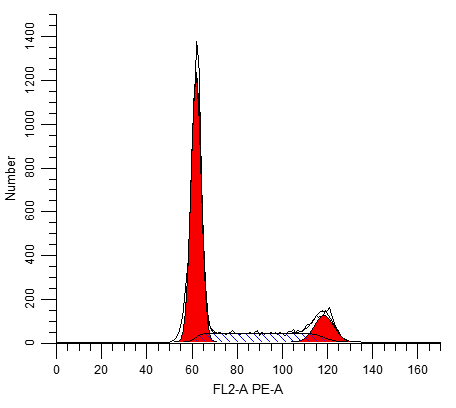


File analyzed: 6.fcs

Date analyzed: 4-Jan-2022

Model: 1nn0n_DSD

Analysis type: Manual analysis

Auto Linearity: No

Ploidy Mode: First cycle is diploid

Diploid: 100.00 %

Dip G1: 65.51 % at 61.76

Dip G2: 12.42 % at 118.57

Dip S: 22.08 % G2/G1: 1.92

%CV: 3.64

Total S-Phase: 22.08 %

Total B.A.D.: 0.00 % no debris no aggs

pc-PSME3+CPB2


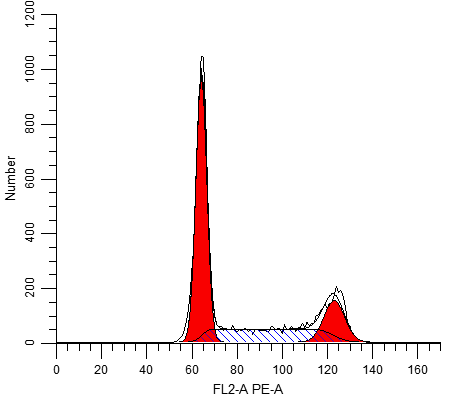


File analyzed: 11.fcs

Date analyzed: 4-Jan-2022

Model: 1nn0n_DSD

Analysis type: Manual analysis

Auto Linearity: No

Ploidy Mode: First cycle is diploid

Diploid: 100.00 %

Dip G1: 56.47 % at 64.10

Dip G2: 16.76 % at 123.06

Dip S: 26.77 % G2/G1: 1.92

%CV: 3.74

Total S-Phase: 26.77 %

Total B.A.D.: 0.00 % no debris no aggs

si-NC+CPB2


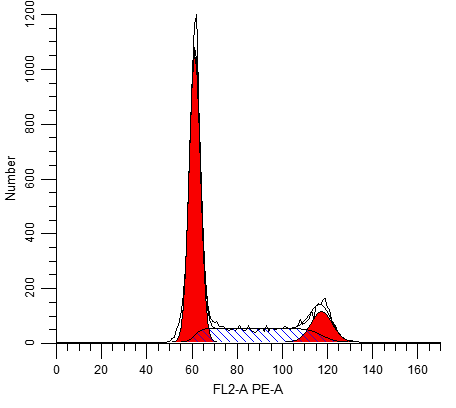


File analyzed: 8.fcs

Date analyzed: 4-Jan-2022

Model: 1nn0n_DSD

Analysis type: Manual analysis

Auto Linearity: No

Ploidy Mode: First cycle is diploid

Diploid: 100.00 %

Dip G1: 61.78 % at 61.18

Dip G2: 12.35 % at 117.47

Dip S: 25.87 % G2/G1: 1.92

%CV: 4.03

Total S-Phase: 25.87 %

Total B.A.D.: 0.00 % no debris no aggs

si-PSME3+CPB2


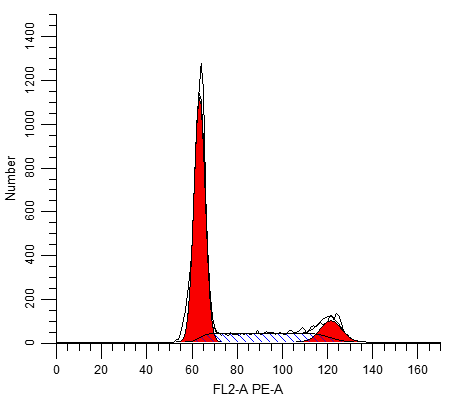


File analyzed: 2.fcs

Date analyzed: 4-Jan-2022

Model: 1nn0n_DSD

Analysis type: Manual analysis

Auto Linearity: No

Ploidy Mode: First cycle is diploid

Diploid: 100.00 %

Dip G1: 67.12 % at 63.31

Dip G2: 11.02 % at 121.55

Dip S: 21.86 % G2/G1: 1.92

%CV: 3.85

Total S-Phase: 21.86 %

Total B.A.D.: 0.00 % no debris no aggs

mimic+CPB2


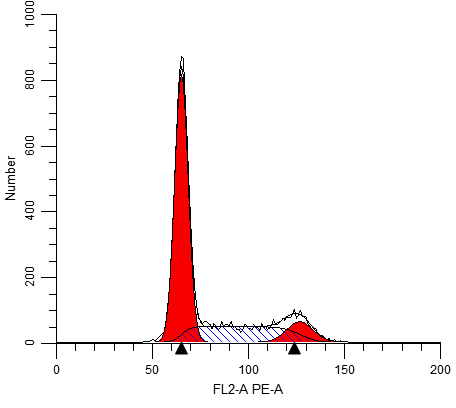


File analyzed: 9.fcs

Date analyzed: 21-Jun-2021

Model: 1nn0n_DSD

Analysis type: Manual analysis

Auto Linearity: No

Ploidy Mode: First cycle is diploid

Diploid: 100.00 %

Dip G1: 64.97 % at 65.01

Dip G2: 9.56 % at 126.76

Dip S: 25.47 % G2/G1: 1.95

%CV: 5.41

Total S-Phase: 25.47 %

Total B.A.D.: 0.00 % no debris no aggs

mimic+pcDNA3.1+CPB2


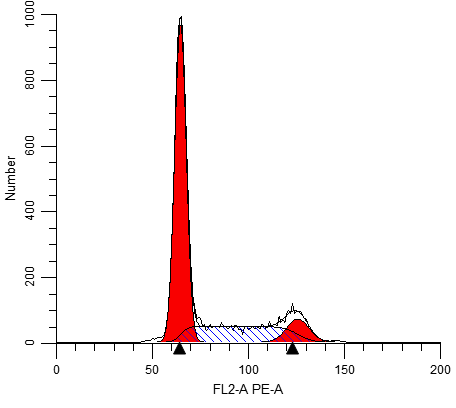


File analyzed: 3.fcs

Date analyzed: 21-Jun-2021

Model: 1nn0n_DSD

Analysis type: Manual analysis

Auto Linearity: No

Ploidy Mode: First cycle is diploid

Diploid: 100.00 %

Dip G1: 65.57 % at 64.50

Dip G2: 9.15 % at 125.78

Dip S: 25.27 % G2/G1: 1.95

%CV: 4.62

Total S-Phase: 25.27 %

Total B.A.D.: 0.00 % no debris no aggs

mimic+pc-PSME3+CPB2


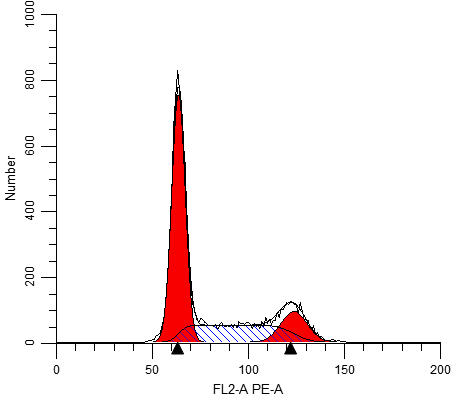


File analyzed: 2.fcs

Date analyzed: 21-Jun-2021

Model: 1nn0n_DSD

Analysis type: Manual analysis

Auto Linearity: No

Ploidy Mode: First cycle is diploid

Diploid: 100.00 %

Dip G1: 58.81 % at 63.46

Dip G2: 14.05 % at 123.74

Dip S: 27.14 % G2/G1: 1.95

%CV: 5.50

Total S-Phase: 27.14 %

Total B.A.D.: 0.00 % no debris no aggs
