# Supplementary material for: miR-30d Inhibition Protects IPEC-J2 Cells Against Clostridium perfringens Beta2 Toxin-Induced Inflammatory Injury
Source: Front Vet Sci. 2022 Jun 21;9:909500. doi: 10.3389/fvets.2022.909500 (PMC9253665; doi:10.3389/fvets.2022.909500)
Supplement: Supplementary file 2 [file Data_Sheet_1.zip › raw data/western blot.docx]

1 2 3 4


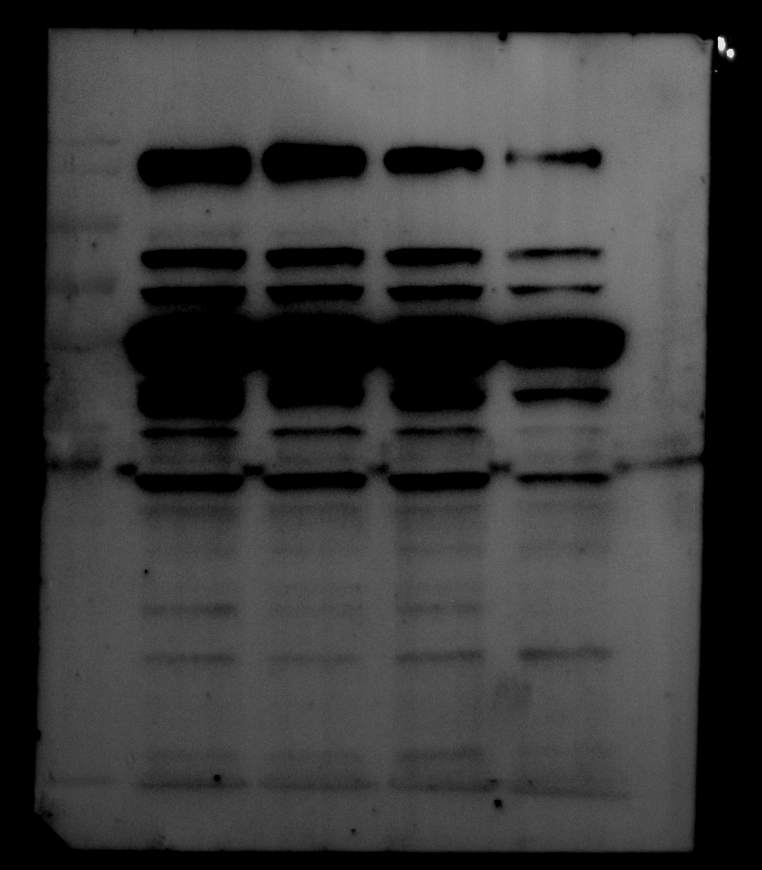


PSME3

1 2 3 4


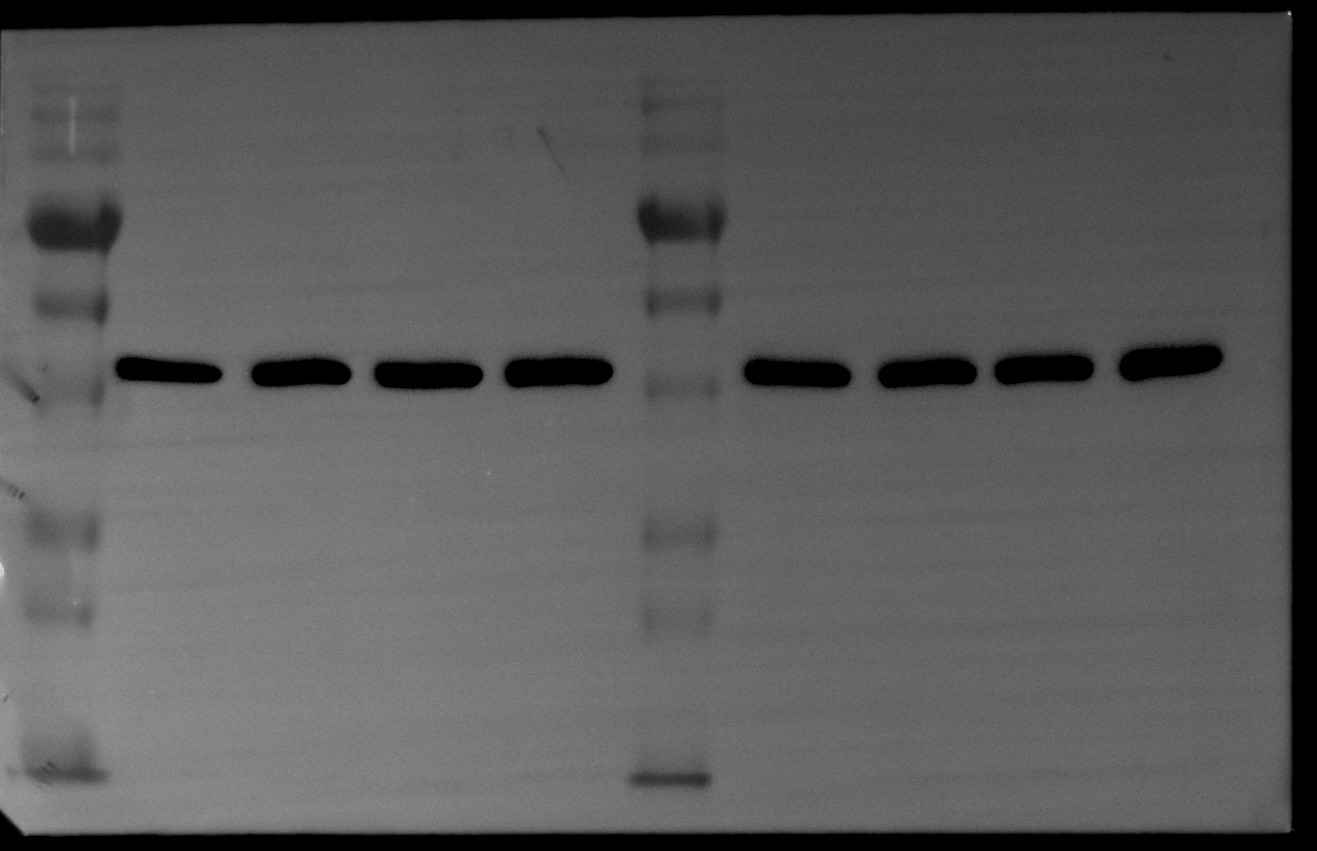


β-actin

Figure 4G. 1. mimic NC+CPB2; 2. mimic+CPB2; 3. inhibitor NC+CPB2; 4. inhibitor+CPB2


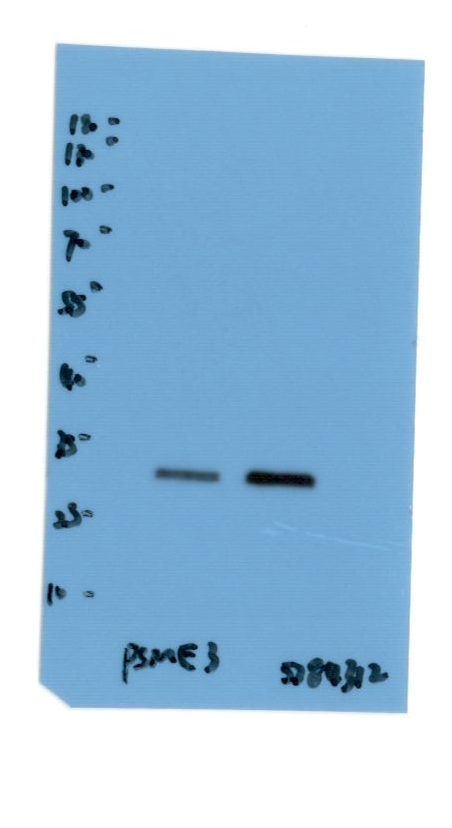

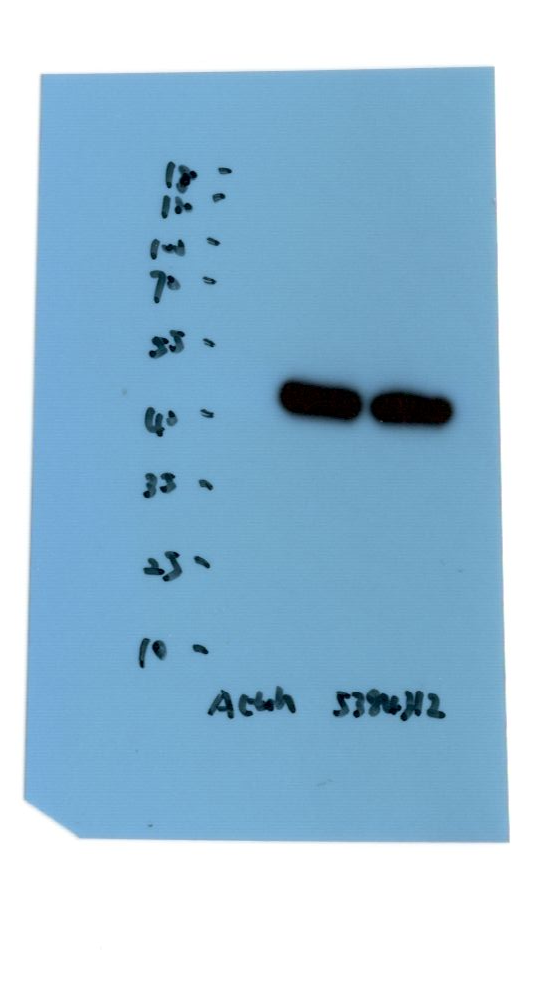


1 2

1 2

Figure 5C. 1. pcDNA3.1+CPB2; 2. pc-PSME3+CPB2

β-actin

PSME3

1 2

1 2


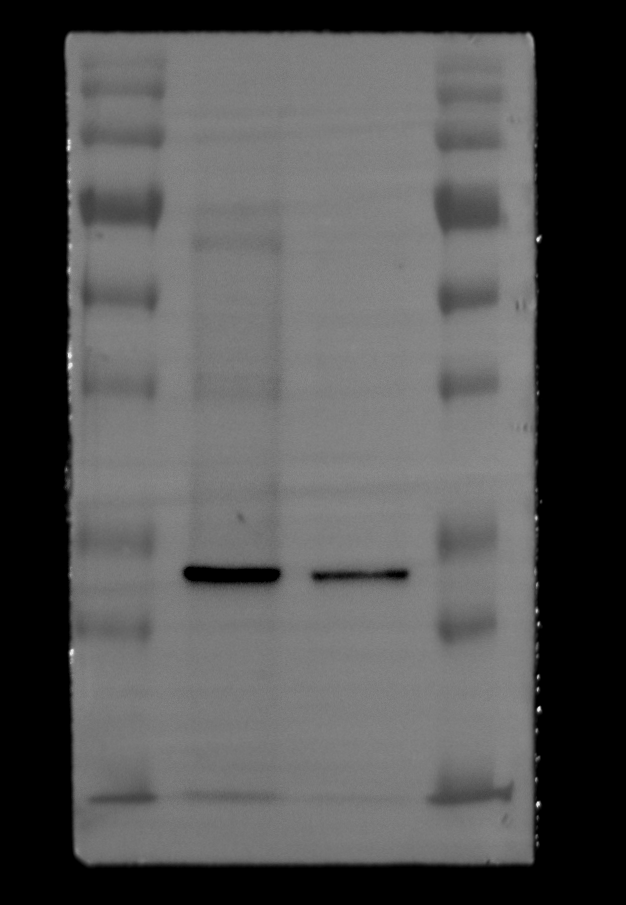

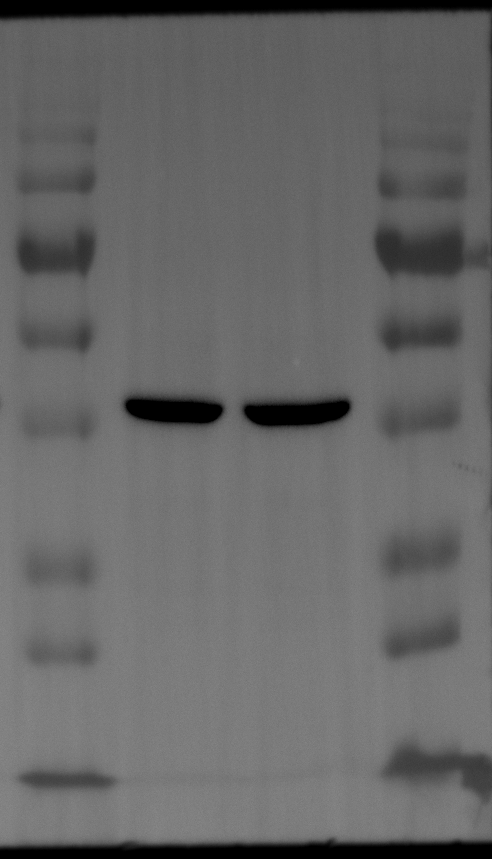


β-actin

PSME3

Figure 5D. 1. si-NC+CPB2; 2. si-PSME3+CPB2
