# Supplementary figures and images for: miR-30d Inhibition Protects IPEC-J2 Cells Against Clostridium perfringens Beta2 Toxin-Induced Inflammatory Injury
Source: Front Vet Sci. 2022 Jun 21;9:909500. doi: 10.3389/fvets.2022.909500 (PMC9253665; doi:10.3389/fvets.2022.909500)

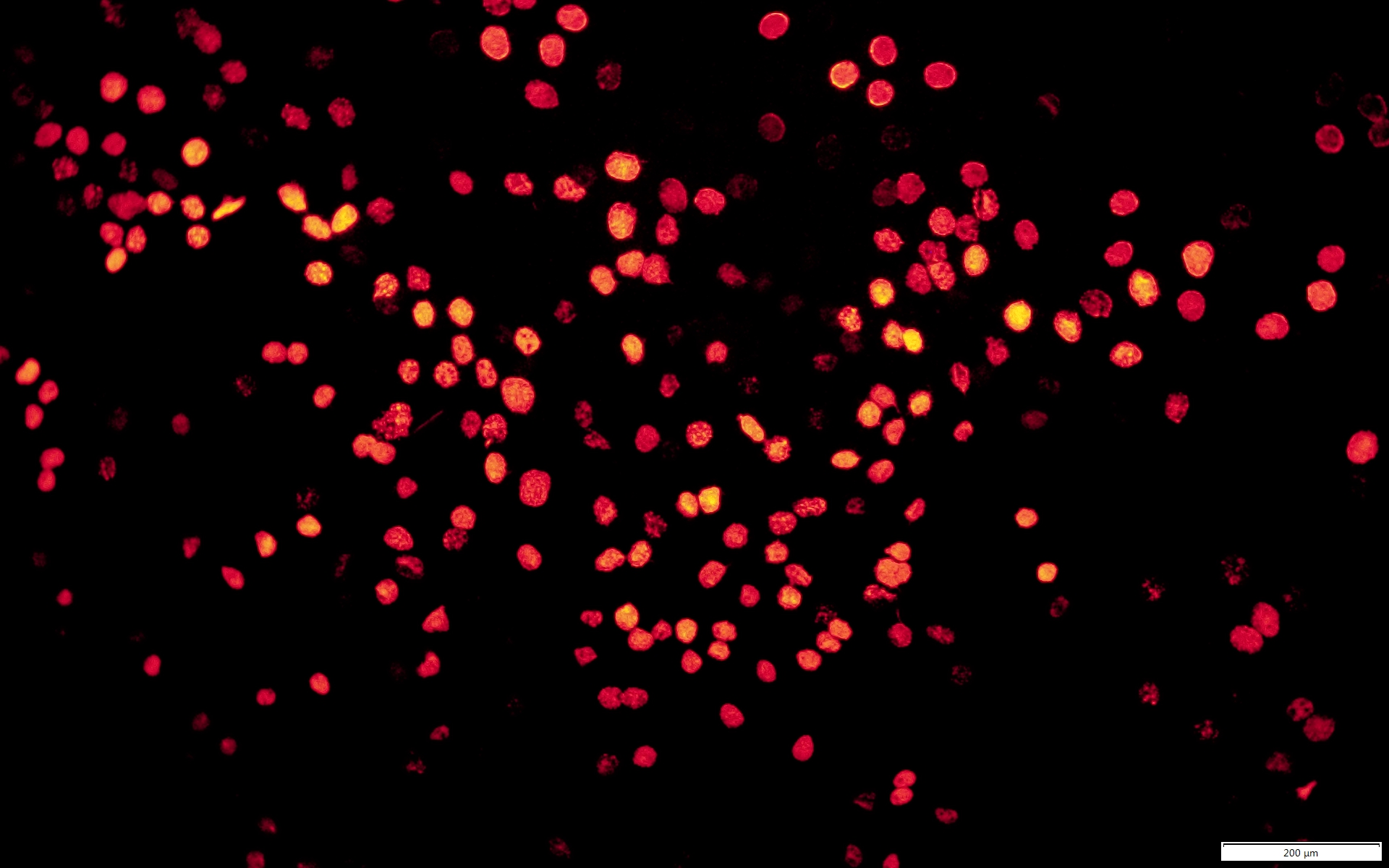

Supplement: Supplementary file 2 [file Data_Sheet_1.zip › raw data/EdU/Figure 3B/Control (EdU).jpg]

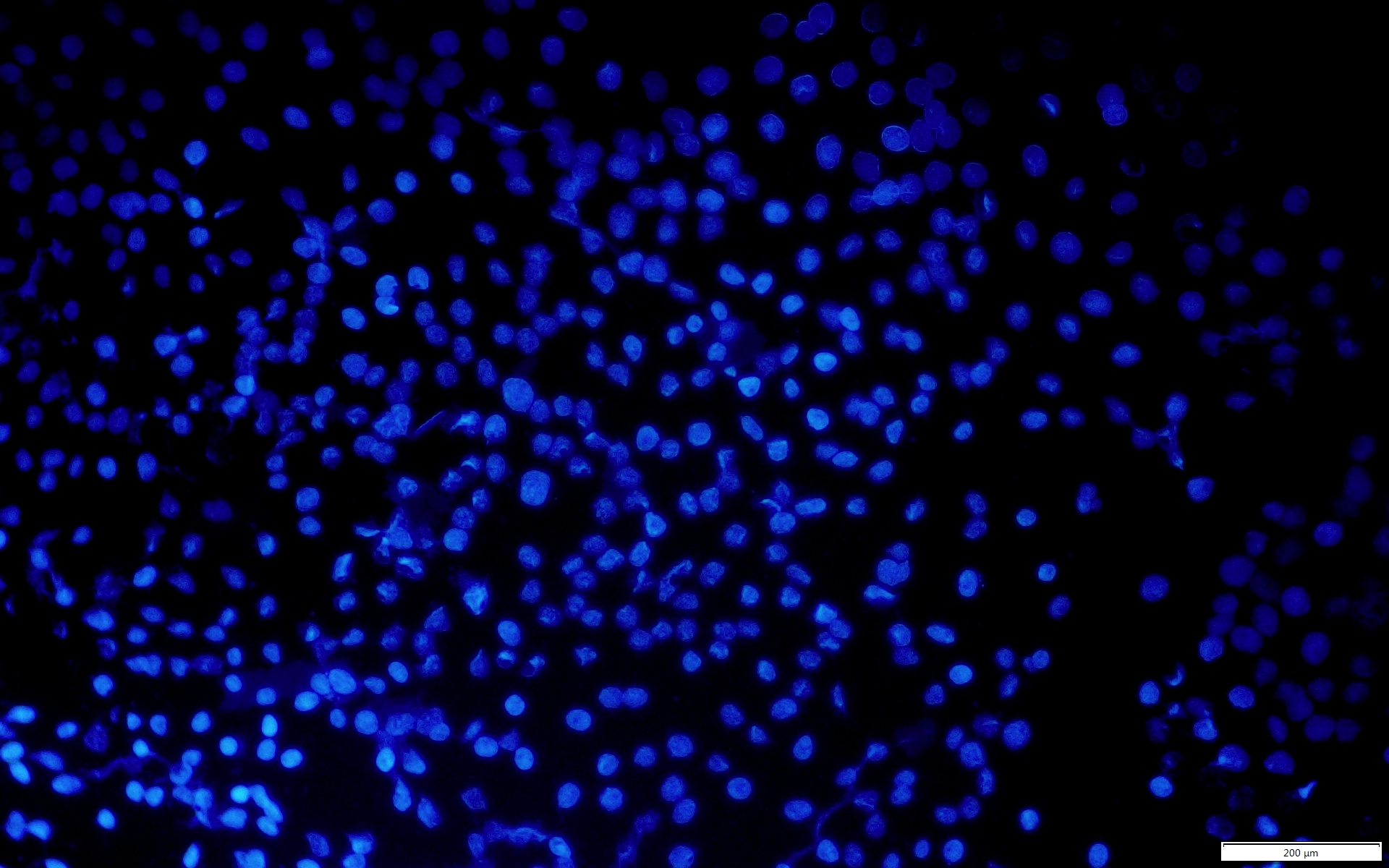

Supplement: Supplementary file 2 [file Data_Sheet_1.zip › raw data/EdU/Figure 3B/Control (Hoechst).jpg]

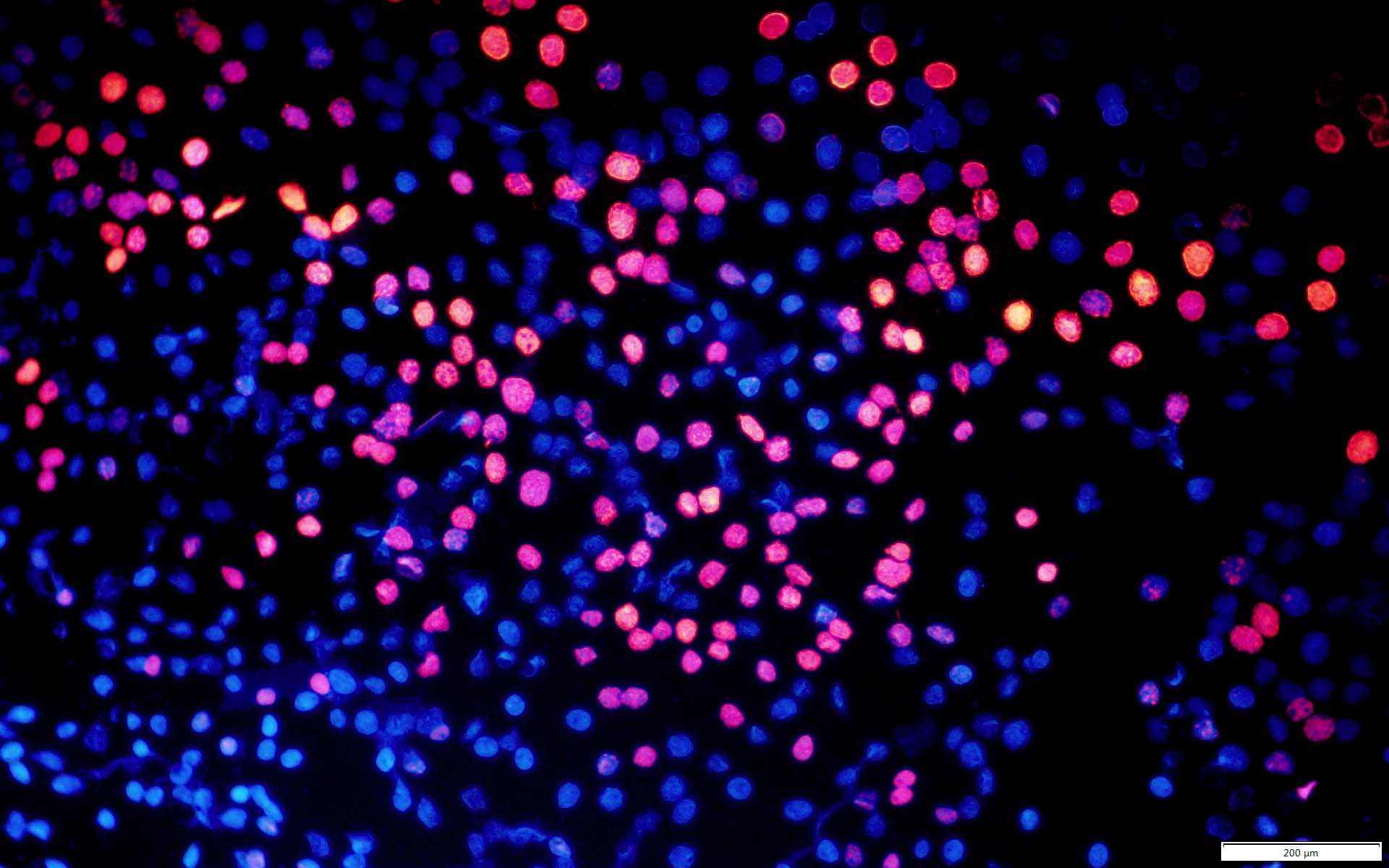

Supplement: Supplementary file 2 [file Data_Sheet_1.zip › raw data/EdU/Figure 3B/Control (Merge).jpg]

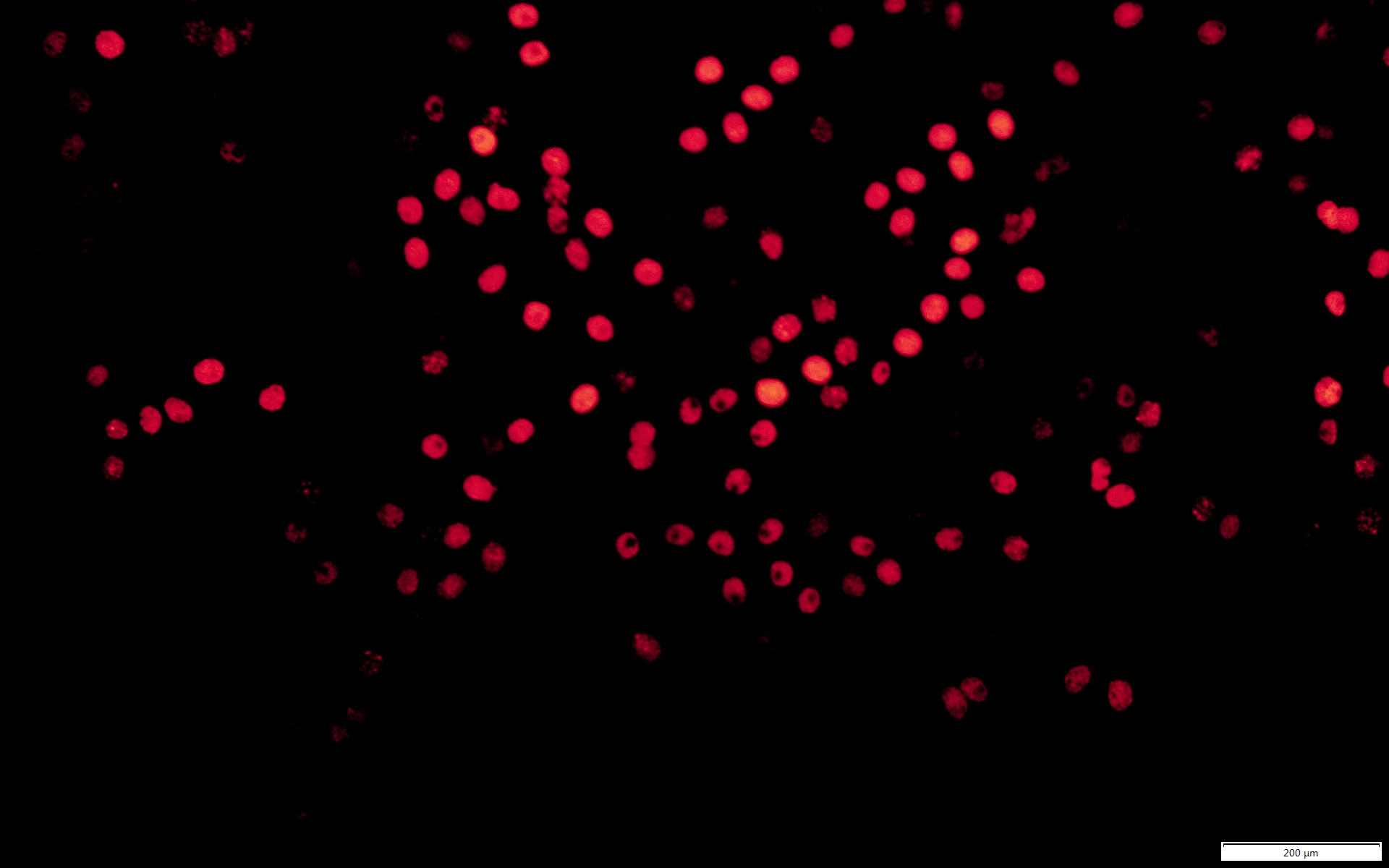

Supplement: Supplementary file 2 [file Data_Sheet_1.zip › raw data/EdU/Figure 3B/CPB2 (EdU).jpg]

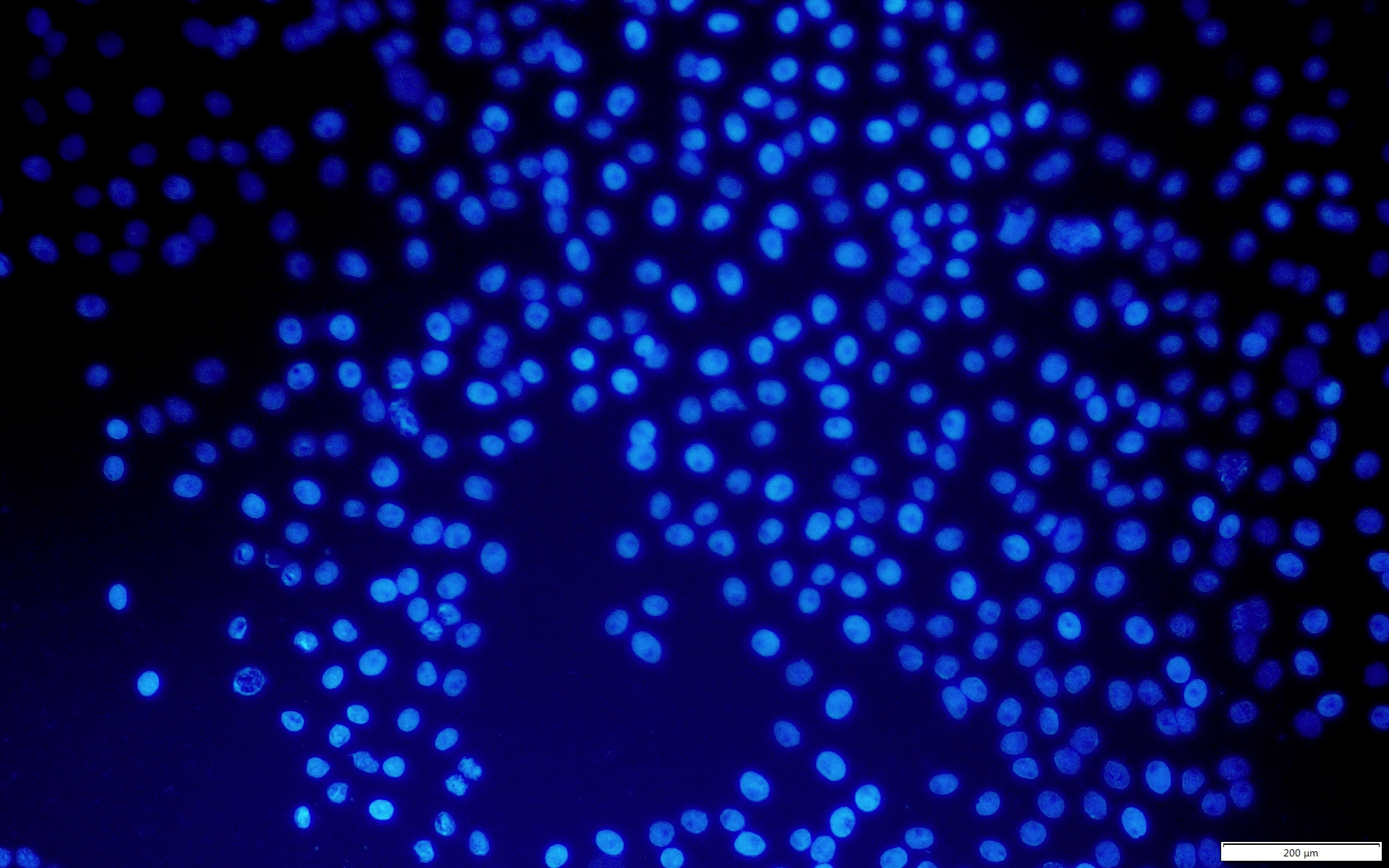

Supplement: Supplementary file 2 [file Data_Sheet_1.zip › raw data/EdU/Figure 3B/CPB2 (Hoechst).jpg]

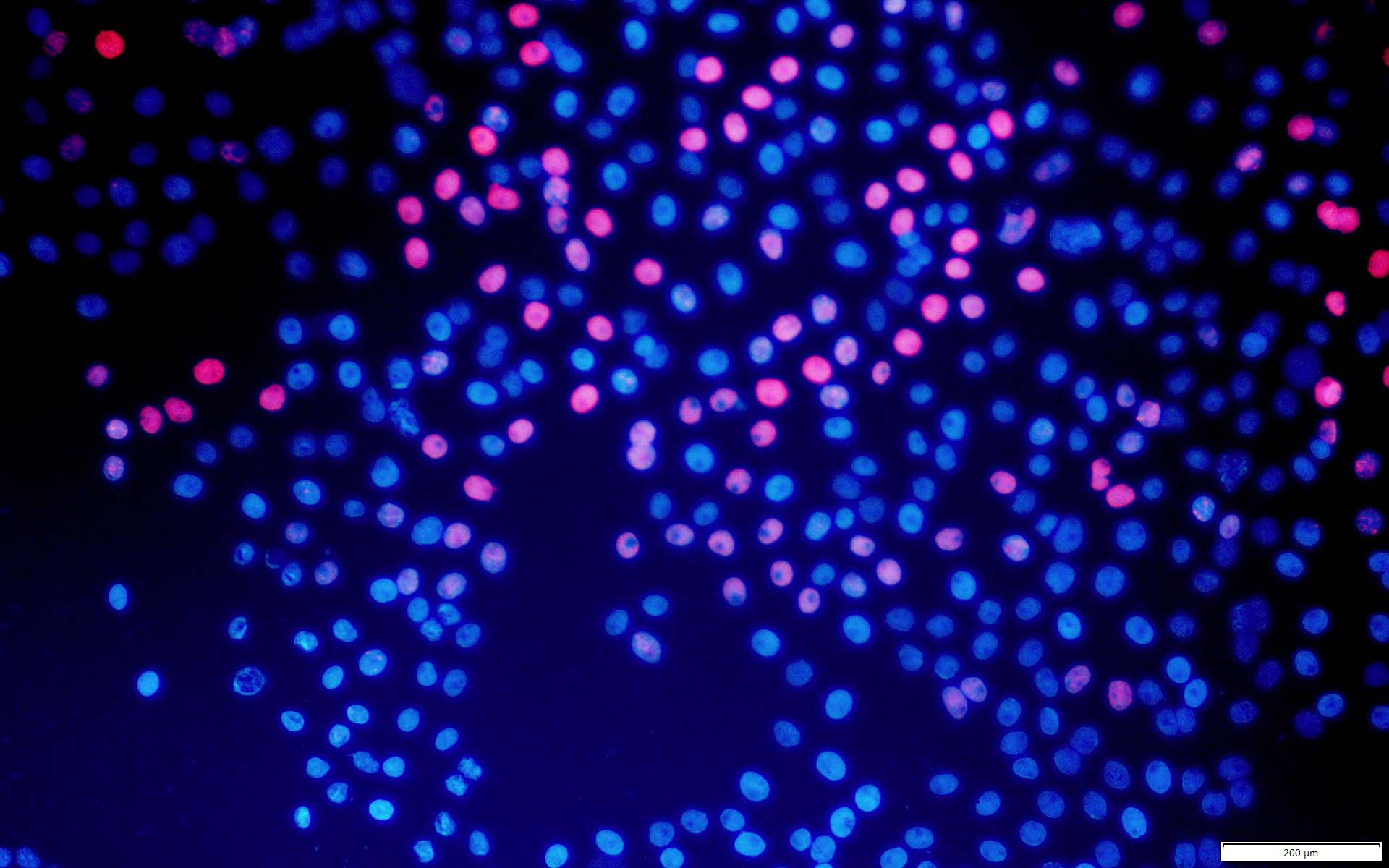

Supplement: Supplementary file 2 [file Data_Sheet_1.zip › raw data/EdU/Figure 3B/CPB2 (Merge).jpg]

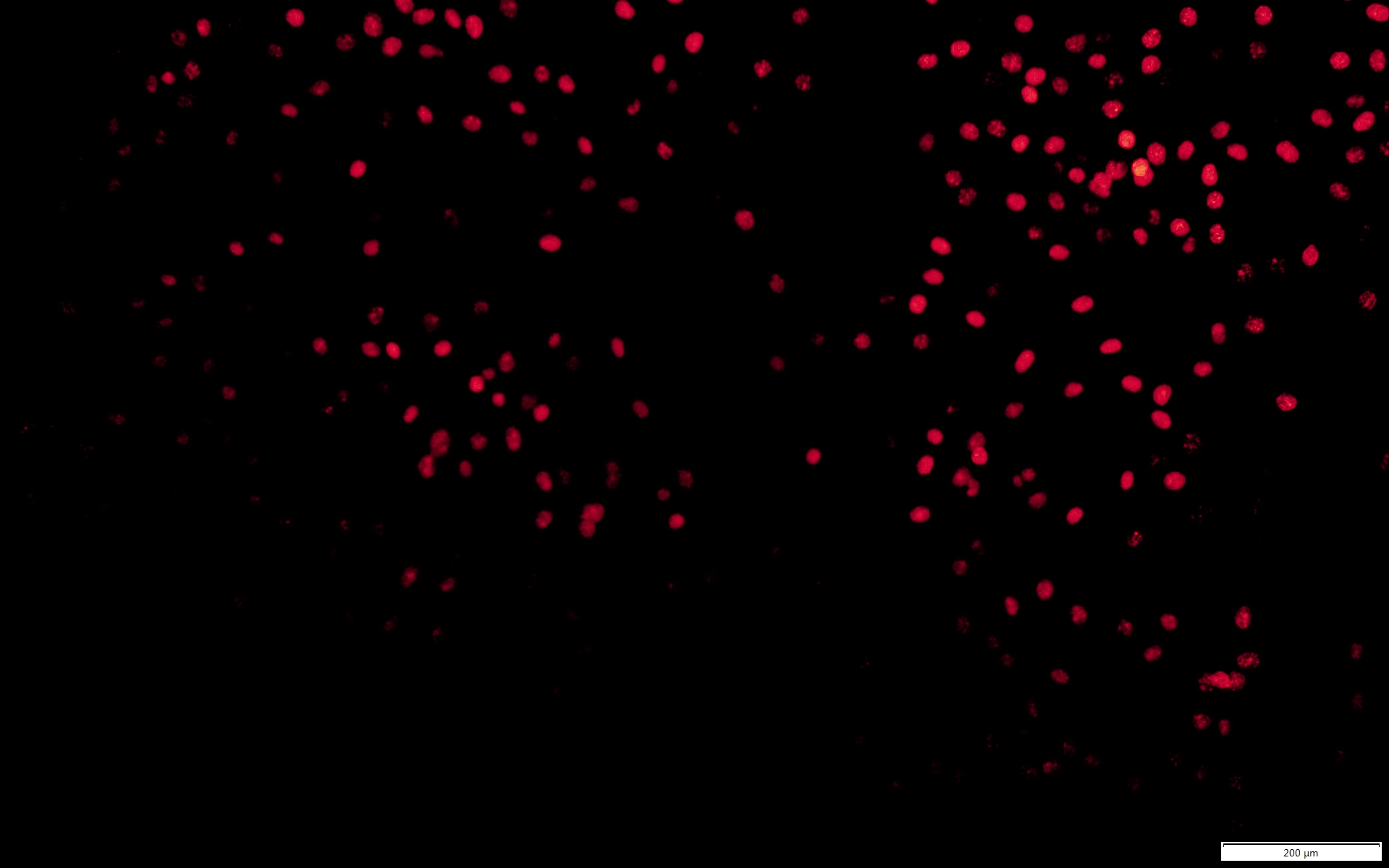

Supplement: Supplementary file 2 [file Data_Sheet_1.zip › raw data/EdU/Figure 3B/inhibitor NC+CPB2 (EdU).jpg]

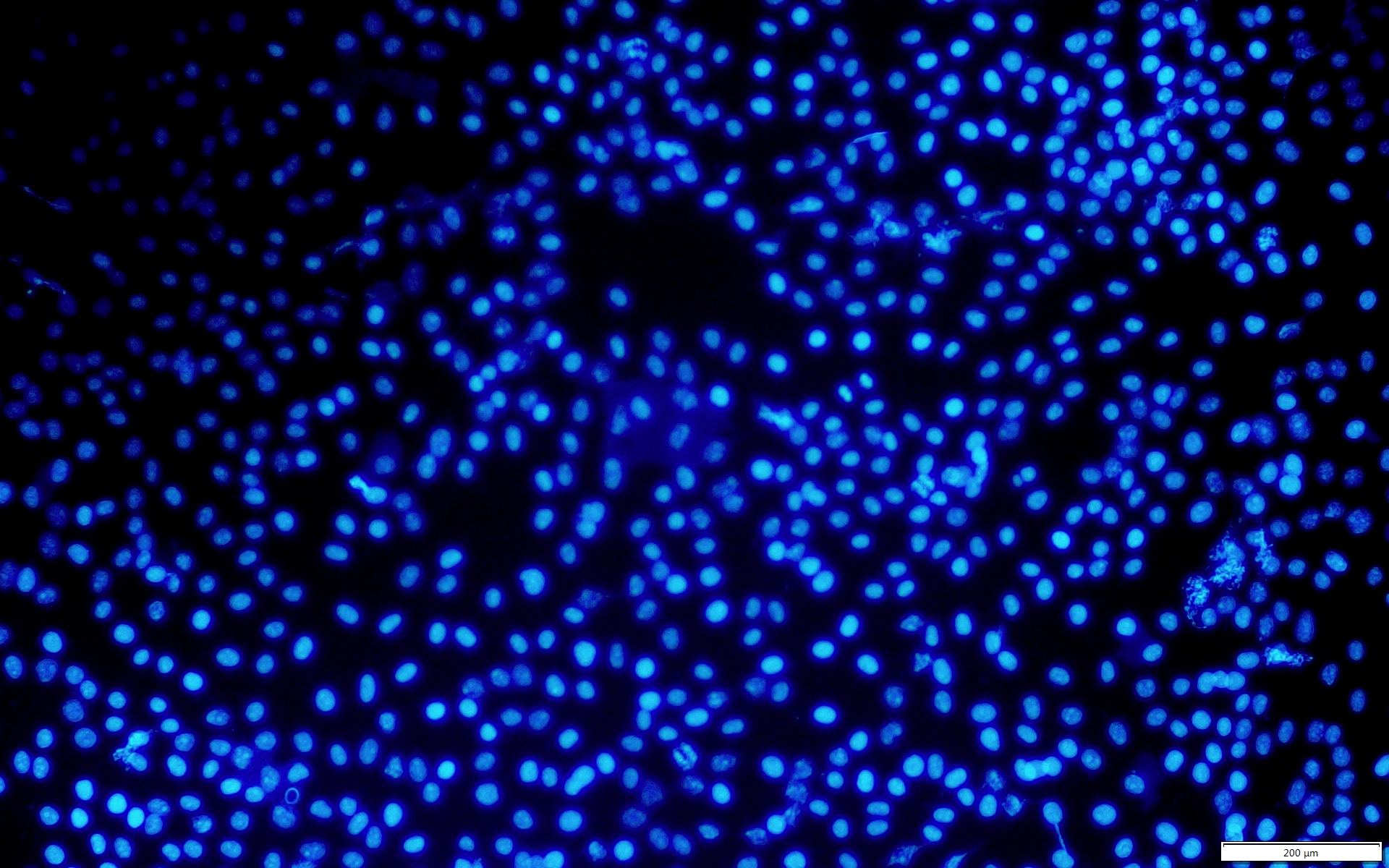

Supplement: Supplementary file 2 [file Data_Sheet_1.zip › raw data/EdU/Figure 3B/inhibitor NC+CPB2 (Hoechst).jpg]

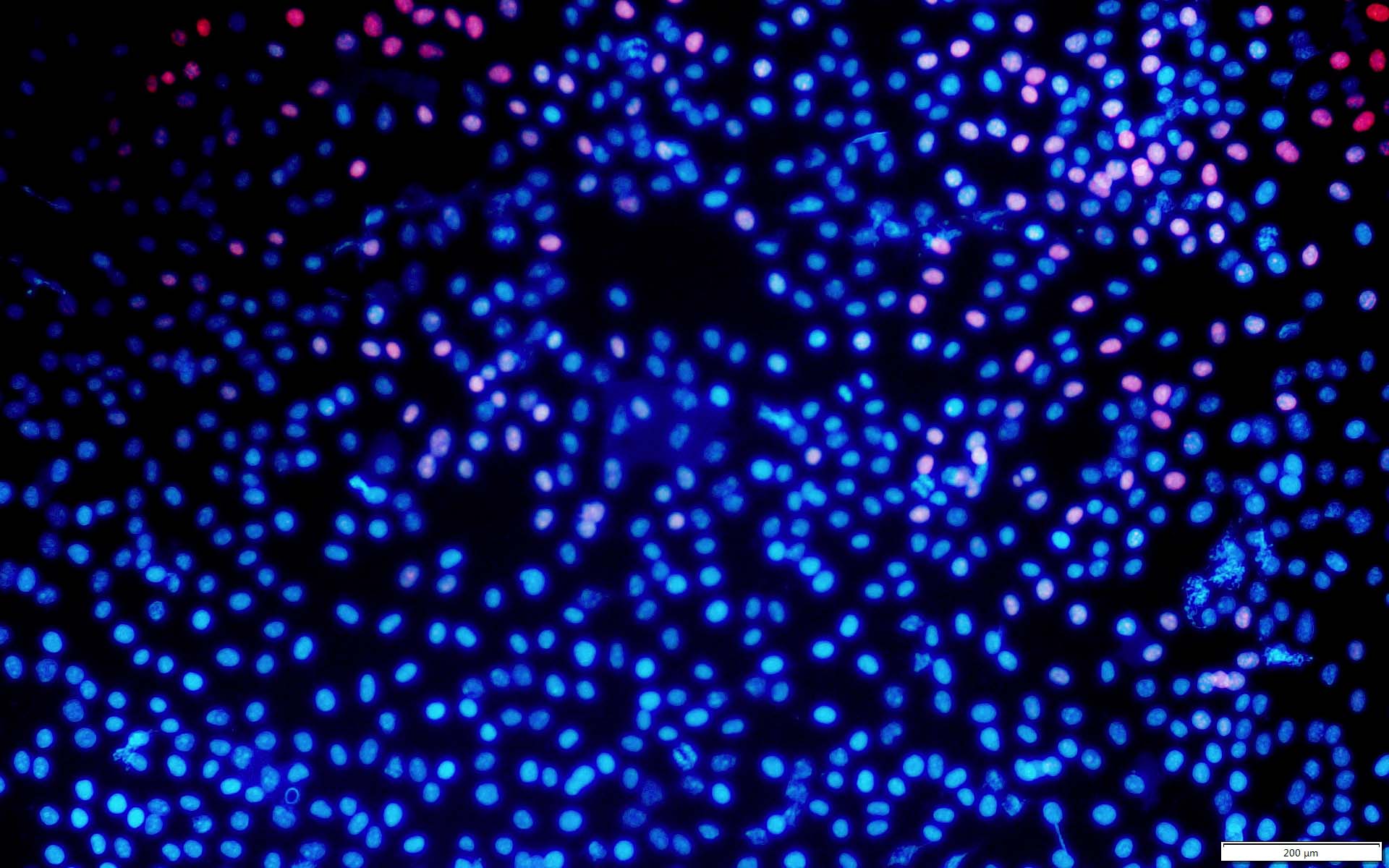

Supplement: Supplementary file 2 [file Data_Sheet_1.zip › raw data/EdU/Figure 3B/inhibitor NC+CPB2 (Merge).jpg]

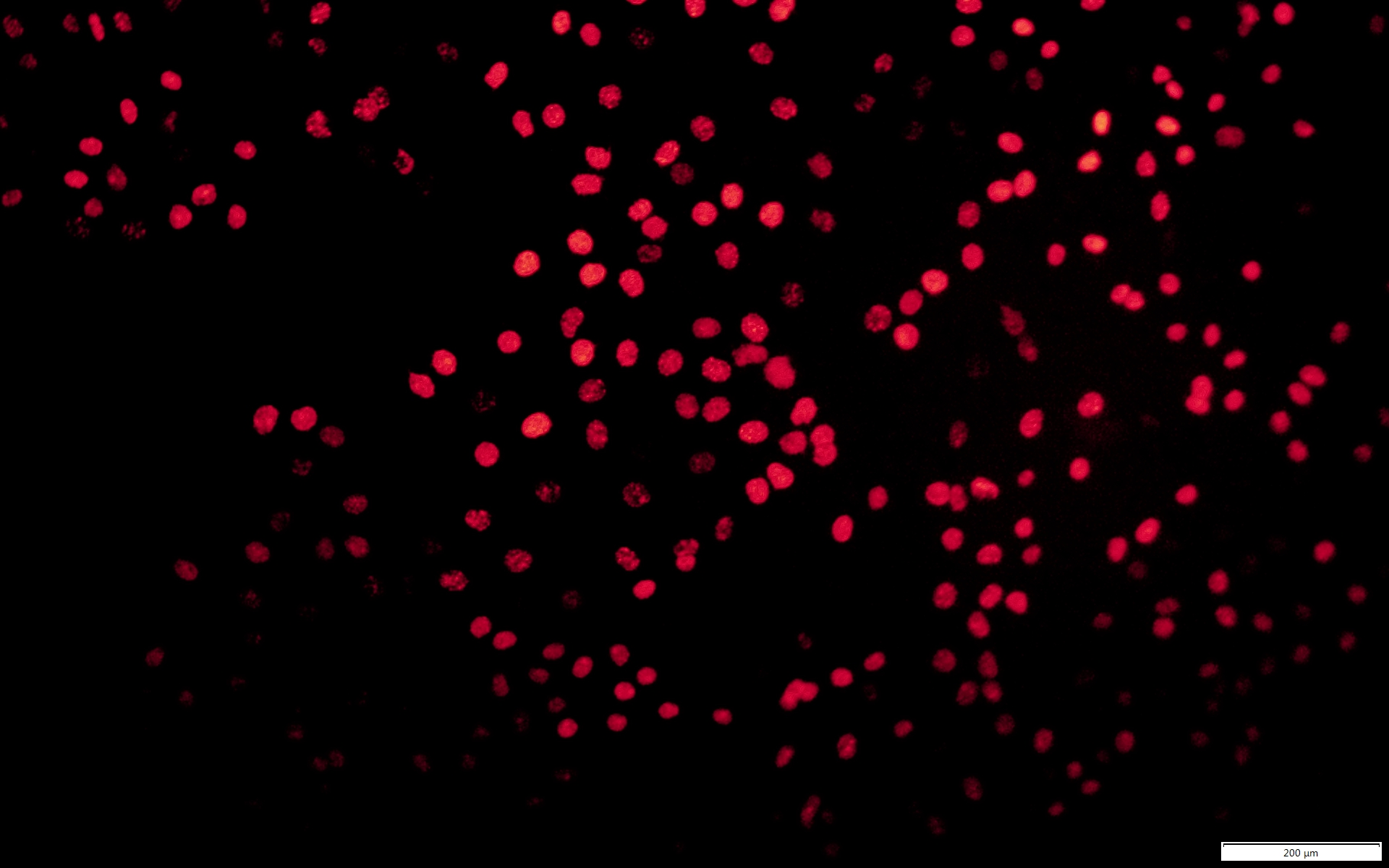

Supplement: Supplementary file 2 [file Data_Sheet_1.zip › raw data/EdU/Figure 3B/inhibitor+CPB2 (EdU).jpg]

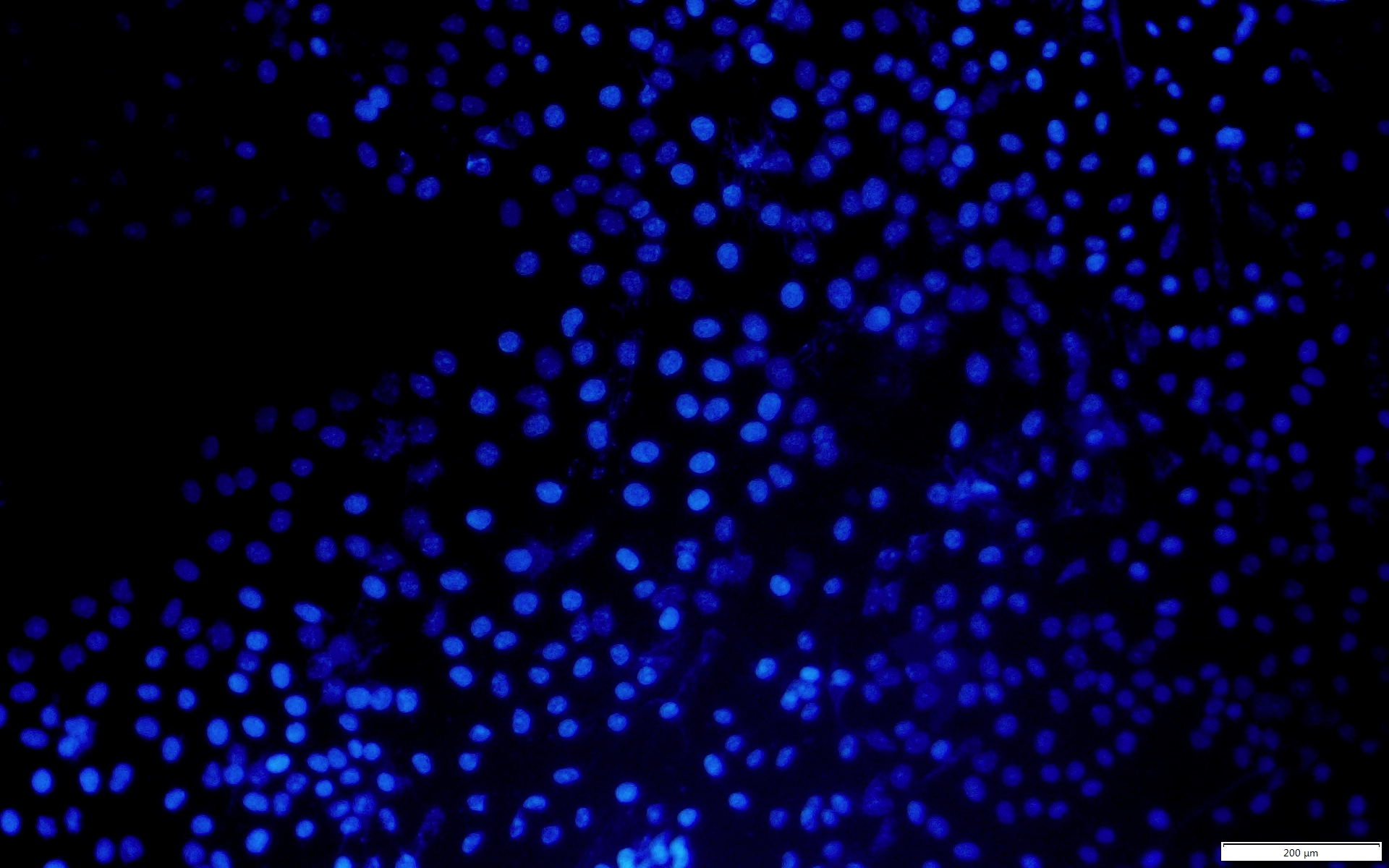

Supplement: Supplementary file 2 [file Data_Sheet_1.zip › raw data/EdU/Figure 3B/inhibitor+CPB2 (Hoechst).jpg]

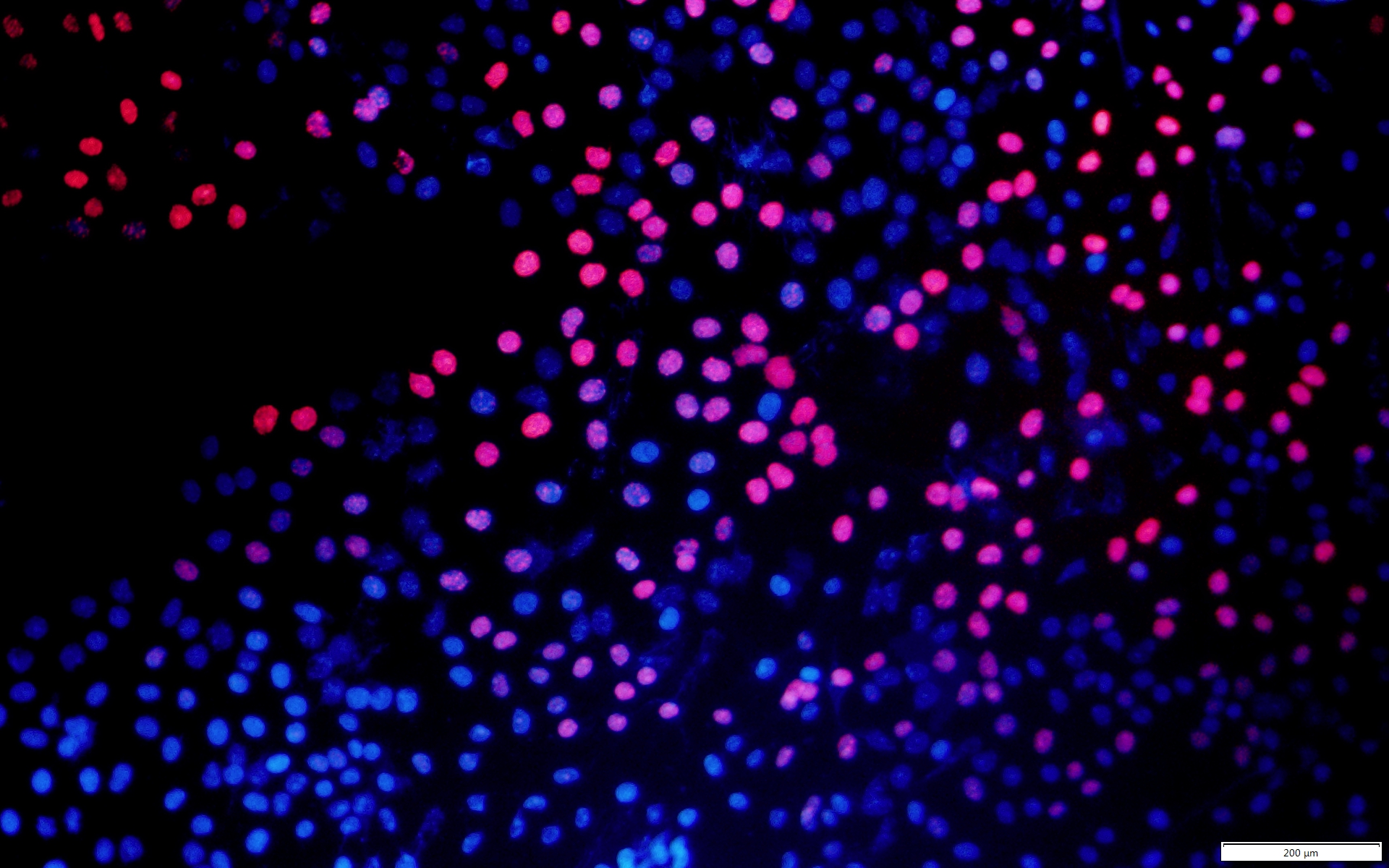

Supplement: Supplementary file 2 [file Data_Sheet_1.zip › raw data/EdU/Figure 3B/inhibitor+CPB2 (Merge).jpg]

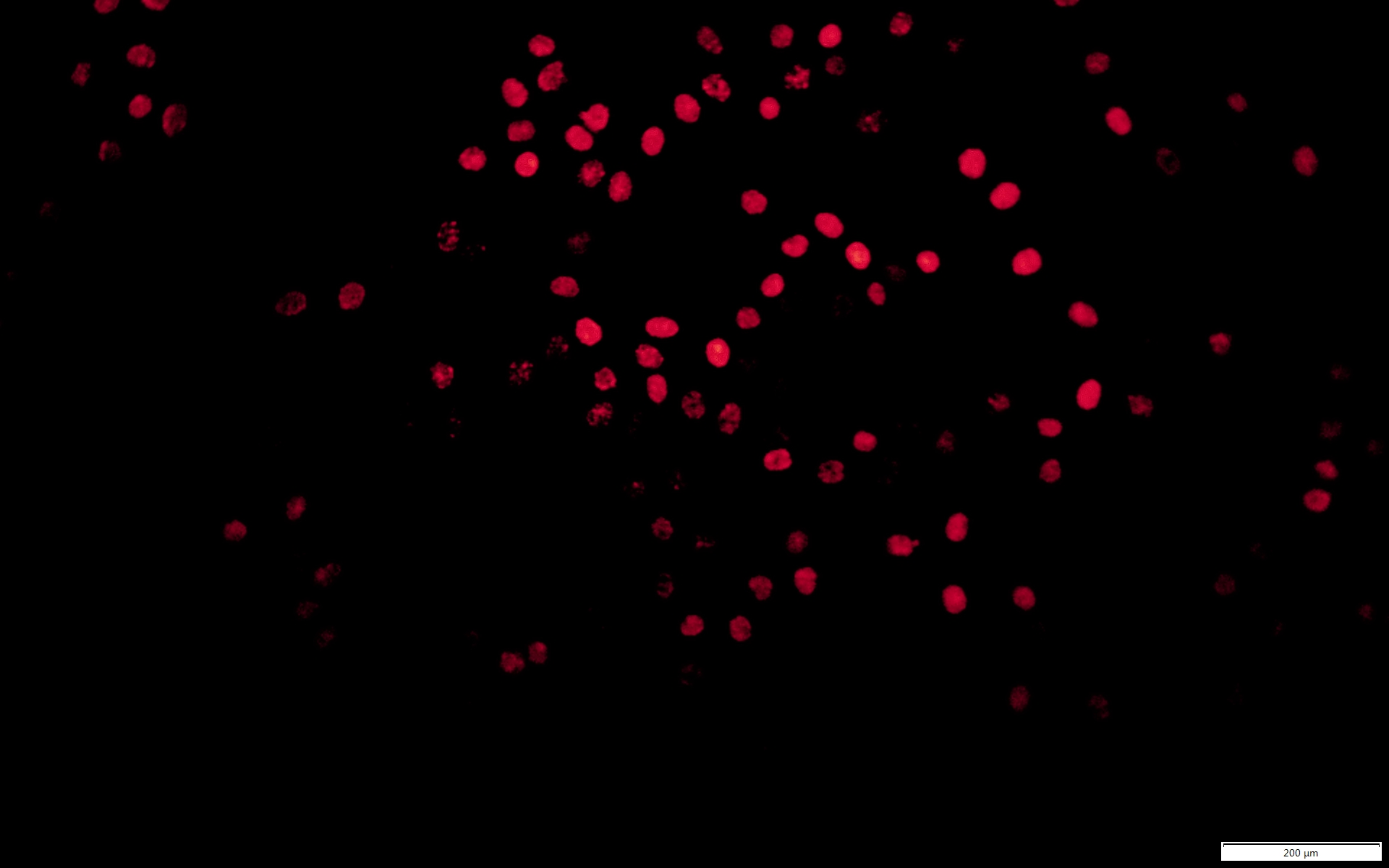

Supplement: Supplementary file 2 [file Data_Sheet_1.zip › raw data/EdU/Figure 3B/mimic NC+CPB2 (EdU).jpg]

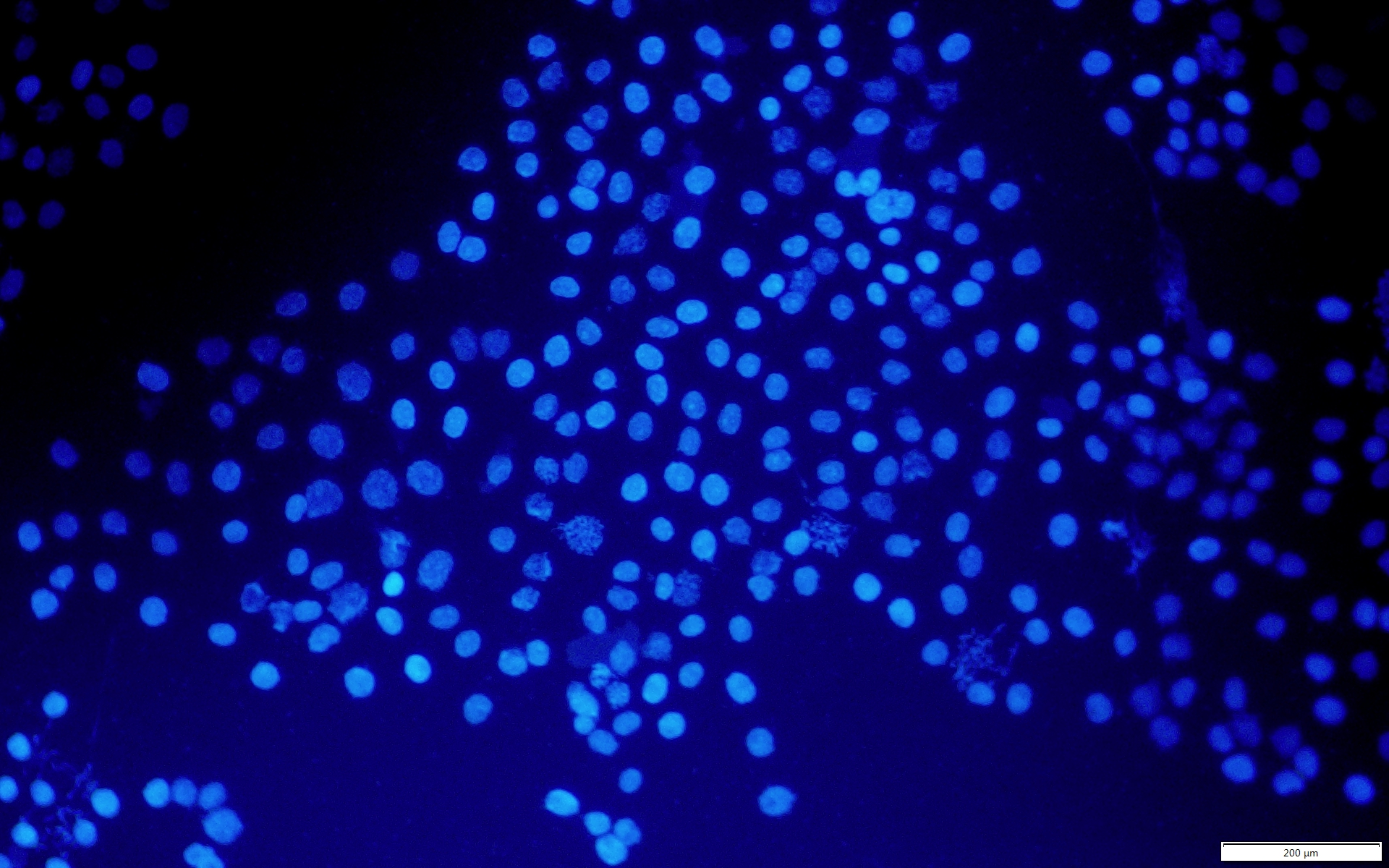

Supplement: Supplementary file 2 [file Data_Sheet_1.zip › raw data/EdU/Figure 3B/mimic NC+CPB2 (Hoechst).jpg]

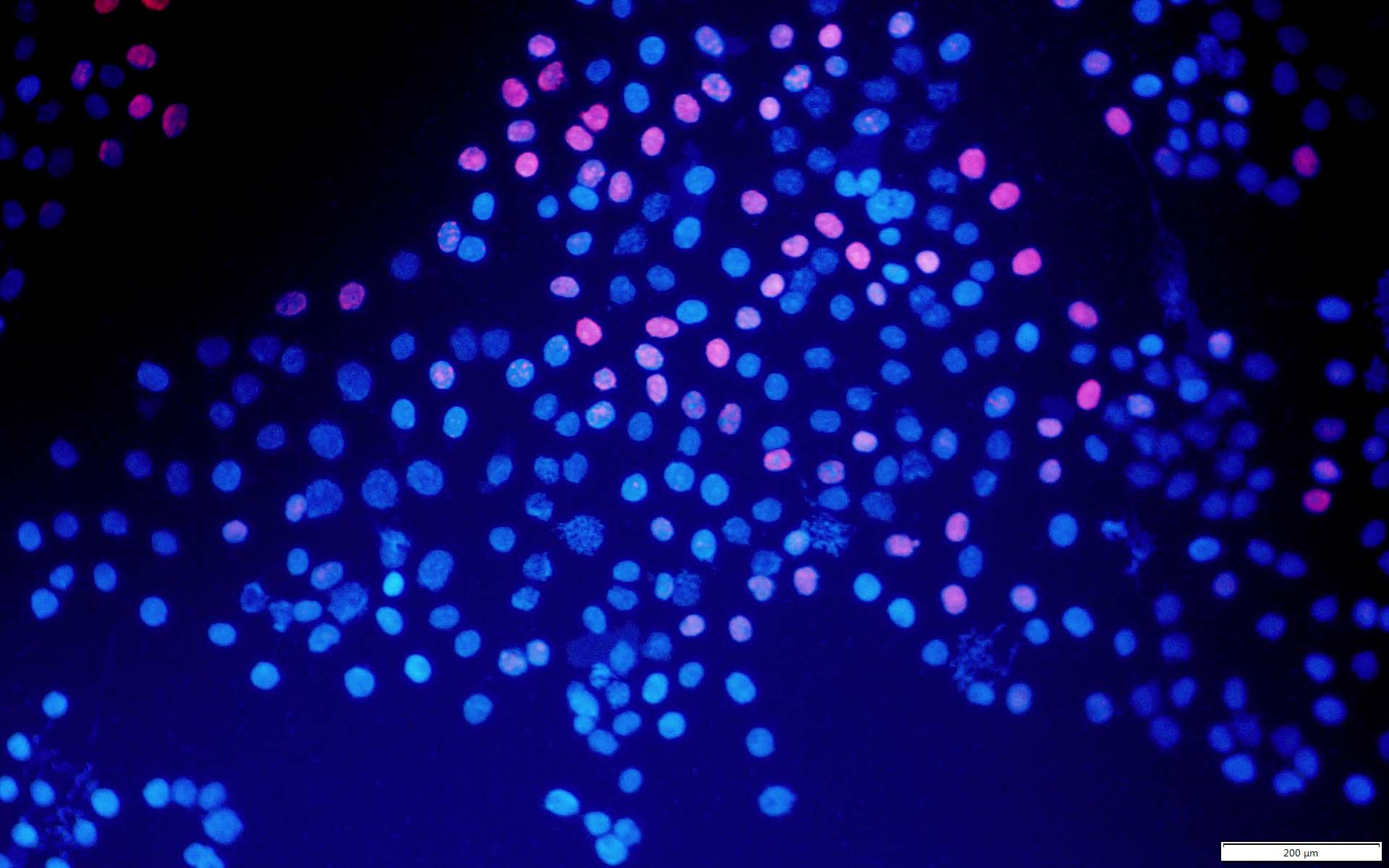

Supplement: Supplementary file 2 [file Data_Sheet_1.zip › raw data/EdU/Figure 3B/mimic NC+CPB2 (Merge).jpg]

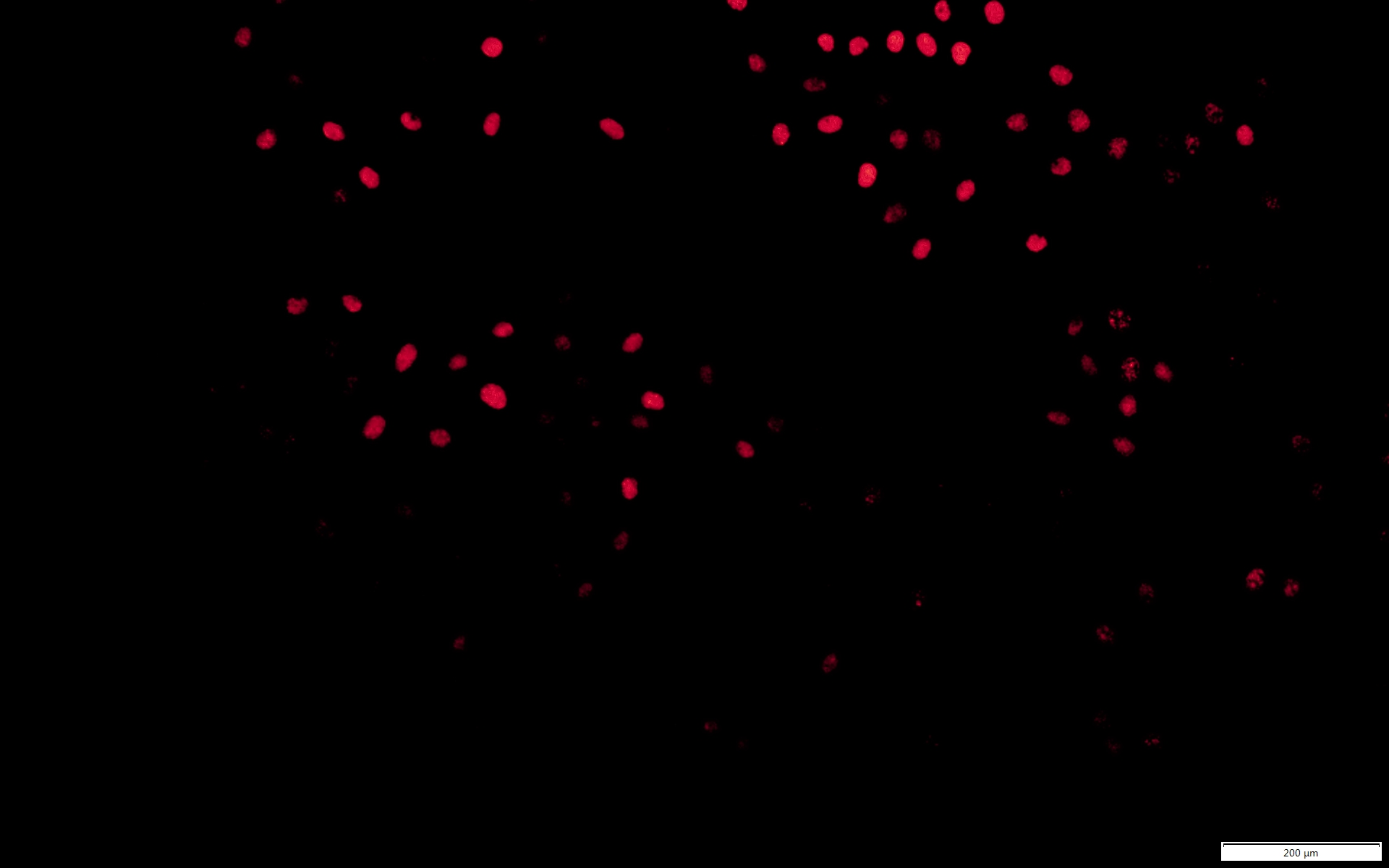

Supplement: Supplementary file 2 [file Data_Sheet_1.zip › raw data/EdU/Figure 3B/mimic+CPB2 (EdU).jpg]

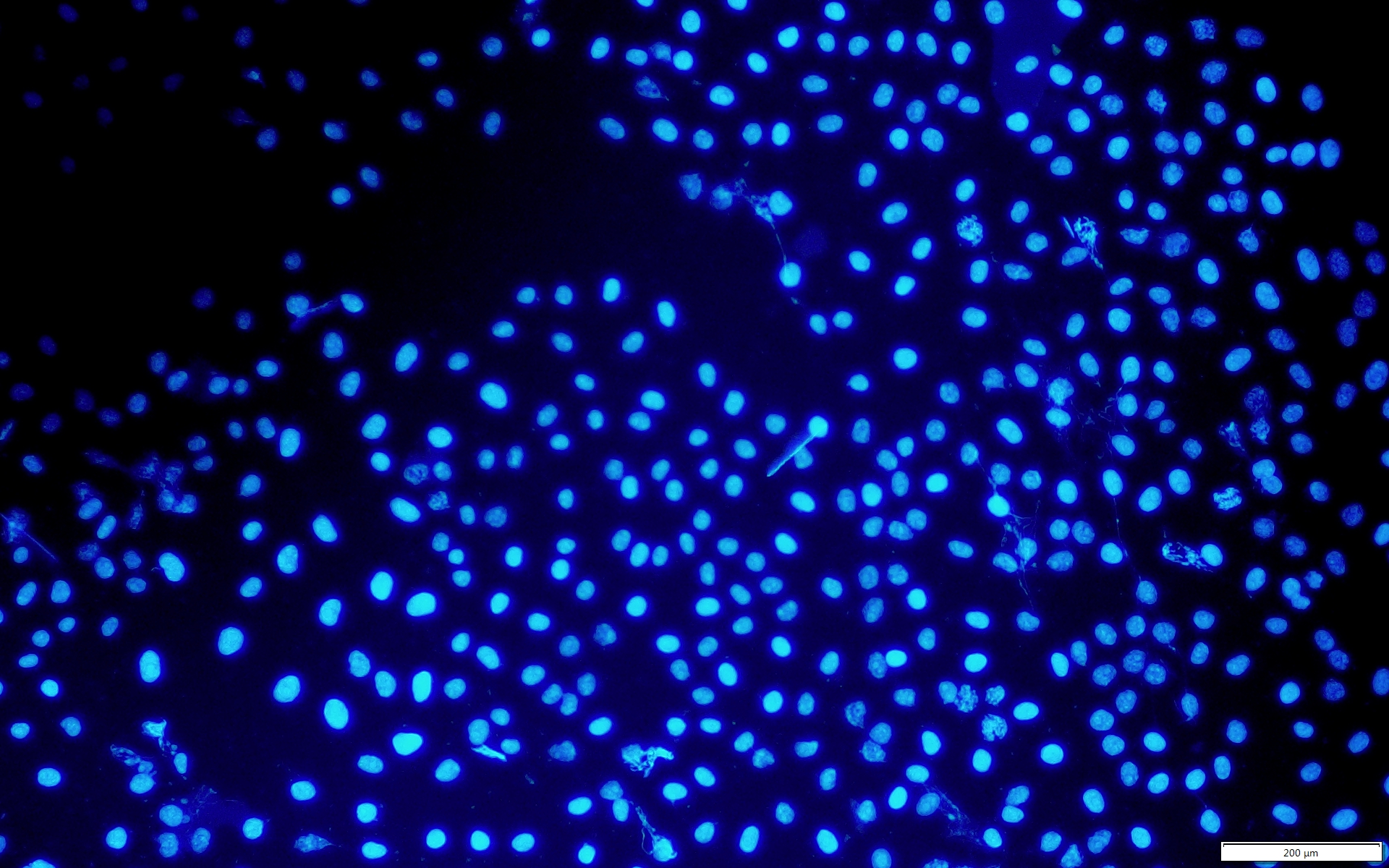

Supplement: Supplementary file 2 [file Data_Sheet_1.zip › raw data/EdU/Figure 3B/mimic+CPB2 (Hoechst).jpg]

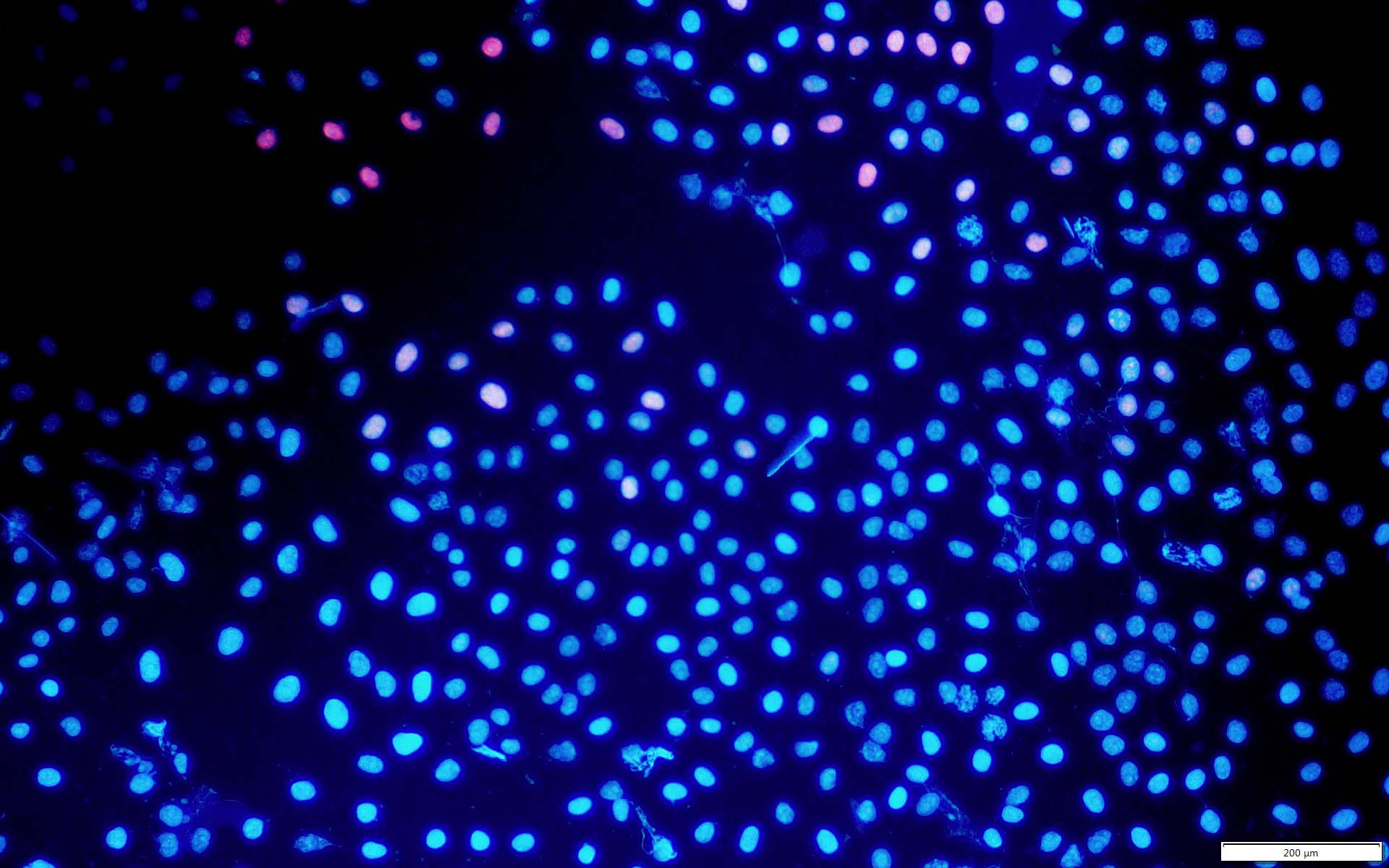

Supplement: Supplementary file 2 [file Data_Sheet_1.zip › raw data/EdU/Figure 3B/mimic+CPB2 (Merge).jpg]

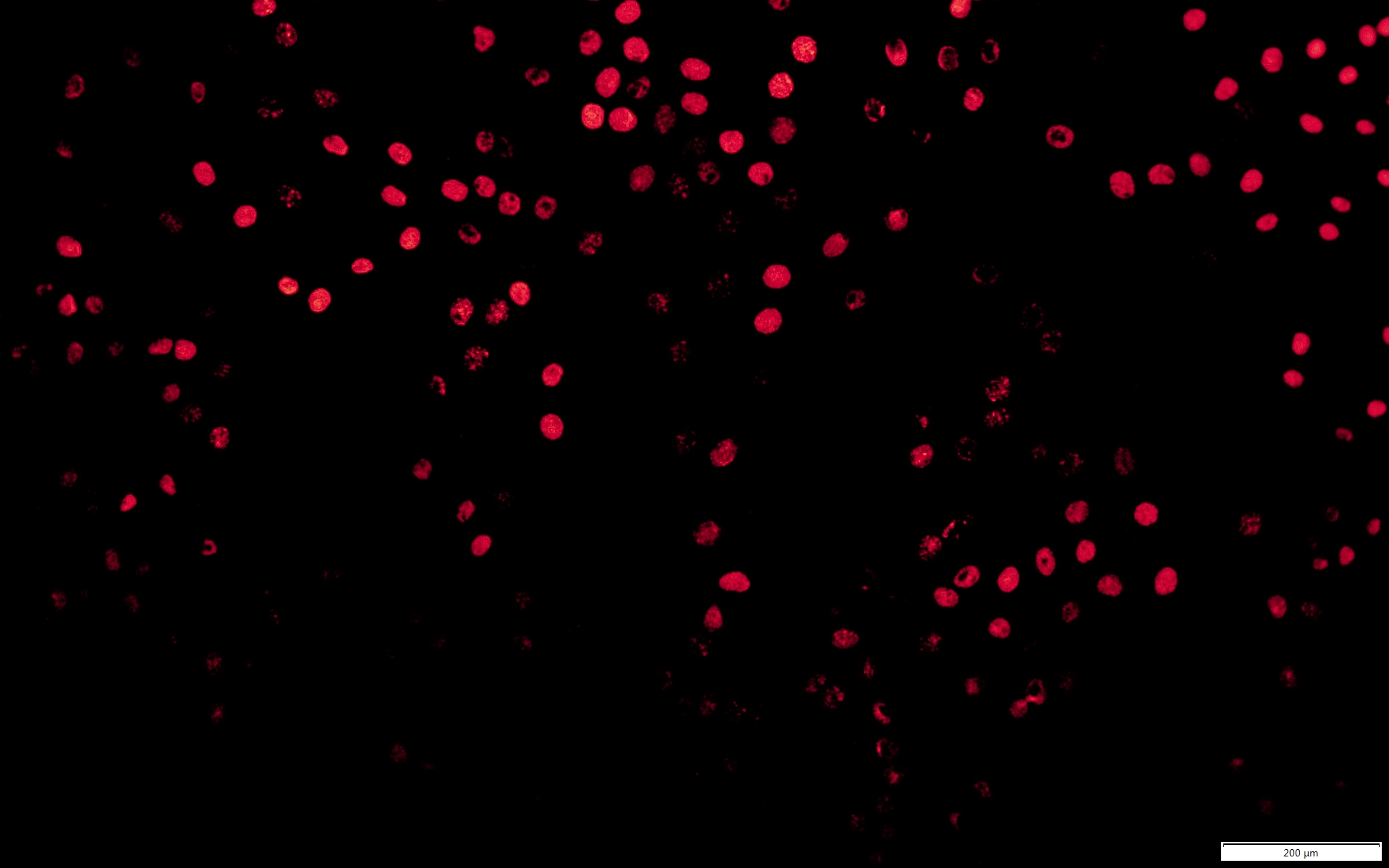

Supplement: Supplementary file 2 [file Data_Sheet_1.zip › raw data/EdU/Figure 6B/pcDNA3.1+CPB2 (EdU).jpg]

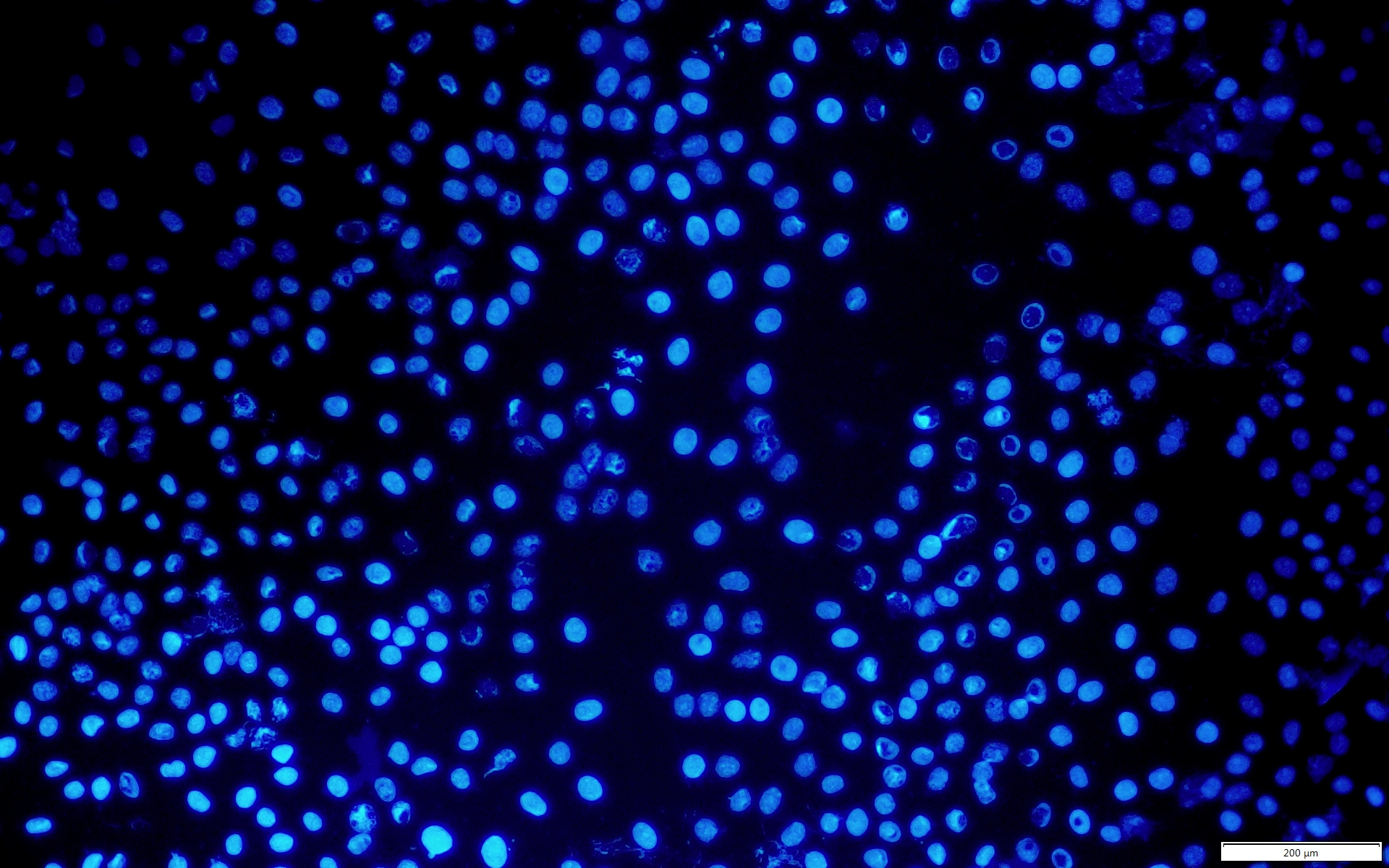

Supplement: Supplementary file 2 [file Data_Sheet_1.zip › raw data/EdU/Figure 6B/pcDNA3.1+CPB2 (Hoechst).jpg]

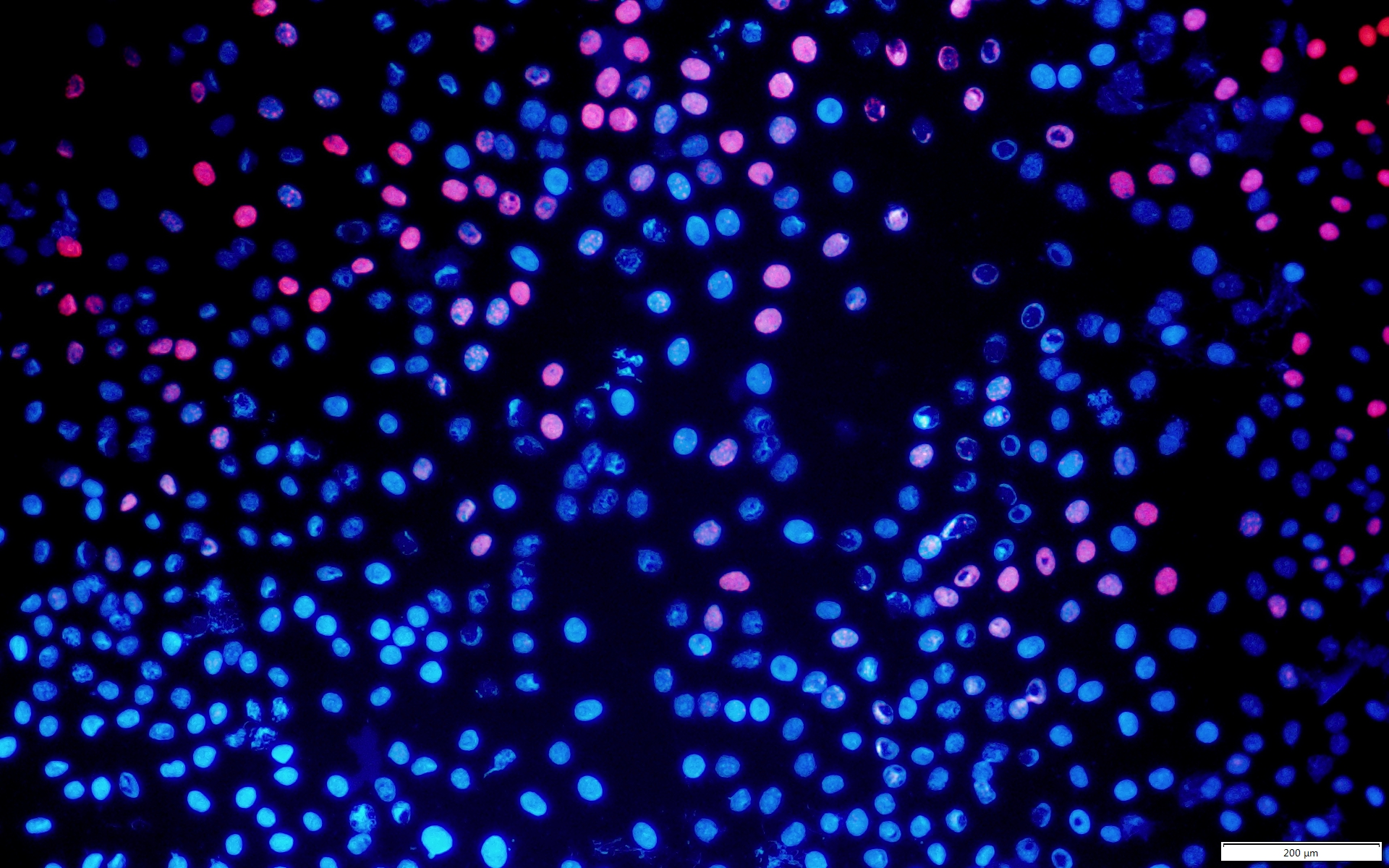

Supplement: Supplementary file 2 [file Data_Sheet_1.zip › raw data/EdU/Figure 6B/pcDNA3.1+CPB2 (Merge).jpg]

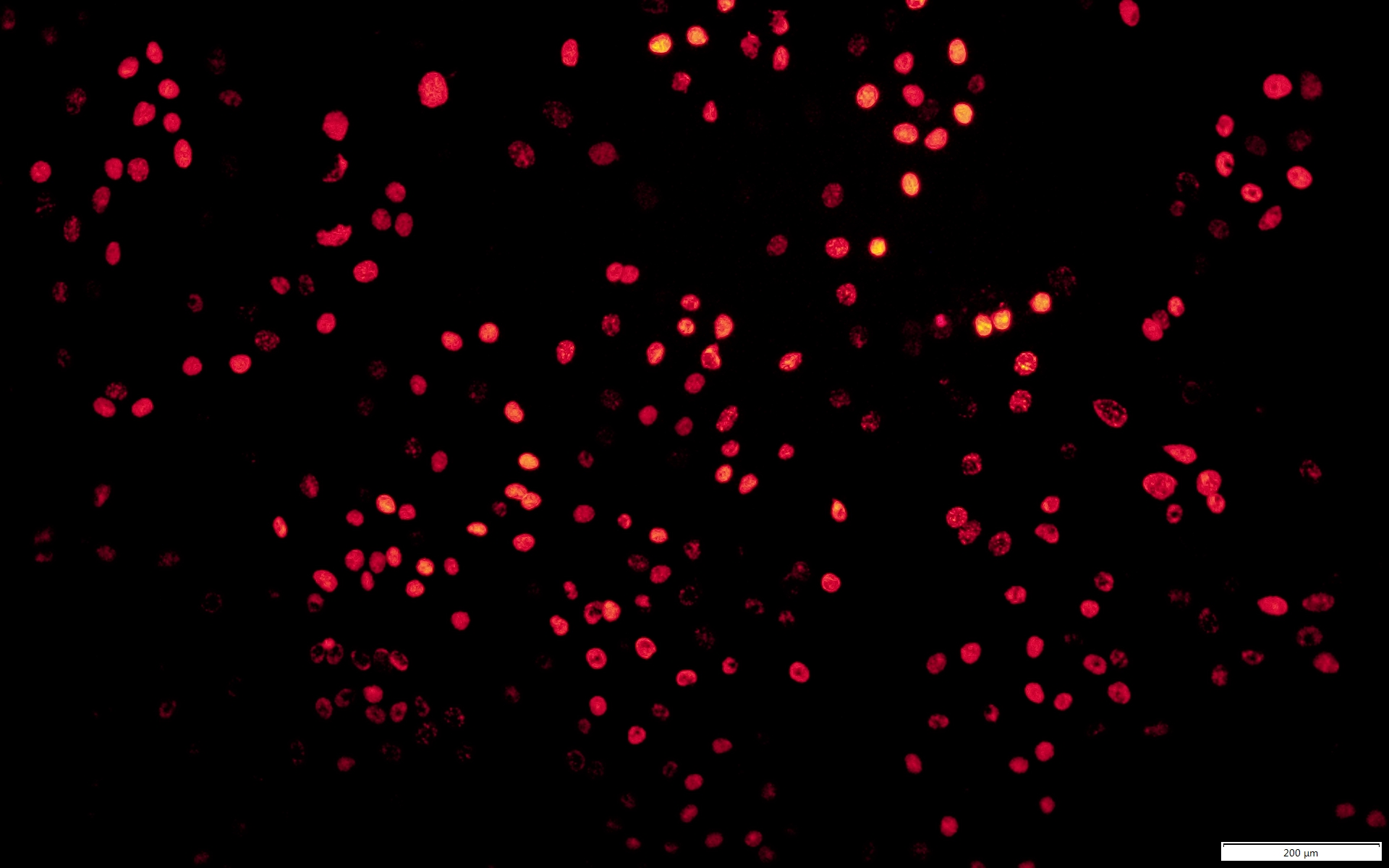

Supplement: Supplementary file 2 [file Data_Sheet_1.zip › raw data/EdU/Figure 6B/pc-PSME3+CPB2 (EdU).jpg]

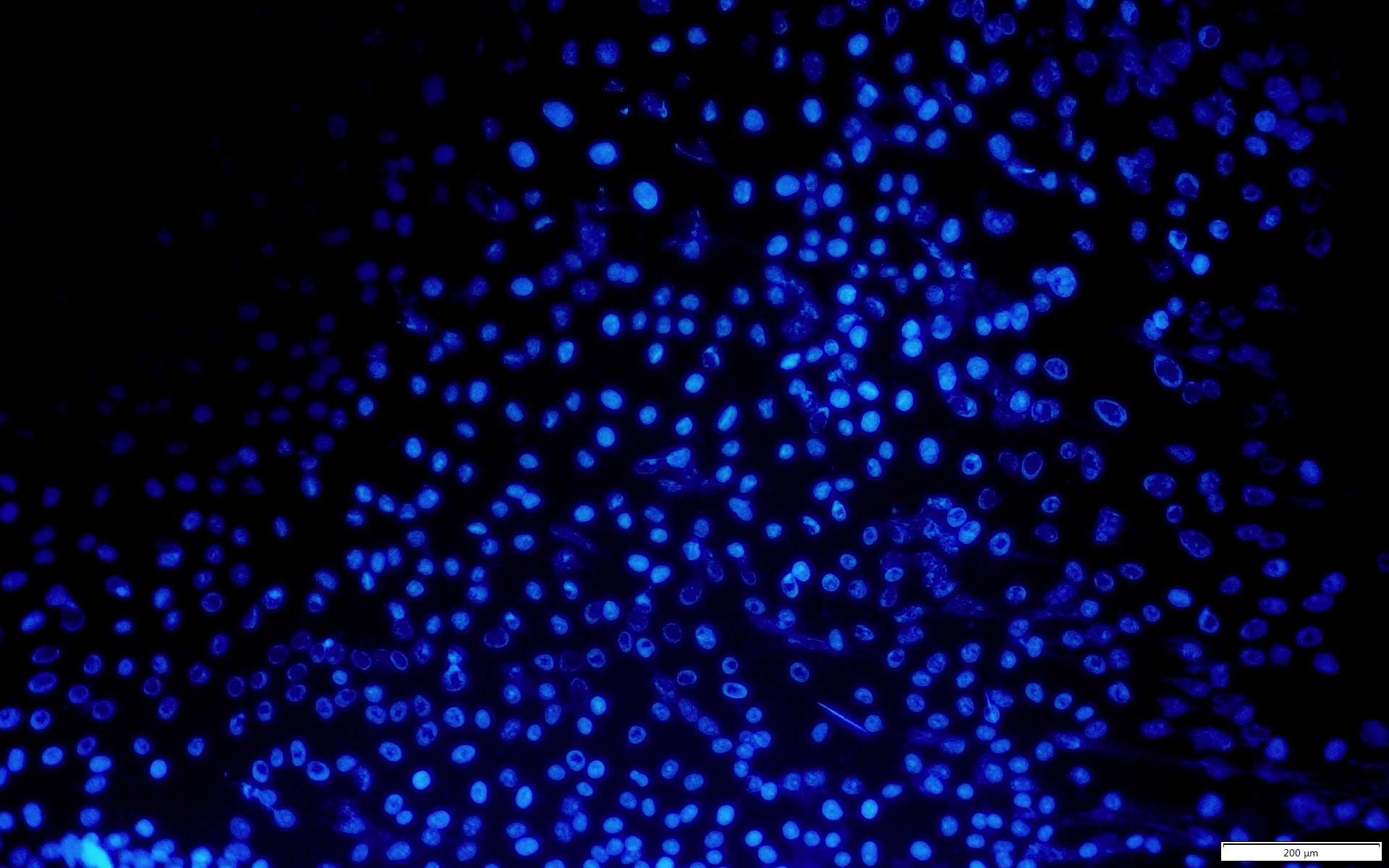

Supplement: Supplementary file 2 [file Data_Sheet_1.zip › raw data/EdU/Figure 6B/pc-PSME3+CPB2 (Hoechst).jpg]

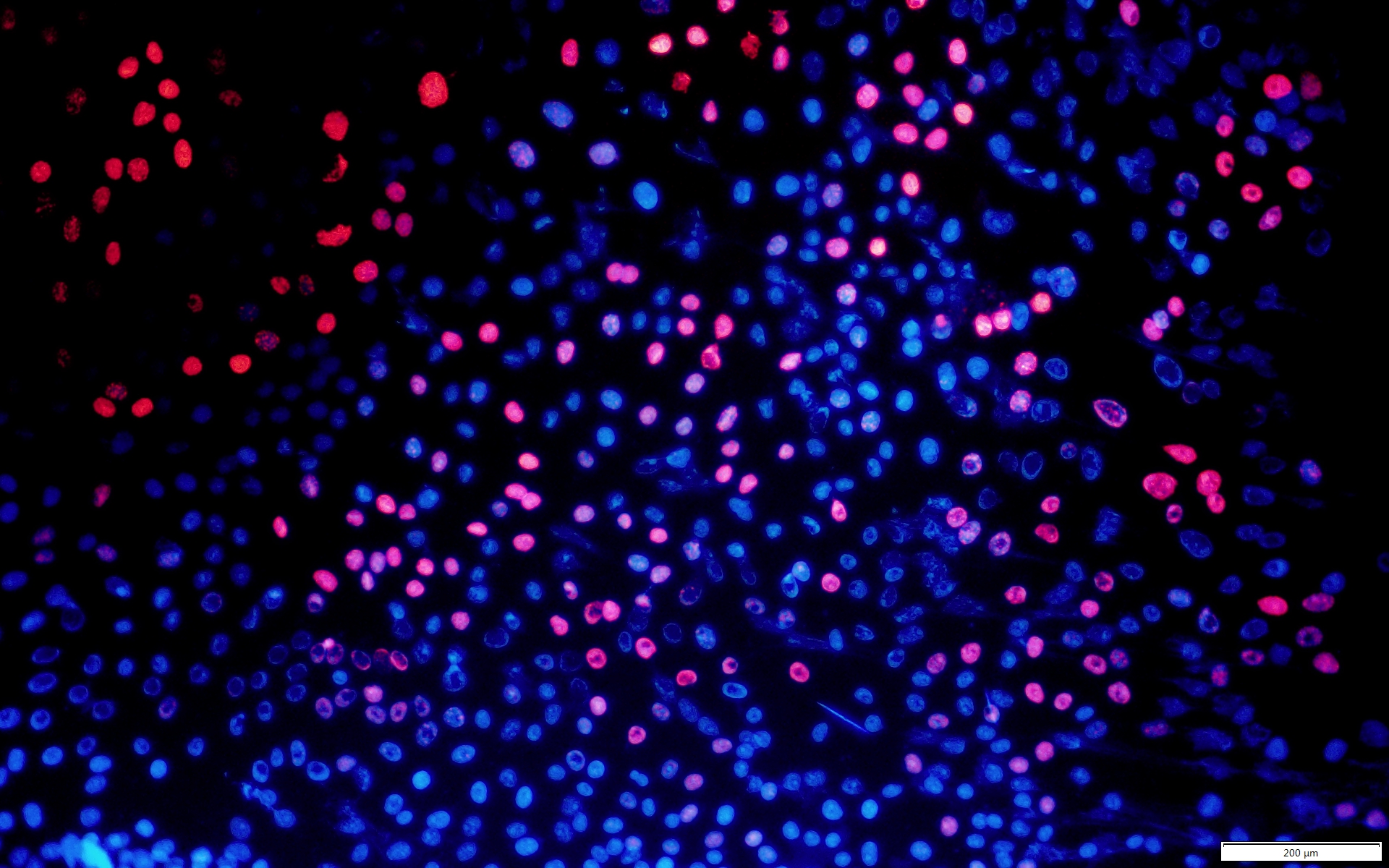

Supplement: Supplementary file 2 [file Data_Sheet_1.zip › raw data/EdU/Figure 6B/pc-PSME3+CPB2 (Merge).jpg]

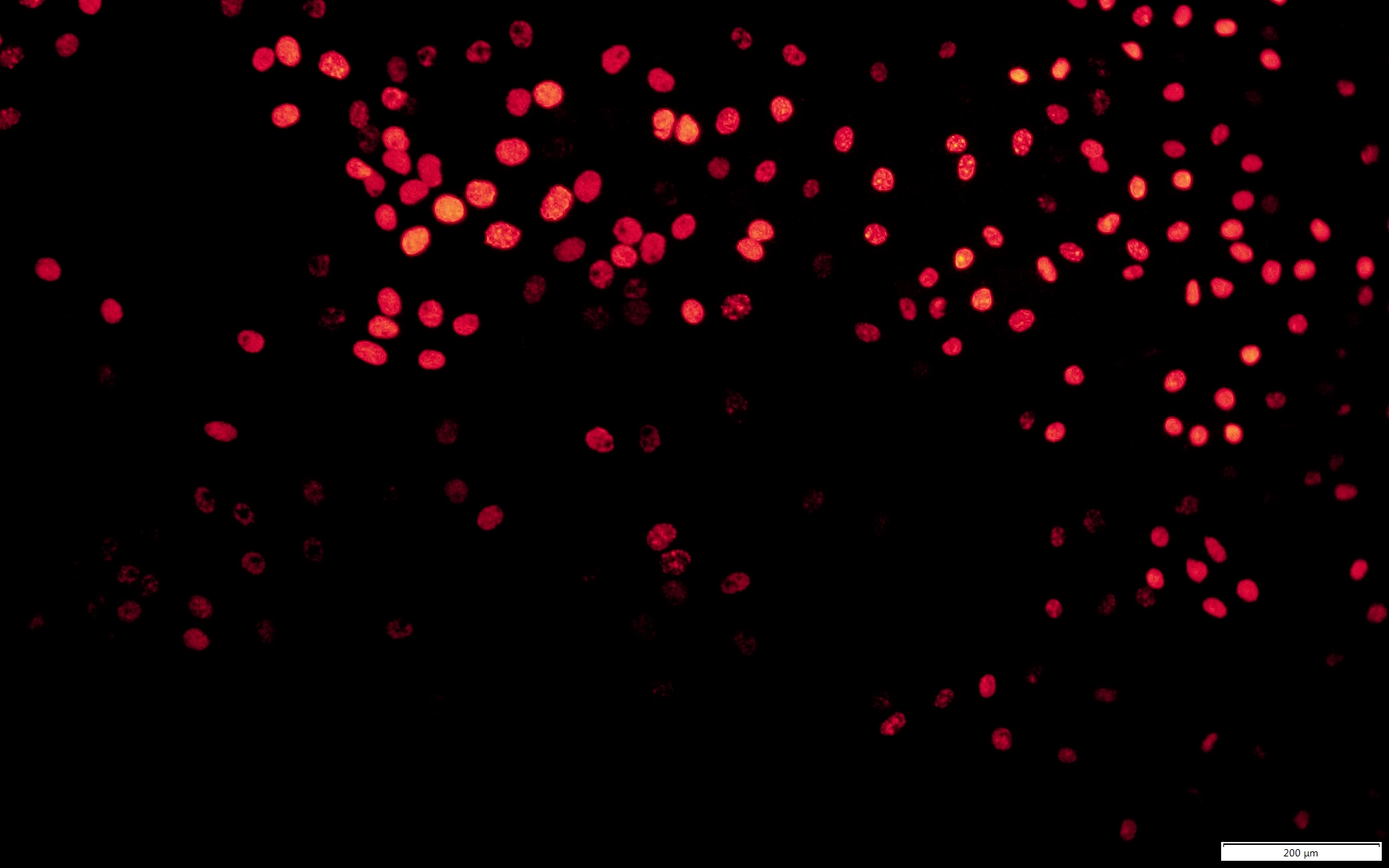

Supplement: Supplementary file 2 [file Data_Sheet_1.zip › raw data/EdU/Figure 6B/si-NC+CPB2 (EdU).jpg]

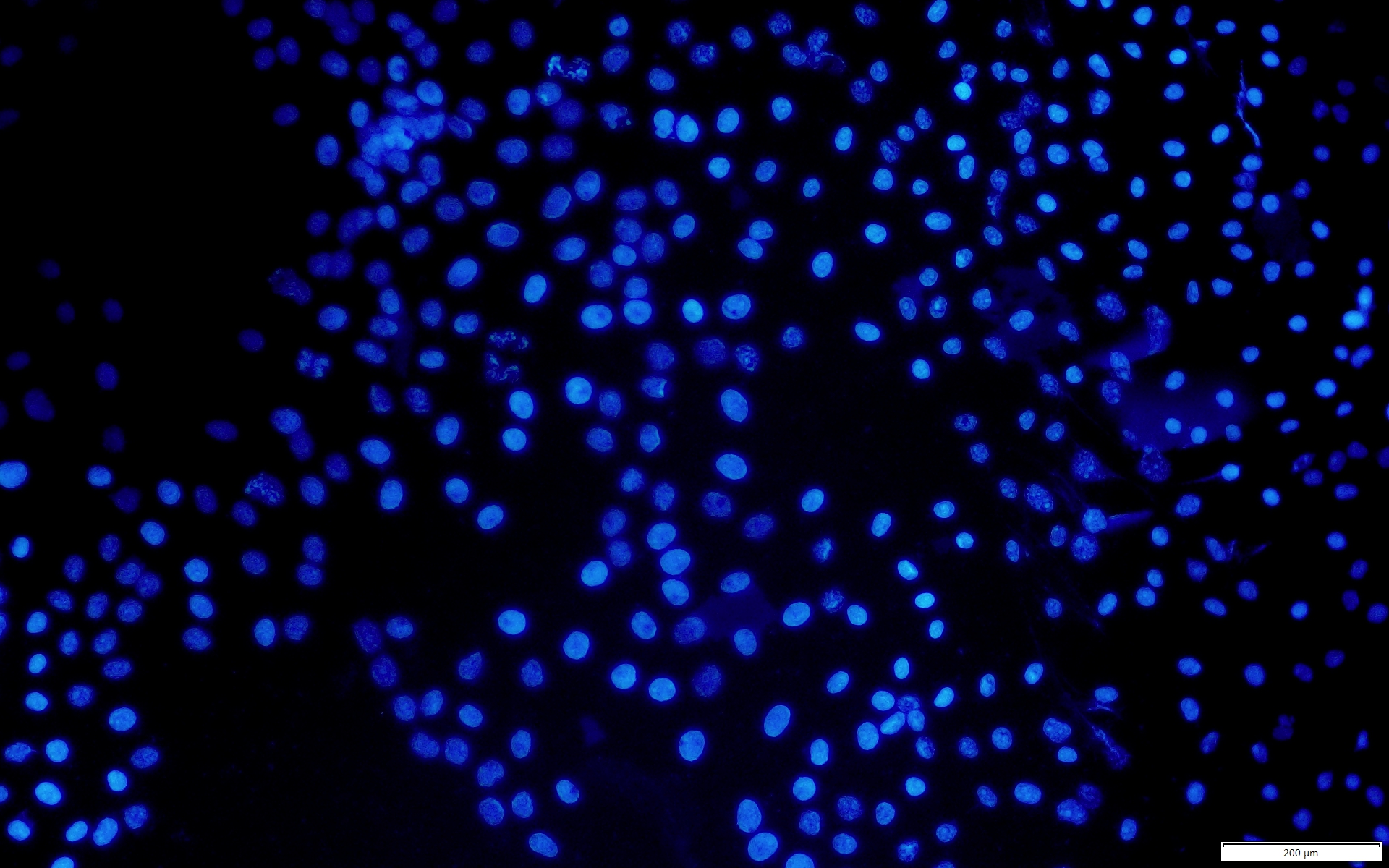

Supplement: Supplementary file 2 [file Data_Sheet_1.zip › raw data/EdU/Figure 6B/si-NC+CPB2 (Hoechst).jpg]

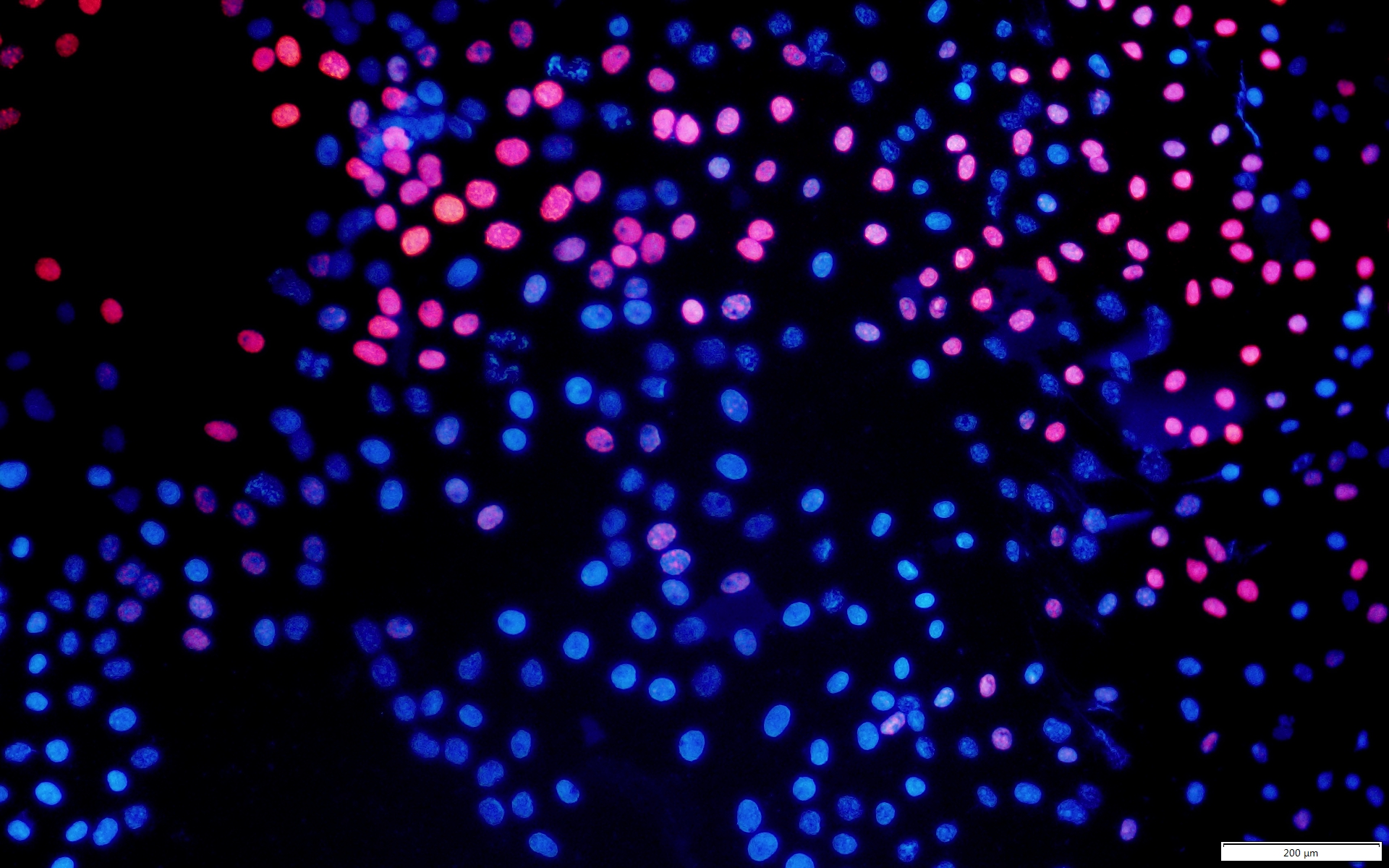

Supplement: Supplementary file 2 [file Data_Sheet_1.zip › raw data/EdU/Figure 6B/si-NC+CPB2 (Merge).jpg]

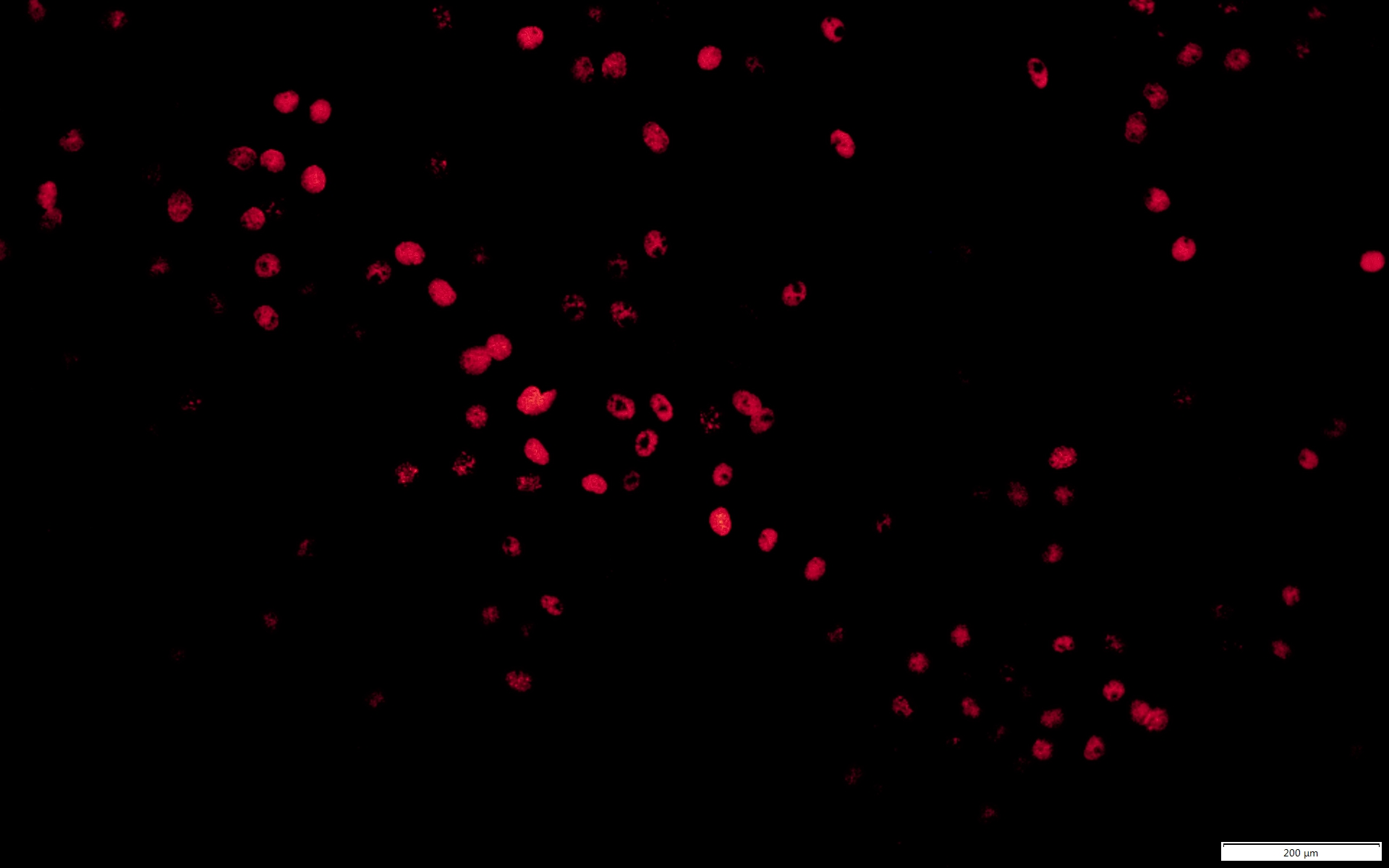

Supplement: Supplementary file 2 [file Data_Sheet_1.zip › raw data/EdU/Figure 6B/si-PSME3+CPB2 (EdU).jpg]

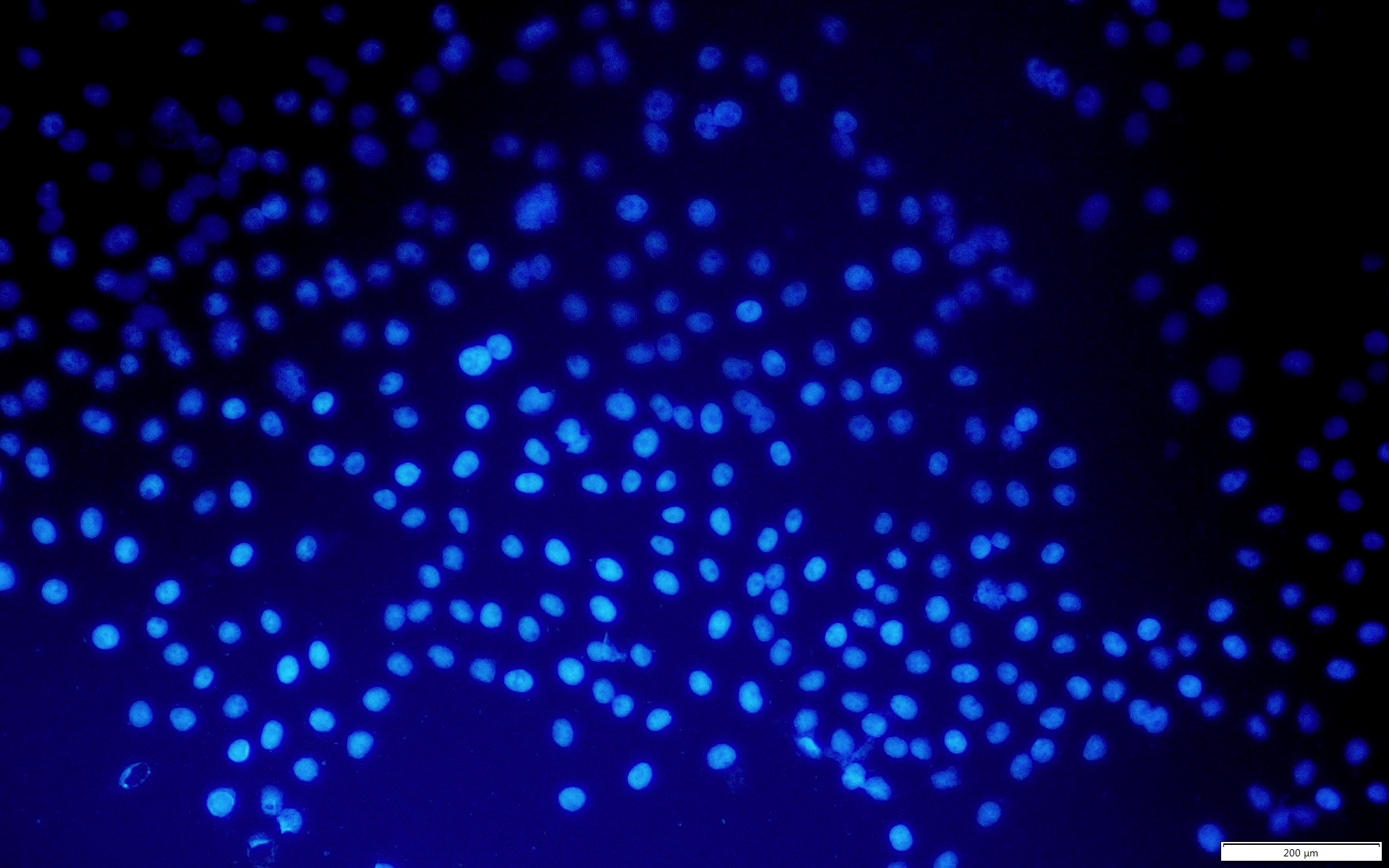

Supplement: Supplementary file 2 [file Data_Sheet_1.zip › raw data/EdU/Figure 6B/si-PSME3+CPB2 (Hoechst).jpg]

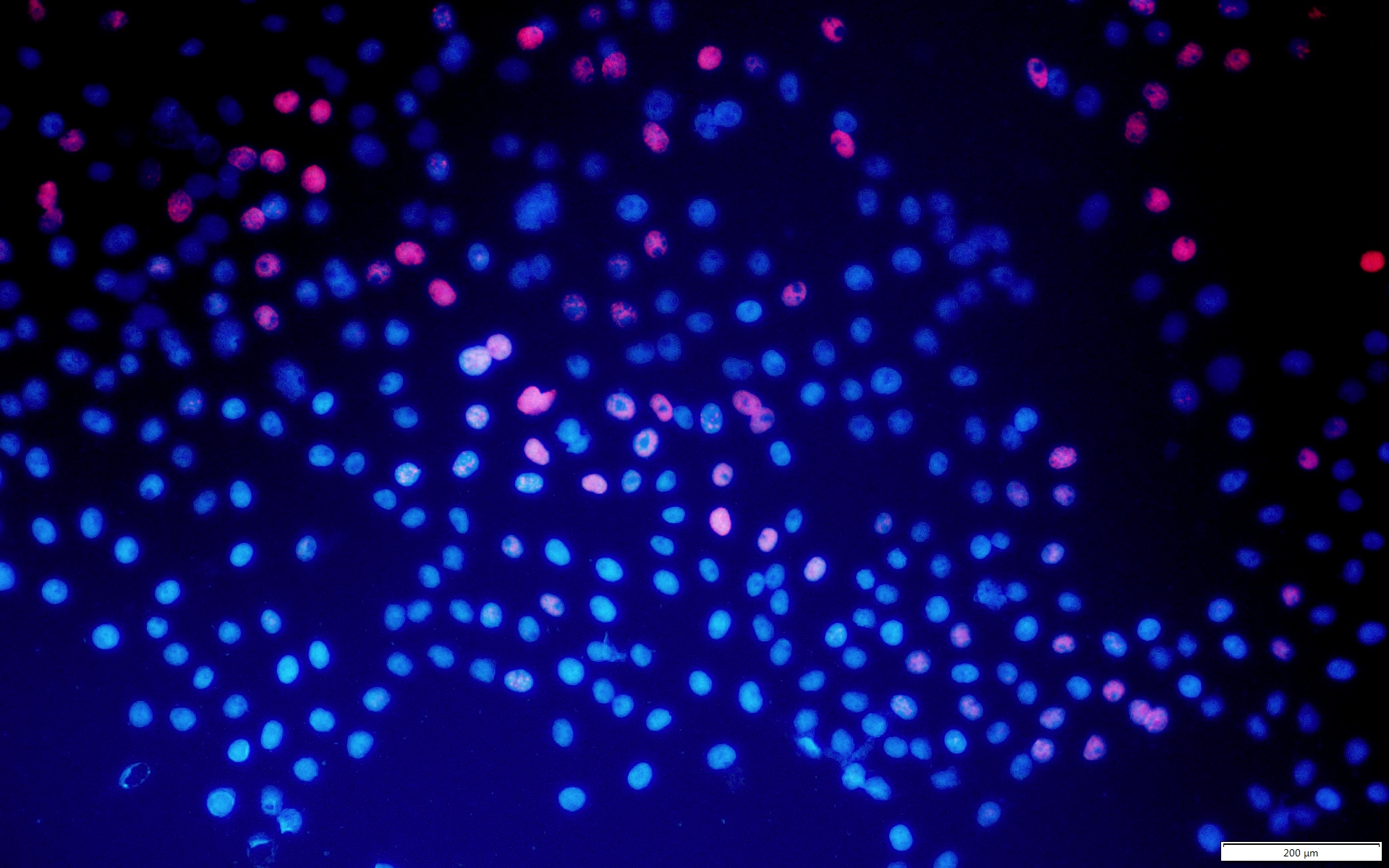

Supplement: Supplementary file 2 [file Data_Sheet_1.zip › raw data/EdU/Figure 6B/si-PSME3+CPB2 (Merge).jpg]

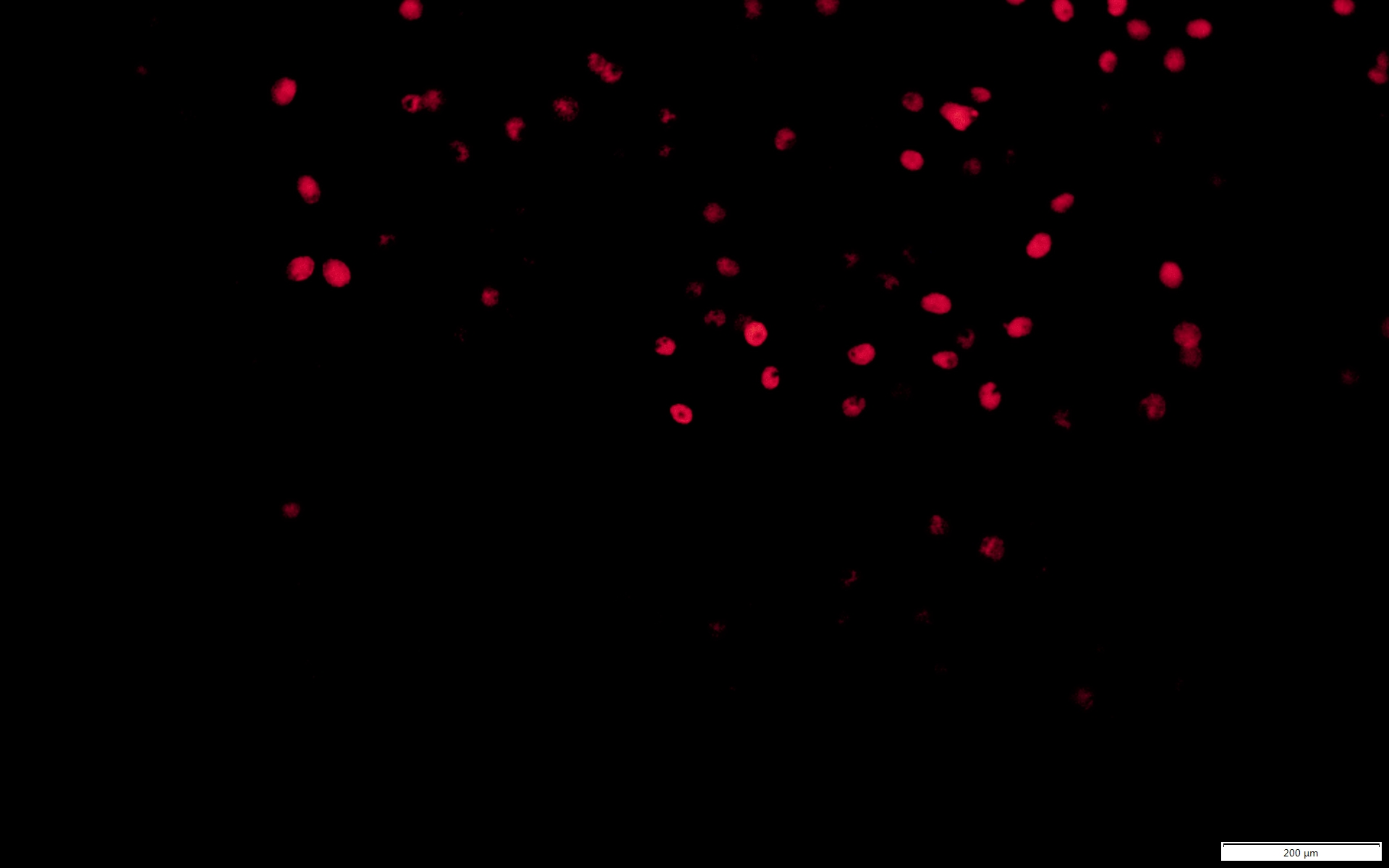

Supplement: Supplementary file 2 [file Data_Sheet_1.zip › raw data/EdU/Figure 7B/mimic (EdU).jpg]

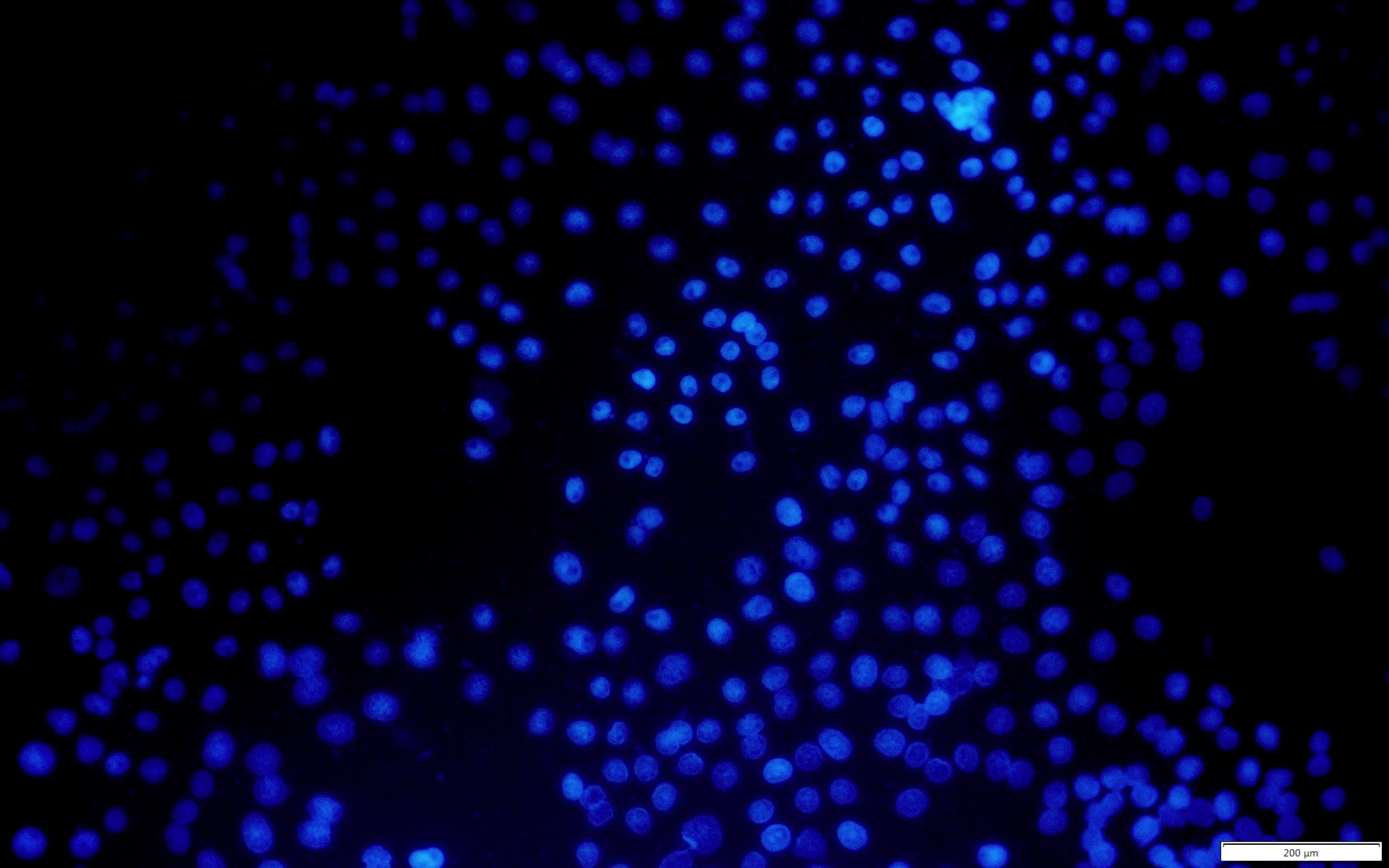

Supplement: Supplementary file 2 [file Data_Sheet_1.zip › raw data/EdU/Figure 7B/mimic (Hoechst).jpg]

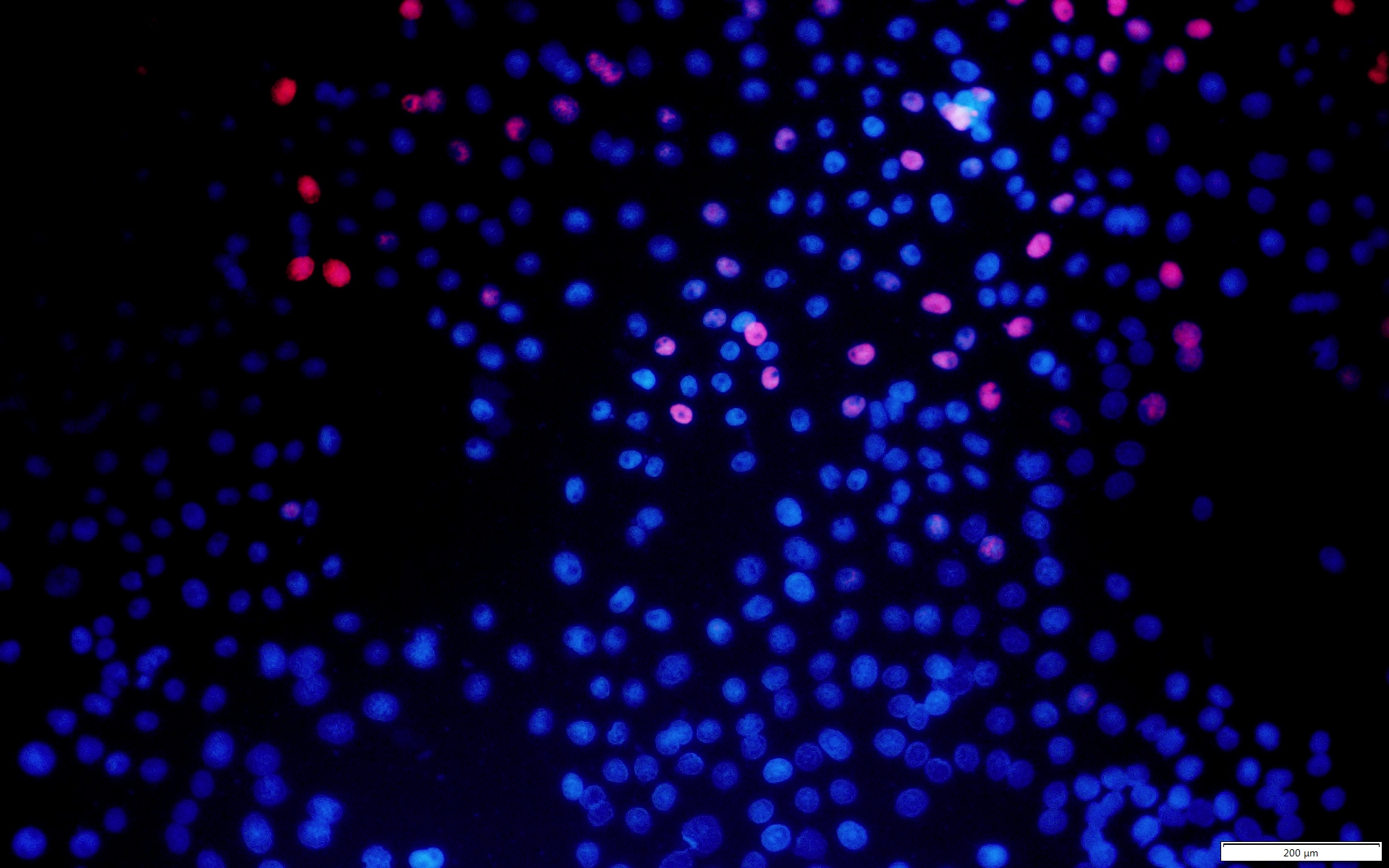

Supplement: Supplementary file 2 [file Data_Sheet_1.zip › raw data/EdU/Figure 7B/mimic (Merge).jpg]

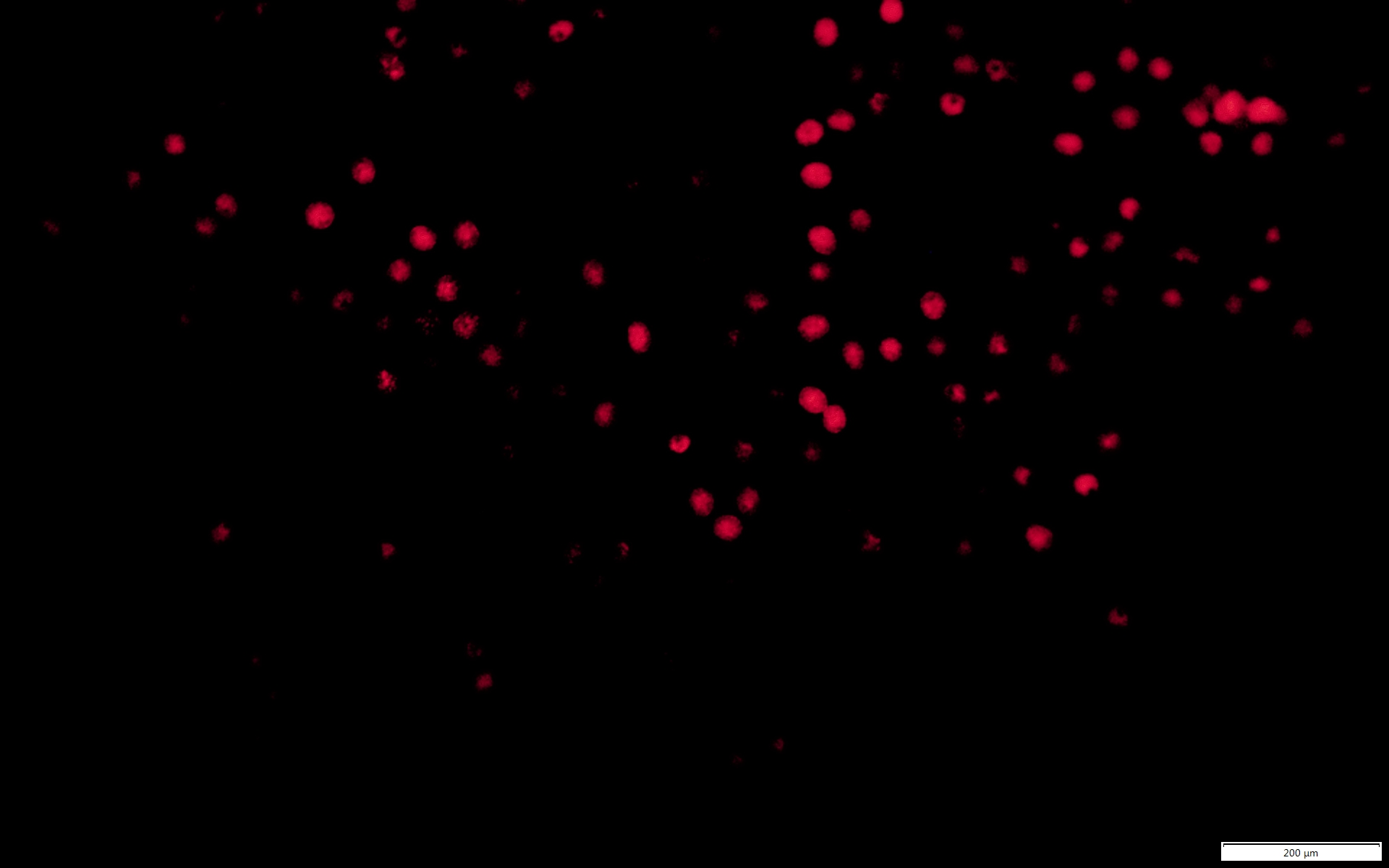

Supplement: Supplementary file 2 [file Data_Sheet_1.zip › raw data/EdU/Figure 7B/mimic+pcDNA (EdU).jpg]

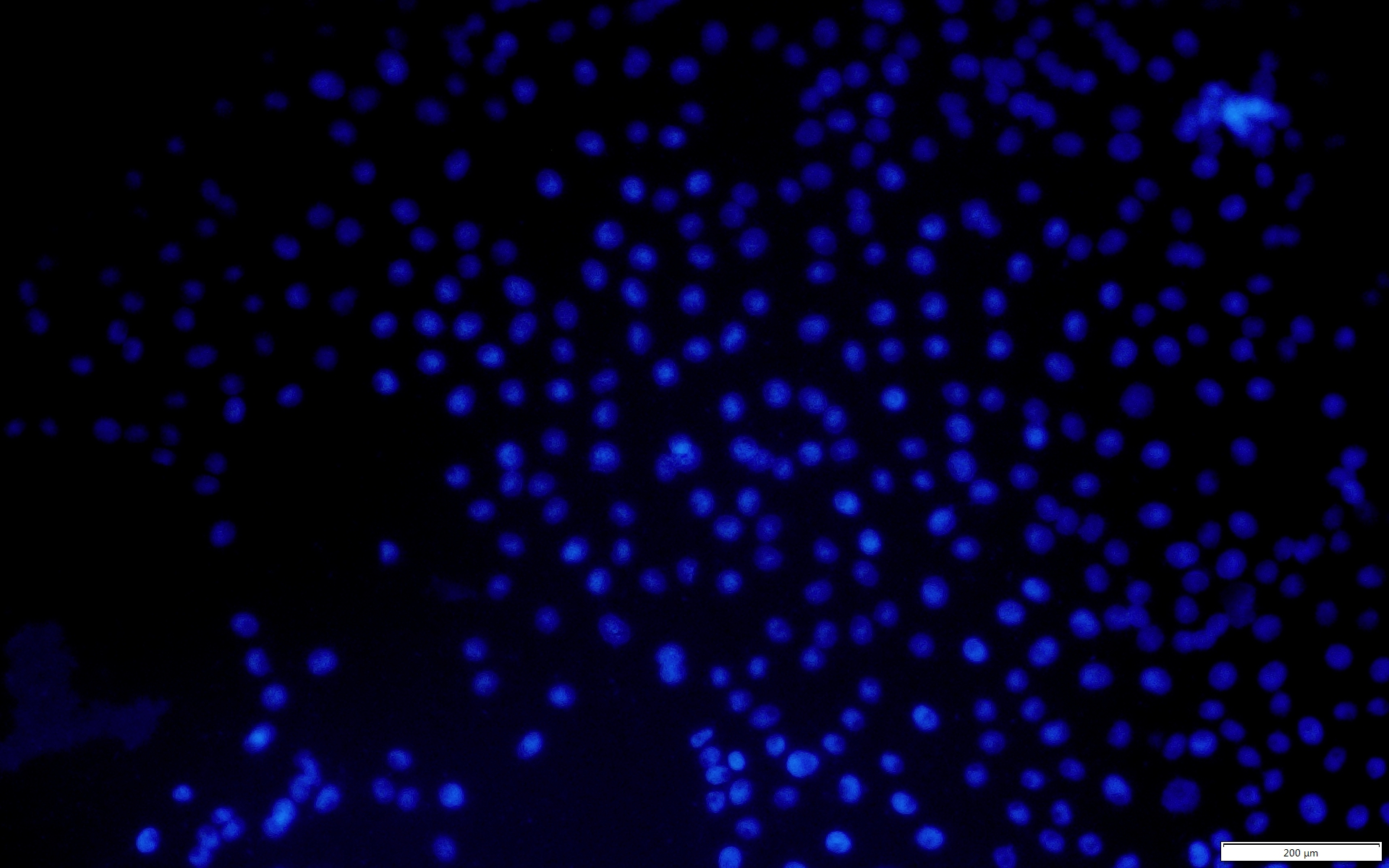

Supplement: Supplementary file 2 [file Data_Sheet_1.zip › raw data/EdU/Figure 7B/mimic+pcDNA (Hoechst).jpg]

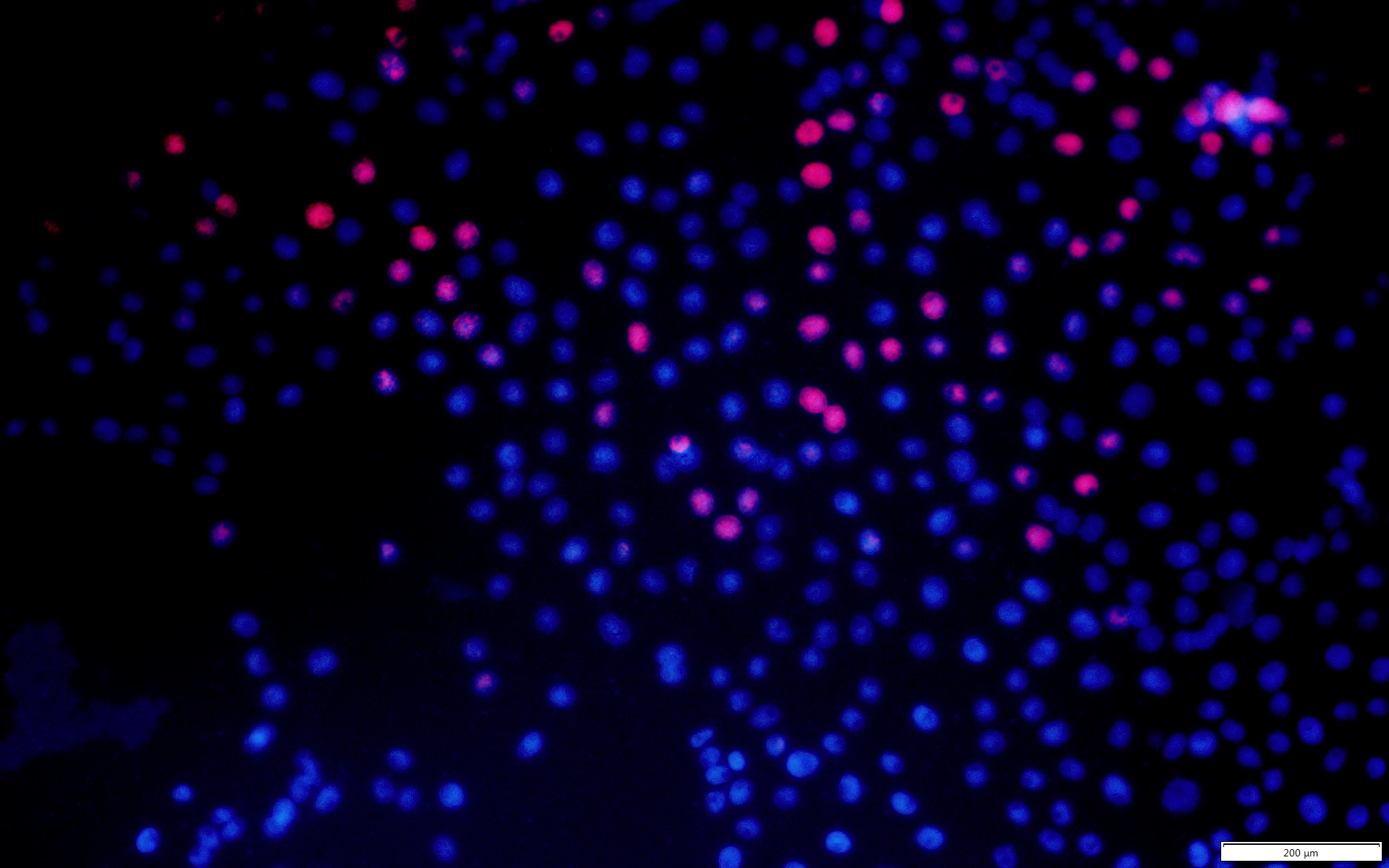

Supplement: Supplementary file 2 [file Data_Sheet_1.zip › raw data/EdU/Figure 7B/mimic+pcDNA (Merge).jpg]

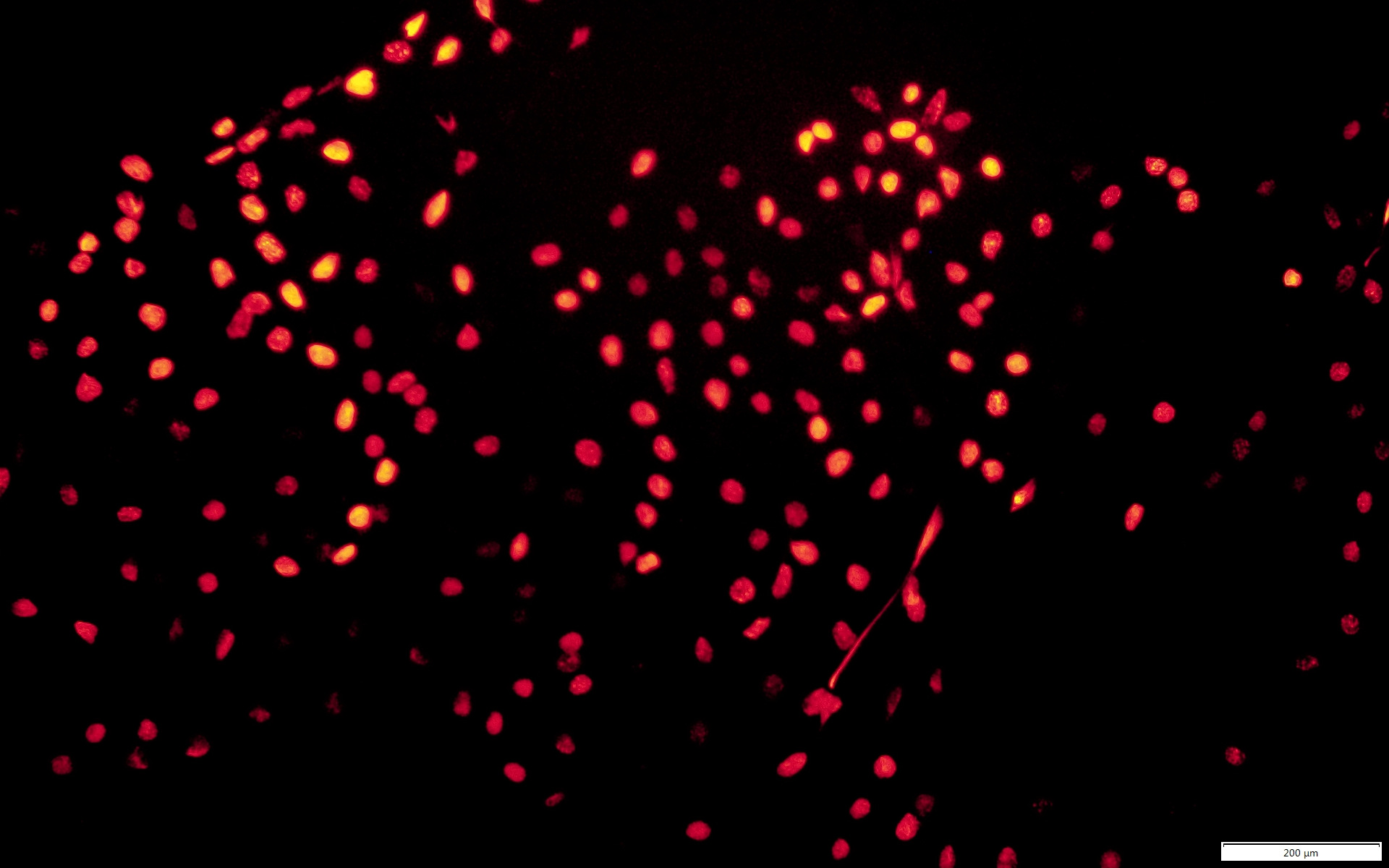

Supplement: Supplementary file 2 [file Data_Sheet_1.zip › raw data/EdU/Figure 7B/mimic+pc-PSME3 (EdU).jpg]

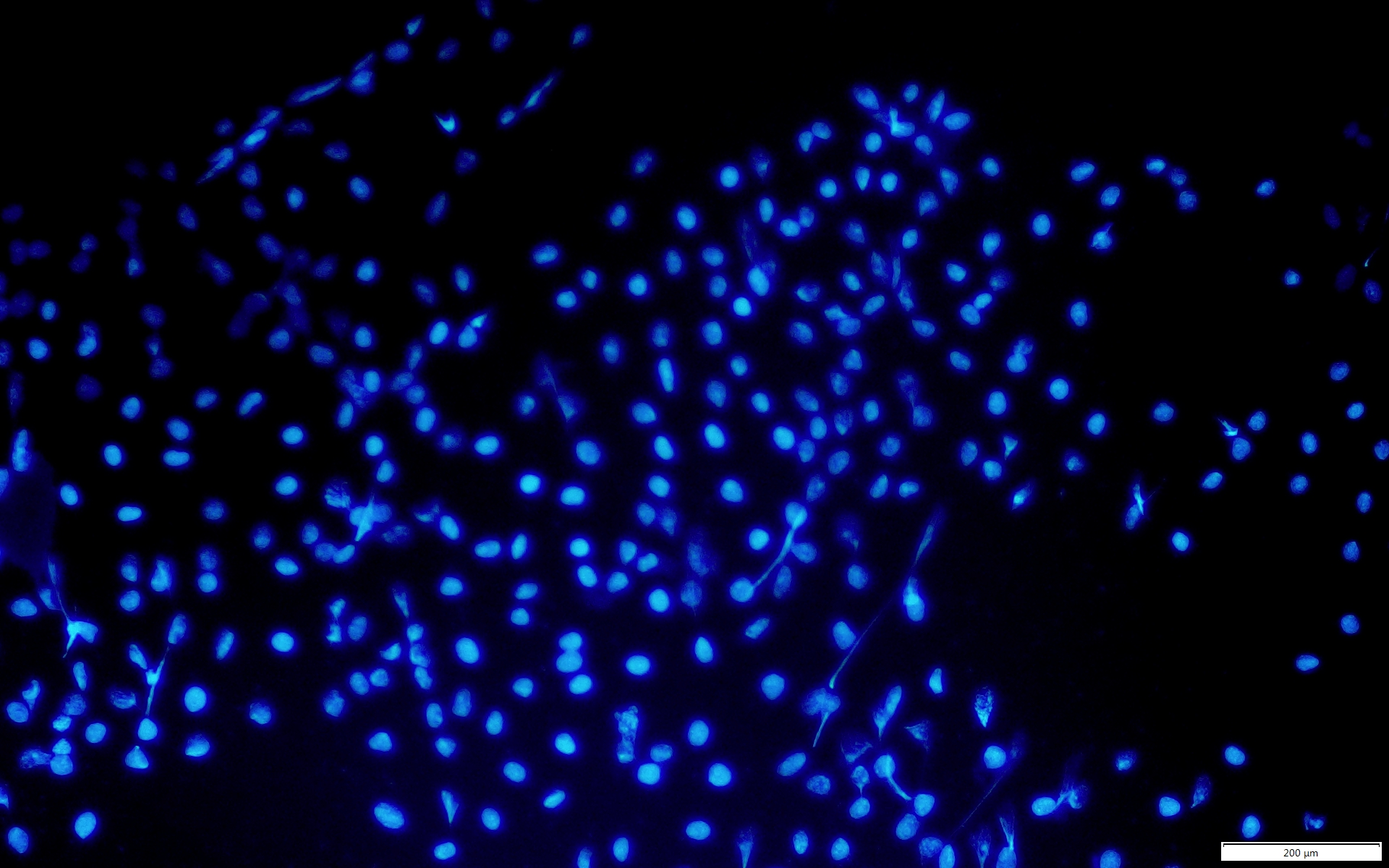

Supplement: Supplementary file 2 [file Data_Sheet_1.zip › raw data/EdU/Figure 7B/mimic+pc-PSME3 (Hoechst).jpg]

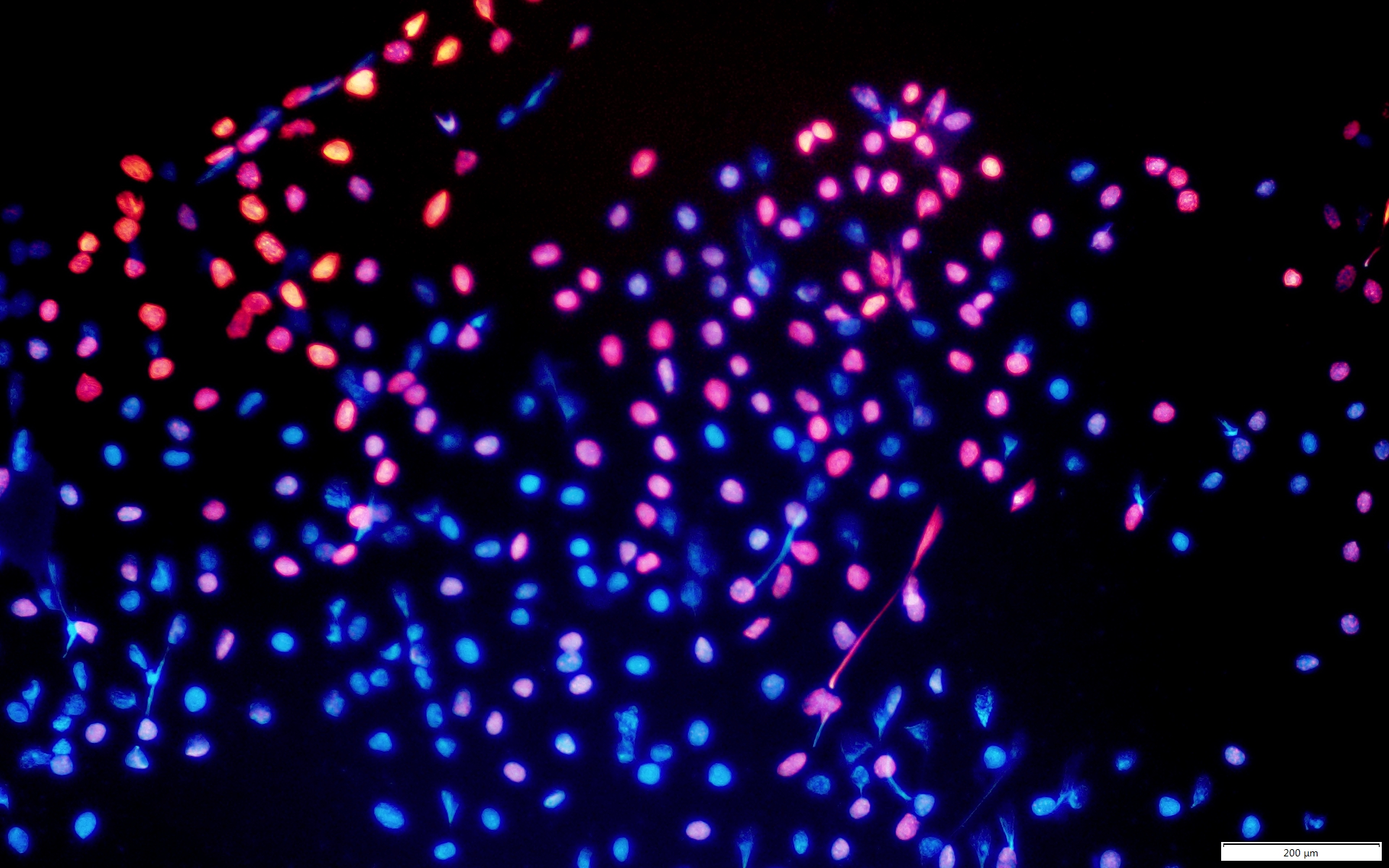

Supplement: Supplementary file 2 [file Data_Sheet_1.zip › raw data/EdU/Figure 7B/mimic+pc-PSME3 (Merge).jpg]
